# A real-world disproportionality analysis of FDA adverse event reporting system (FAERS) events for lecanemab

**DOI:** 10.3389/fphar.2025.1559447

**Published:** 2025-04-02

**Authors:** Linlin Yan, Linhai Zhang, Zucai Xu, Zhong Luo

**Affiliations:** Department of Neurology, Affiliated Hospital of Zunyi Medical University, Zunyi, Guizhou, China

**Keywords:** FAERS, adverse events, AD, lecanemab, signal detection

## Abstract

**Background:**

Lecanemab is a humanized murine IgG1 antibody. Recent Phase 3 clinical trials have demonstrated its ability to reduce brain amyloid-β (Aβ) load and slow cognitive decline in patients with early Alzheimer’s disease (AD). However, since its approval, reports on adverse effects (AEs) associated with lecanemab have been limited. To better understand the AEs related to lecanemab and provide guidance for future clinical use, we analyzed lecanemab-associated AEs using data from the United States Food and Drug Administration (FDA) Adverse Event Reporting System (FAERS).

**Methods:**

We extracted all AEs reports from the FAERS database for the period from the first quarter of 2023 to the third quarter of 2024. Using the Reporting Odds Ratio (ROR), Proportional Reporting Ratio (PRR), Bayesian Confidence Propagation Neural Network (BCPNN), and Multi-item Gamma Poisson Shrinker (MGPS) algorithms, we conducted a comprehensive analysis of lecanemab-related AEs, restricting the analysis to AEs with the role code of primary suspect (PS).

**Results:**

A total of 811 AEs reports related to lecanemab used in AD patients and 506 AEs in Non-AD patients were included. The preferred terms (PTs) identified as positive across all four algorithms included headache, Amyloid Related Imaging Abnormalities-oedema/effusion (ARIA-E), chills, Amyloid Related Imaging Abnormalities-haemosiderosis/microhaemorrhage (ARIA-H), fatigue, infusion-related reaction, nausea, pyrexia, pain, influenza like illness, and so on. Among these, ARIA-E, ARIA-H, brain oedema and status epilepticus were associated with Important Medical Events (IMEs) for AD patients, and brain oedema, cerebral haemorrhage, cerebral microhaemorrhage, subdural haematoma, ischaemic stroke, cerebral infarction were associated with IMEs for Non-AD patients. At the system organ class (SOC) level, the highest signal detection for lecanemab was observed in nervous system disorders among AD and Non-AD patients [ROR for AD: 2.42 (2.2–2.65); ROR for Non-AD: 6.97 (6.12–7.95)]. The median time to the occurrence of these AEs was 44 days after administration in AD patients and 30 days for Non-AD patients.

**Conclusion:**

This study utilized the FAERS database to evaluate lecanemab-associated AEs in AD and non-AD patients, along with their temporal patterns post-marketing authorization, thereby establishing a foundation for subsequent clinical pharmacovigilance. A biweekly 10 mg/kg was identified as the optimal therapeutic dosage. ARIA emerged as frequent treatment-related AEs, with APOEɛ4 carriers demonstrating heightened susceptibility. This necessitates serial brain MRI surveillance for all patients during treatment, aimed not only at early ARIA detection but also vigilant monitoring of IMEs including cerebral haemorrhage, cerebral microhaemorrhages, subdural haematoma, cerebral edema, ischaemic stroke, and cerebral infarction. While AD patients predominantly exhibited non-specific clinical manifestations, non-AD cohorts showed elevated risks of stroke-related complications. Consequently, dynamic neurological deficit monitoring is indispensable for non-AD populations receiving lecanemab to mitigate adverse outcomes. Finally, comprehensive reassessment of anticoagulant or antiplatelet therapy indications is warranted in both AD and non-AD patients to reduce hemorrhagic risks.

## 1 Introduction

AD is the leading cause of dementia, and with the increasing life expectancy of populations, its prevalence is rising annually. In 2018, the global number of dementia cases was approximately 50 million, and this number is expected to double by 2050 (Scheltens et al., 2021). According to the 2016 statistics from the Alzheimer’s Association in the United States, AD became the fifth leading cause of death among individuals aged 65 and older. The care and healthcare costs associated with this disease impose a significant economic burden on families (Alzheimer’s, 2016). Genetic studies suggest that the risk of developing AD is strongly influenced by genetic factors (Gatz et al., 2006), with genes such as those in the *APOE* family playing a key role in its pathogenesis. The *APOE*ɛ4 allele is most significantly associated with late-onset AD and exhibits a dose-dependent effect, while the *APOE*ɛ2 allele can reduce the risk of developing AD (Serrano-Pozo et al., 2021). From a histopathological perspective, AD is characterized by the presence of Aβ plaques and tau neurofibrillary tangles in the brain. The pathological biological function of *APOE* is closely linked to Aβ; in model mice, the knockout of the endogenous *APOE* gene alters the morphology of Aβ, and the *APOE*ɛ4 allele slows down the hydrolysis of Aβ proteins, further accelerating the progression of AD (Raulin et al., 2022).

Current treatments for AD primarily target cholinergic and glutamatergic neurotransmitter pathways, The existence of a national registry also provides opportunities for guidance in clinical practice (Rabinovici et al., 2022). However, these medications only alleviate symptoms and do not offer a cure. Recent studies have highlighted the significance of monoclonal antibodies in AD therapy. In July 2023, the FDA approved lecanemab, marking the advent of a novel AD treatment targeting Aβ through biologics. Lecanemab is a humanized immunoglobulin that binds to amyloid-beta oligomers. A study on early-stage AD demonstrated that lecanemab reduces amyloid-beta biomarkers and improves cognitive function (van Dyck et al., 2023). Adverse reactions reported in its Phase 3 trials include infusion-related reactions, ARIA with edema or effusions, headaches, falls, and urinary tract infections.

The FAERS is an open-access, publicly available database designed to support the FDA’s post-marketing surveillance program for all approved drugs and therapeutic biologics. It contains comprehensive information on adverse events and medication errors collected by the FDA. The system relies on voluntary reporting from consumers, healthcare professionals, and drug manufacturers to capture drug-related adverse events. Data from the FAERS database can be used to establish reliable associations between drugs and adverse events. Currently, there is a lack of research on lecanemab-related adverse events in the FAERS database. This study aims to utilize the database to elucidate lecanemab-associated adverse events and provide further guidance for clinical use.

## 2 Methods

### 2.1 Data source

The FAERS database is updated quarterly, and users can access it completely free of charge at https://fis.fda.gov/extensions/FPD-QDE-FAERS/FPD-QDE-FAERS.html. Files obtained from the FAERS database include demographic information, drug data, outcome statistics, report sources, treatment durations, indications, and adverse event codes. For our study, we included all reports related to lecanemab uploaded to the FAERS database since its approval. To eliminate duplicate data, we screened the DEMO table using PRIMARYID, CASEID, and FDA_DT. If the same CASEID appeared multiple times, the record with the latest FDA_DT was selected. When both CASEID and FDA_DT were identical, we selected the record based on PRIMARYID.

### 2.2 Data acquisition and processing

The FAERS database includes two variables for drugs: DRUGNAME and PROD_AI. To avoid missing data due to the omission of either brand names or generic names, we included the following search terms: “BAN2401”, “LECANEMAB”, “LECANEMAB IRMB”, “LECANEMAB IRMB LEQEMBI” and “LEQEMBI”. In the FAERS database, AEs are categorized based on the Medical Dictionary for Regulatory Activities (MedDRA) as PS (primary suspect), SS (secondary suspect), C (concomitant), and I (interacting) (Kumar, 2019). To improve accuracy, we included only reports with PS as the primary role code. Baseline information such as gender, age, reporting source, region, and year was extracted from AE reports. The interval between EVENT_DT in the DEMO table and START_DT in the THER table was used to assess the time to onset for lecanemab-related AEs.

The Weibull shape parameter (WSP) model was applied to evaluate changes in AE risk over time using the scale (α) and shape (β) parameters. Specifically: β < 1 (95% CI < 1) indicates a decreasing risk over time (early failure); β = 1 (95% CI includes 1) represents a constant risk over time (random failure); β > 1 (95% CI excludes 1) suggests an increasing risk over time (wear-out failure) (Xi et al., 2024).

### 2.3 Data algorithms

We utilized both Frequentist and Bayesian statistical methods to detect drug safety signals. Frequentist statistics included the ROR, PRR, BCPNN, and MGPS algorithms (Park et al., 2020; Liu et al., 2013). The relevant algorithms are detailed in Tables 1, 2.

**TABLE 1:** Two-by-two contingency table for adverse event analysis.

	Number of suspect adverse events	Number of other adverse events	Total
Suspect drug	a	b	a+b
Other drug	c	d	c + d
Total	a+c	b + d	a+b + c + d

**TABLE 2:** Comparison of disproportionality analysis algorithms in pharmacovigilance.

Algorithms	Formula	Prerequisite
ROR	$\text{ROR}=\frac{\left(a/c\right)}{\left(b/d\right)}=\frac{\text{ad}}{\text{bc}}$	lower limit of 95% CI > 1, N ≥ 3
	$95\%\text{CI}=e^{\ln\left(\text{ROR}\right)\pm 1.96\sqrt{\left(\frac{1}{a}+\frac{1}{b}+\frac{1}{c}+\frac{1}{d}\right)}}$	
PRR	$\text{PRR}=\frac{a/\left(a+b\right)}{c/\left(c+d\right)}$	PRR≥2, χ2≥4, N ≥ 3
	$\chi 2=\frac{{\left(\text{ad}-\text{bc}\right)}^{2}}{\left(a+b\right)\left(c+d\right)\left(a+c\right)\left(b+d\right)}$	
BCPNN	$\text{IC}=\log_{2}\frac{a\left(a+b+c+d\right)}{\left(a+c\right)\left(a+b\right)}$	IC025 > 0, N ≥ 3
	$E\left(\text{IC}\right)=\log_{2}\frac{\left(a+\gamma 11\right)\left(a+b+c+d+\alpha \right)\left(a+b+c+d+\beta \right)}{\left(a+b+c+d+\gamma \right)\left(a+b+\alpha 1\right)\left(a+c+\beta 1\right)}$ 95%CI = E (IC) ± 2V(IC)^0.5^	
	$V\left(\text{IC}\right)=\frac{1}{{\left(\ln2\right)}^{2}}\left\{\left[\frac{\left(a+b+c+d\right)-\alpha +\gamma -\gamma 11}{\left(\alpha +\gamma 11\right)\left(1+a+b+c+d+\gamma \right)}\right]+\left[\frac{\left(a+b+c+d\right)-\left(a+b\right)+\alpha -\alpha 1}{\left(a+b+\alpha 1\right)\left(1+a+b+c+d+\alpha \right)}\right]+\left[\frac{\left(a+b+c+d\right)-\left(a+c\right)+\beta -\beta 1}{\left(a+c+\beta 1\right)\left(1+a+b+c+d+\beta \right)}\right]\right\}$	
	$95\%\text{CI}=E\left(\text{IC}\right)\pm 2\sqrt{v\left(\text{IC}\right)}$	
MGPS	$\text{EBGM}=\frac{a\left(a+b+c+d\right)}{\left(a+c\right)\left(a+b\right)}$	EBGM05 ≥ 2,N ≥ 3
	$95\%\text{CI}=e^{\ln\left(\text{EBGM}\right)\pm 1.96\sqrt{(\frac{1}{a}+\frac{1}{b}+\frac{1}{c}+\frac{1}{d}}}$	

a, number of reports containing both the suspect drug and the suspect adverse drug reaction; b, number of reports containing the suspect adverse drug reaction with other medications (except the drug of interest); c, number of reports containing the suspect drug with other adverse drug reactions (except the event of interest); d, number of reports containing other medications and other adverse drug reactions. ROR, reporting odds ratio; CI, confidence interval; N, the number of co-occurrences; PRR, proportional reporting ratio; χ2, chi-squared; BCPNN, bayesian confidence propagation neural network; IC, information component; IC025, the lower limit of the 95% CI, of the IC; E (IC), the IC, expectations; V (IC), the variance of IC; MGPS, multi-item gamma Poisson shrinker; EBGM, empirical Bayesian geometric mean; EBGM05, the lower limit of the 95% CI, of EBGM.

### 2.4 Statistical analysis

This study employed four commonly used algorithms—ROR, PRR, BCPNN, and MGPS—to evaluate the association between the target drug and AEs. The ROR is a disproportionality measure that estimates the likelihood of a specific AE occurring in reports associated with a particular drug compared to all other drugs, using logistic regression analysis. The ROR algorithm considers the total number of reports and can be adjusted for potential confounding variables. Similar to ROR, the PRR is another algorithm used to detect potential drug-related safety signals. PRR identifies signals by comparing the proportion of reports of a specific AE associated with a particular drug to the proportion of the same AE associated with all other drugs. The BCPNN is an advanced Bayesian-based algorithm that evaluates the likelihood of a causal relationship between a drug and an AE. BCPNN is particularly effective in handling sparse data and generates fewer false-positive signals compared to PRR. This algorithm operates by propagating evidence strength through a network of interconnected nodes, continuously updating the probabilities of associations based on cumulative evidence. The MGPS is a shrinkage method similar to PRR or ROR, designed to minimize false-positive signals. MGPS applies a gamma distribution to observed counts and adjusts the estimates towards a central value (typically zero) to shrink signals. This approach highlights the most probable signals, providing focus for subsequent investigations. Each algorithm has distinct strengths and limitations, and their selection depends on the required balance between sensitivity and specificity for signal detection (Zhao and Tao, 2024).

## 3 Results

### 3.1 Clinical characteristics

**FIGURE 1:**
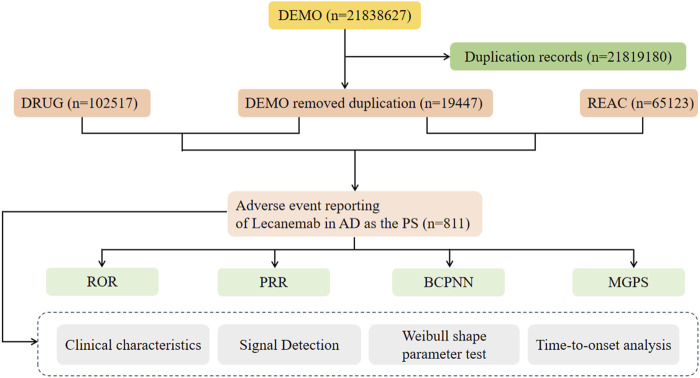
Flowchart of this study.

Figure 1 illustrates the workflow of data extraction, processing, and analysis in this study. From the third quarter of 2023 to the present, the FAERS database has recorded a total of 21,838,627 cases. After removing 21,819,180 duplicate cases, 19,447 cases were included, comprising 65,123 AEs records. The clinical characteristics of these drugs among AD patients are summarized in Table 3, and Non-AD in Supplementary Table 1.

**TABLE 3:** Clinical characteristics of lecanemab in AD.

Characteristics	Lecanemab (N, %)
Total Number of Reports	811
Gender	
Female	475 (58.6%)
Male	317 (39.1%)
Unknow	19 (2.3%)
Weight	
<50 Kg	17 (2.1%)
50-100 Kg	228 (28.1%)
>100 Kg	24 (3%)
Unknow	542 (66.8%)
Age	
<65	101 (12.4%)
65–85	601 (74.1%)
>85	22 (2.7%)
Unknow	87 (10.7%)
Report Person	
Consumer (CN)	418 (51.5%)
Health Professional (HP)	138 (17%)
Physician (MD)	223 (27.5%)
Pharmacist (PH)	16 (2%)
Unknow	16 (2%)
Serious outcome	
Death (DE)	25 (3.1%)
Disability (DS)	1 (0.1%)
Hospitalization (HO)	133 (16.4%)
Life-threatening (LT)	12 (1.5%)
Other Serious Outcomes (OT)	63 (7.8%)
Required Intervention (RI)	2 (0.2%)
Unknow	575 (70.9%)
Report Countries (Top Seven)	
United States	734 (90.5%)
Japan	43 (5.3%)
China	11 (1.4%)
France	4 (0.5%)
Italy, United Kingdom, Korea (South)	3 (0.4%), respectively

Among the reports related to lecanemab in AD patients, a total of 811 cases were identified, including 475 female cases (58.6%) and 317 male cases (39.1%). Besides, for Non-AD patients, 506 cases were identified, including 240 female cases (47.4%) and 173 male cases (34.2%). Across various subgroups, the majority of AE reports were from individuals weighing 50–100 kg and aged 65–85 years both in AD and Non-AD. Reports were predominantly submitted by consumers and healthcare professionals, who provided reliable information on drug-related AEs. The most common outcome was hospitalization, accounting for 16.4% and 9.9% for AD and Non-AD patients.

The top seven countries reporting AEs related to lecanemab are the United States (90.5%), Japan (5.3%), China (1.4%), France (0.5%), and the United Kingdom, Italy, and South Korea, each accounting for 0.4%. Since lecanemab has been on the market for only 1 year, 175 AE cases were reported in 2023, while 636 cases were reported in 2024 for AD patients.

### 3.2 Signal detection

After screening positive signals through ROR, PRR, MGPS, and BCPNN algorithms, a total of 24 AEs were identified. Table 4 lists four algorithms positive AEs under the PTs in AD, with ARIA-E, ARIA-H, brain oedema and status epilepticus associated with IMEs, Supplementary Table 2 lists four algorithms positive AEs under the PTs in Non-AD, and with ARIA-E, ARIA-H, brain oedema, cerebral haemorrhage, cerebral microhaemorrhage, subdural haematoma, ischaemic stroke and cerebral infarction associated with IMEs, highlighted in bold.

**TABLE 4:** PTs of lecanemab among AD identified as positive across all four algorithms.

PT	N	RR (95%CI)	ROR (95%Cl)	X^2^	IC(IC025)	EBGM(EBGM05)
Headache	170	14.79 (14.61–14.97)	16.07 (13.32–19.4)	1,500.82	10.39 (8.88)	3.38 (3.12)
**ARIA-E**	99	21.27 (21.02–21.52)	22.33 (17.23–28.92)	1,146.94	13.11 (10.56)	3.71 (3.37)
Chills	96	53.18 (52.86–53.51)	55.83 (40.1–77.72)	1835.61	20.44 (15.5)	4.35 (3.97)
**ARIA-H**	90	27.07 (26.79–27.35)	28.3 (21.28–37.64)	1,220.26	15.04 (11.84)	3.91 (3.54)
Fatigue	89	7.22 (7–7.45)	7.52 (5.94–9.5)	391.34	6.07 (4.98)	2.6 (2.26)
Infusion Related Reaction	65	293.23 (292.45–294.01)	303.08 (138.8–661.82)	1842.47	29.41 (15.3)	4.88 (4.38)
Nausea	60	2.78 (2.52–3.04)	2.84 (2.17–3.71)	63.68	2.64 (2.11)	1.4 (1.01)
Pyrexia	50	4.52 (4.23–4.82)	4.61 (3.42–6.23)	120.8	4.08 (3.18)	2.03 (1.6)
Pain	28	4.89 (4.49–5.28)	4.94 (3.31–7.37)	75.17	4.36 (3.12)	2.13 (1.55)
Influenza Like Illness	21	73.68 (72.9–74.46)	74.46 (34.06–162.78)	451.9	22.8 (11.85)	4.51 (3.7)
Amyloid Related Imaging Abnormalities	18	43.72 (43.01–44.44)	44.11 (21.58–90.16)	315.27	18.92 (10.4)	4.24 (3.4)
Feeling Cold	15	22.56 (21.89–23.22)	22.72 (11.69–44.14)	180.35	13.57 (7.79)	3.76 (2.9)
Nasopharyngitis	13	6.32 (5.72–6.91)	6.35 (3.5–11.54)	48.53	5.43 (3.29)	2.44 (1.6)
COVID-19	12	13.07 (12.4–13.74)	13.14 (6.7–25.79)	94.64	9.54 (5.42)	3.25 (2.34)
Migraine	9	12.36 (11.59–13.13)	12.41 (5.73–26.85)	67.55	9.16 (4.8)	3.2 (2.15)
**Brain Oedema**	9	11.84 (11.08–12.61)	11.89 (5.52–25.62)	65.01	8.89 (4.68)	3.15 (2.11)
Brain Fog	7	44.21 (43.06–45.36)	44.36 (14.07–139.9)	123.2	19 (7.27)	4.25 (2.95)
Visual Impairment	7	6.91 (6.09–7.72)	6.93 (3.05–15.72)	29.04	5.85 (2.95)	2.55 (1.43)
Superficial Siderosis Of Central Nervous System	5	7.18 (6.21–8.15)	7.19 (2.72–19.01)	21.67	6.03 (2.67)	2.59 (1.29)
Flushing	5	7.18 (6.21–8.15)	7.19 (2.72–19.01)	21.67	6.03 (2.67)	2.59 (1.29)
Nasal Congestion	4	14.03 (12.86–15.21)	14.06 (4.33–45.7)	33.53	10.02 (3.74)	3.33 (1.82)
**Status Epilepticus**	4	5.74 (4.68–6.81)	5.75 (1.98–16.7)	13.26	5.01 (2.05)	2.33 (0.92)
Sneezing	3	23.68 (22.19–25.18)	23.72 (5.3–106.05)	37.25	13.96 (3.99)	3.8 (2.04)
Magnetic Resonance Imaging Abnormal	3	94.74 (92.47–97)	94.88 (9.86–912.54)	69.56	24.43 (3.68)	4.61 (2.67)

**FIGURE 2:**
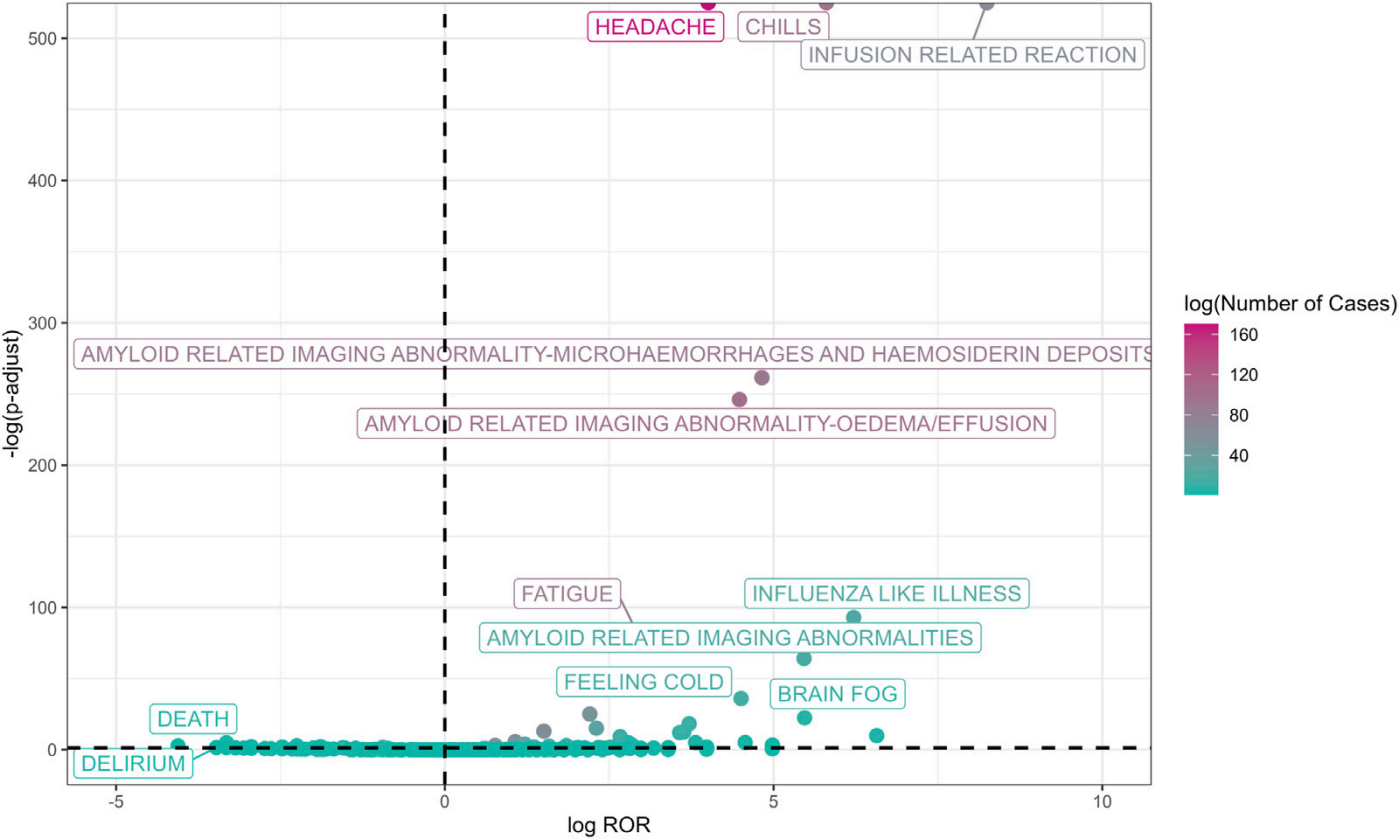
The volcano picture of the PT in lecanemab used for AD patients.

Figure 2 illustrates all 381 PTs related to lecanemab in AD patients (Supplementary Table 3), and Supplementary Figure S1 illustrates all 225 PTs in Non-AD patients (Supplementary Table 4). The X-axis represents log_2_ROR, while the Y-axis indicates the negative logarithm of the P-value adjusted using the false discovery rate (FDR) method. Points closer to the right side of the X-axis indicate stronger relevance for specific AEs, and positive values on the Y-axis signify statistical significance. Therefore, AE records located in the upper-right corner represent signals with the strongest intensity and statistical significance. The color of the points reflects the number of cases, with redder points indicating a higher case count.

**FIGURE 3:**
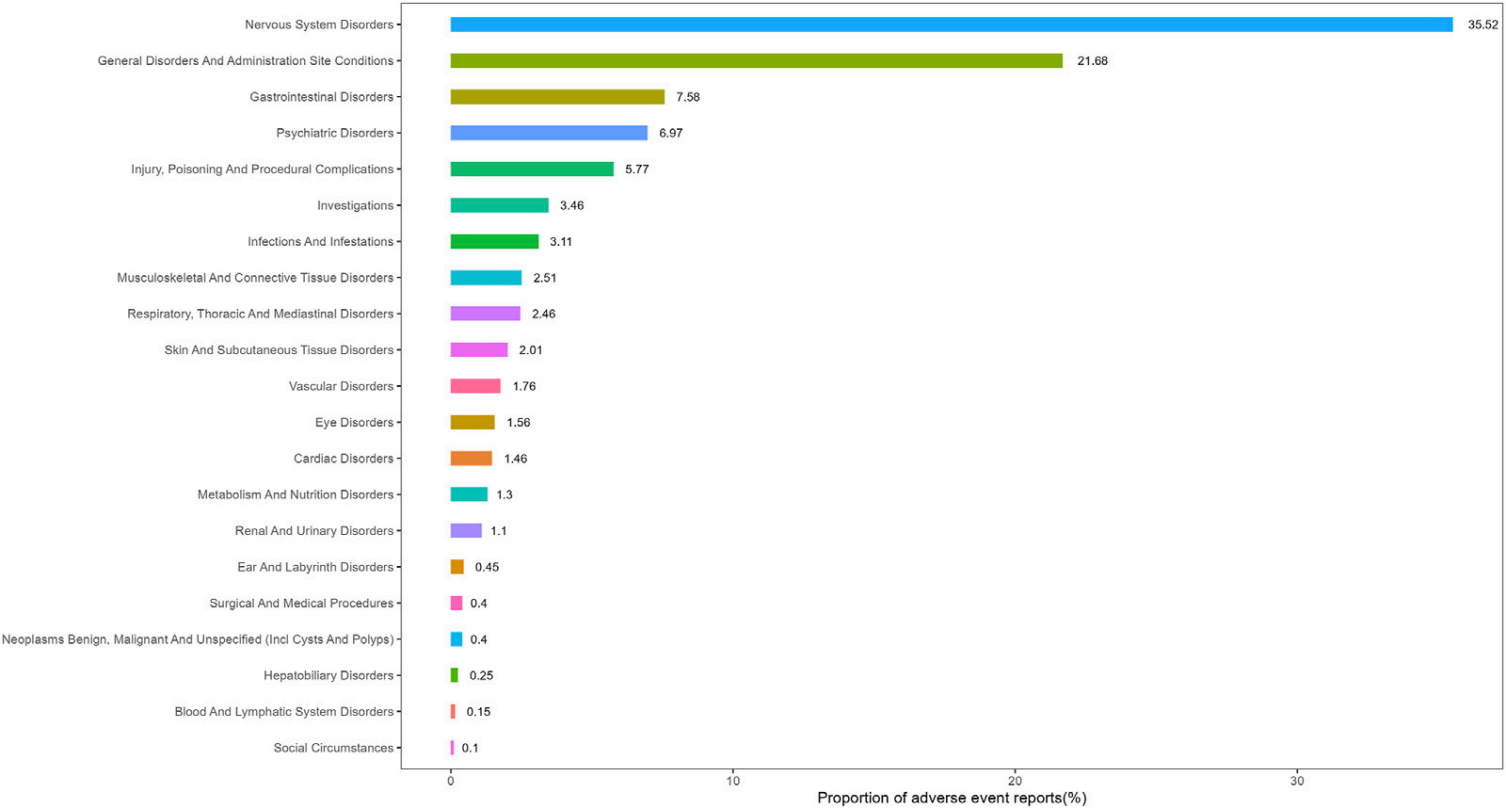
The percentage of the AE at SOC level in lecanemab used for AD patients.

**FIGURE 4:**
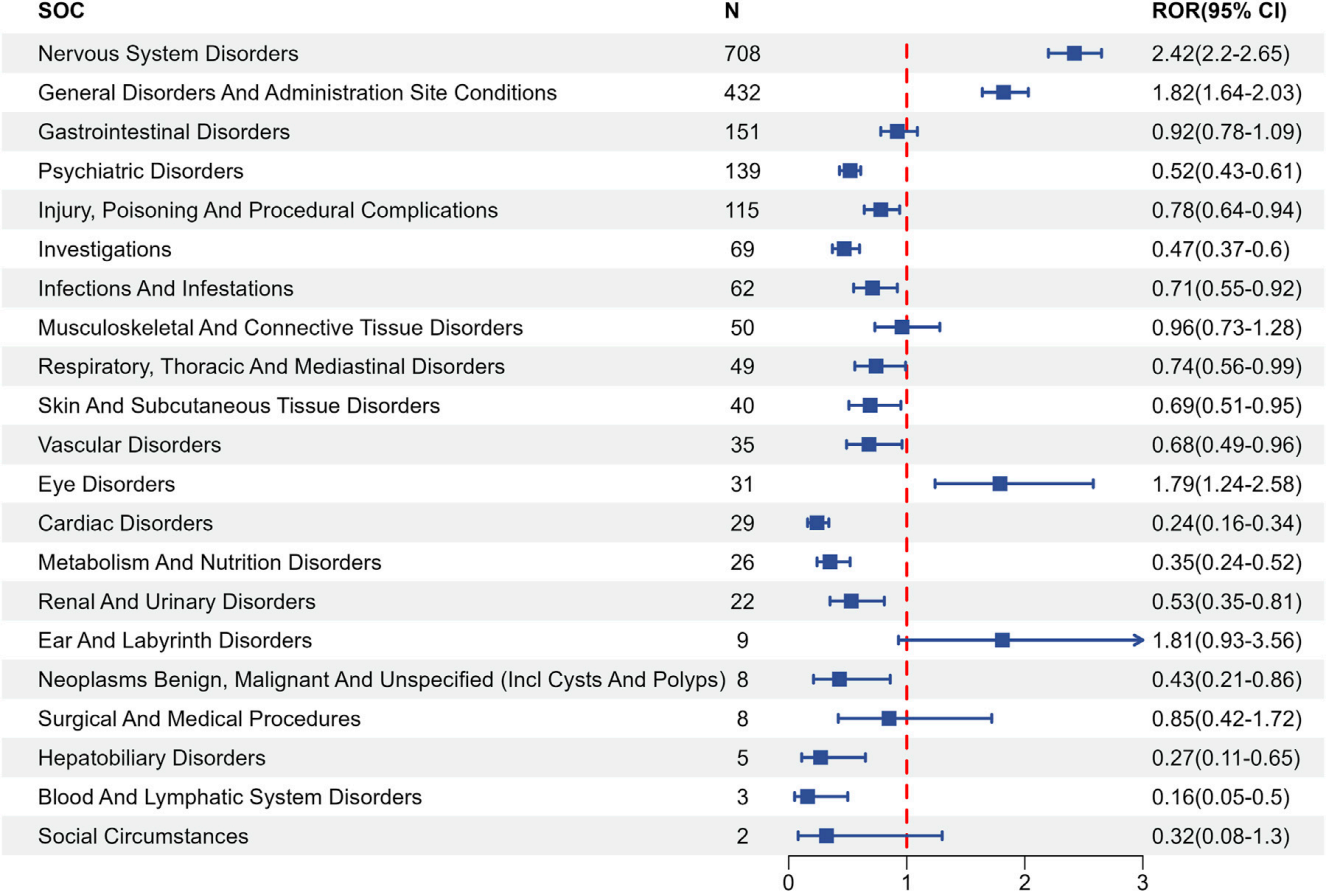
The forest picture of the SOC level in lecanemab used for AD patients.

Figures 3, 4 present the proportion of all positive AE signals for AD patients classified by SOC and the corresponding forest plots, and Supplementary Figures S2, 3 present the same results for Non-AD patients. Notable positive signals were observed in the categories of nervous system disorders, this results suggesting a significant association between lecanemab and related AEs for all patients.

### 3.3 Time to onset of lecanemab related PT and weibull shape parameter test

**FIGURE 5:**
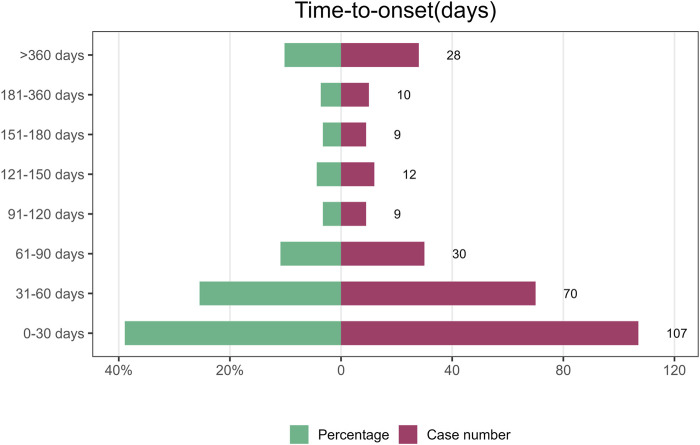
The time of AE onset in lecanemab used for AD patients.

Data extracted from the FAERS database were analyzed to examine the temporal characteristics of lecanemab-associated AEs using a Weibull distribution. The median time to onset of lecanemab-related AEs in AD was 44 days after administration (interquartile range [IQR]: 14.00–87.5 days) (Table 5) and 30 days for Non-AD (interquartile range [IQR]: 14.00–59 days) (Supplementary Table 5). Overall, lecanemab-related AEs predominantly occurred in the early stages, most of all AEs reported within 90 days of administration. However, AE reports continued to appear even beyond 1 year post-administration (Figure 5; Supplementary Figure S4).

**TABLE 5:** Time-to-onset analysis of Lecanemab related AEs signals using the Weibull distribution test in AD patients.

				Weibull Distribution				
	Time to onset (days)			Scale Parameter		Shape Parameter		
Drug	N	Median (IQR)	Min-Max	α	95% CI	β	95% CI	Failure Type
Lecanemab	275	44 (14.00–87.5)	1–1,283	81.05	63.58–98.52	0.58	0.53–0.63	Early Failure

To further evaluate the temporal characteristics of lecanemab-associated AEs, the Weibull analysis yielded a shape parameter of 0.58 (95% CI: 0.53–0.63). This indicates that the likelihood of lecanemab-associated AEs decreases gradually over time in AD patients.

## 4 Discussion

This study utilized the FAERS database to conduct a real-world, non-clinical investigation, describing the AEs associated with lecanemab since its market approval and their temporal trends. In previous AD treatments, the primary focus was on the cholinergic and glutamatergic systems, which aimed to slow the progression of AD by modulating acetylcholine and glutamate levels in the brain. However, these approaches have never been curative, and the risk of AEs increases with higher drug dosages (Passeri et al., 2022). The deposition of Aβ in the brain is a hallmark biomarker and a critical pathological feature of AD (Ali et al., 2023). It also serves as a key target in AD pathogenesis research and drug development. Previous studies at the animal level have provided evidence supporting the efficacy of immunotherapy in improving memory and behavioral outcomes (Gallardo and Holtzman, 2017), Recent studies have indicated that monoclonal antibodies can specifically target and clear Aβ in the brain (van Dyck et al., 2023; Budd Haeberlein et al., 2022; Mintun et al., 2021), improving clinical symptoms in patients with early-stage AD. These findings provide critical clinical evidence for both the treatment and pathogenesis of AD.

Lecanemab, also known as Leqembi, was officially approved by the FDA in July 2023 as a treatment for early-stage AD. Lecanemab is a humanized murine IgG1 monoclonal antibody that significantly enhances binding to soluble Aβ protofibrils (Logovinsky et al., 2016), which are highly neurotoxic and a primary cause of AD pathogenesis. Results from an 18-month Phase 3 clinical trial demonstrated that lecanemab can reduce brain amyloid-beta biomarkers and improve cognitive function at the 18-month mark (van Dyck et al., 2023). In the 2021 Phase 2b double-blind trial and the 2023 Phase 3 clinical trial, the most common serious AEs was infusion-related reactions, which were dose-dependent. However, most patients experienced no reaction after receiving prophylactic symptomatic treatment with related medications (van Dyck et al., 2023; Swanson et al., 2021).

However, clinical research on the AEs associated with lecanemab remains limited. To enhance reliability and reduce the risk of false positives, we focused on PT signals identified as positive across four algorithms and adverse events categorized by SOC. We hope our study provides guidance for clinical application, improves patients’ quality of life, and enhances clinical outcomes.

Our findings indicate that, in addition to infusion-related reactions, AEs such as headache, chills, cerebral microhaemorrhage, fatigue, dizziness, and nausea were consistent with literature. These manifestations may represent either non-specific symptoms associated with monoclonal antibody therapy or adverse reactions linked to ARIA (Roytman et al., 2023; Cummings et al., 2023). ARIA refers to MRI signal abnormalities that may occur spontaneously or as treatment-related AEs when using monoclonal antibodies targeting Aβ. These abnormalities are classified into two types: ARIA-E and ARIA-H. While the precise mechanism underlying ARIA remains unclear, studies suggest it may be associated with vascular damage and leakage caused by the binding of monoclonal antibodies to Aβ (Barakos et al., 2022; Sperling et al., 2011). ARIA is often asymptomatic and typically resolves within weeks to months after discontinuing treatment (Sperling et al., 2012; Filippi et al., 2022), but the FDA has mandated a black box warning for lecanemab, emphasizing persistent risks associated with ARIA (Mahase, 2023), Consequently, stringent post-therapeutic monitoring is imperative. Per the Appropriate Use Recommendations, serial MRI surveillance should be conducted following the fifth, seventh, and 14th infusions, with an additional scan mandated after the 26th infusion—particularly for *APOE* ε4 carriers and patients exhibiting ARIA on early MRI (Cummings et al., 2023).

Clinical trial data indicate that ARIA begin to subside approximately 3 months after onset. The presence of the *APOE* ε4 gene is a major risk factor for both ARIA-E and ARIA-H, therefore, it is recommended to complete genetic testing before patients start lecanemab treatment. Due to the similar pathogenesis of ARIA and cerebral amyloid angiopathy, corticosteroids may be effective in managing ARIA (Roytman et al., 2023). Recently, a case in Japan reported a patient with early-stage AD who developed severe ARIA-H and ARIA-E following lecanemab treatment (Yamazaki et al., 2025). After receiving regular corticosteroid therapy, the patient’s ARIA-E showed significant improvement.

A meta-analysis included five studies that investigated the use of lecanemab for the treatment of AD in individuals aged 70–72 years (Arroyo-Pacheco et al., 2025), among the various dosing regimens, a dose of 10 mg/kg administered biweekly was identified as the optimal dosage. Under this regimen, the efficacy at 12 and 18 months was 97.5% and 97.7% higher than that of the placebo, respectively. Moreover, across all statistical sensitivity models, this dosage was associated with a reduced rate of clinical progression—as measured by the AD Composite Score (ADCOMS), the Clinical Dementia Rating Scale Sum of Boxes (CDR-SB), and the AD Assessment Scale–Cognitive Subscale (ADAS-Cog)—at 18 months (Dhadda et al., 2022). Another meta-analysis highlighted that lecanemab was particularly effective in improving the CDR-SB (Cao et al., 2025). In addition, the use of lecanemab in patients receiving anticoagulants was associated with an increased risk of bleeding; previous studies have demonstrated that the concurrent use of anticoagulants, antiplatelet agents, or antithrombotics during anti-amyloid treatment increases the risk of ARIA-H (Hampel et al., 2023).

These findings suggest that patients at high risk of bleeding or those requiring anticoagulant therapies should undergo comprehensive risk assessments during treatment, and patients with coagulation disorders should be excluded. In some cases, treatment discontinuation may be necessary to mitigate these risks. Notably, the recommendations allowed lecanemab participants to receive aspirin therapy (up to 325 mg/day) or other antiplatelet medications (Cummings et al., 2023).

Time-to-onset analysis revealed that the majority of AEs occurred within the first 3 months both in AD and Non-AD patients. Furthermore, the majority of AEs in both AD and Non-AD patients corresponded to the Nervous System Disorders SOC category [ROR = 2.42 (95% CI 2.2–2.65) for AD vs 6.97 (95% CI 6.12–7.95) for non-AD, suggesting a potentially elevated risk of lecanemab-associated AEs in Non-AD patients, and Non-AD patients demonstrated additional high-weight adverse events, including cerebral microhaemorrhage, subcortical stroke, and superficial siderosis of central nervous system, alongside ARIA and infusion-related reactions. These findings underscore the necessity for multifaceted vigilance when administering lecanemab to non-AD populations. Notably, AD patients treated with lecanemab demonstrated IMEs such as ARIA-E, ARIA-H, cerebral oedema, and status epilepticus. In contrast, non-AD patients manifesting intracerebral haemorrhage, cerebral microhaemorrhages, subdural haematoma, ischaemic stroke, and cerebral infarction, alongside ARIA and cerebral oedema. Critically, stroke-related adverse events displayed significantly higher incidence in non-AD cohorts.

Additionally, the disproportionately high number of reports from the United States is related to the high use of lecanemab and first approval in the United States. Therefore, we still need to further include data from other countries or regions to enrich the conclusions, decentralized clinical trial protocols can provide more professional advice for future drug trials (Howard et al., 2024). In our study, we focused on PT signals that were positive across all four algorithms, allowing us to identify more reliable indicators. The FAERS database, a large-scale platform enabling spontaneous reporting of AEs, offers the advantage of collecting AEs from multiple dimensions. However, the spontaneous reporting mechanism may result in duplicate cases, underreporting, and incomplete data, which could affect the accuracy of AE evaluation and limit the ability to establish a definitive causal relationship between reported AEs and drug use. Furthermore, inherent limitations of the database prevented the acquisition of the denominator, thereby impeding the calculation of incidence rates.

Despite these limitations, our study provides a comprehensive analysis of post-marketing AEs associated with lecanemab. This large-scale data-driven approach contributes valuable insights for clinical monitoring and risk identification. Special attention should be given to AEs occurring within the first 3 months of lecanemab use and AEs of Non-AD patients.

## 5 Conclusion

We systematically reviewed the FAERS database to evaluate the risk of lecanemab-associated AEs and the timing of their occurrence. These results suggest prioritizing monitoring for central nervous system-related AEs associated with lecanemab, as well as closely following up with patients within the first 3 months of treatment. These findings provide valuable insights to inform clinical decision-making and drug surveillance.

## Data Availability

The original contributions presented in the study are included in the article/Supplementary Material, further inquiries can be directed to the corresponding authors.

## References

[B1] Ali D. Khouli R. H. E. Abner E. L. Bahrani A. A. Gold B. T. Jiang Y. (2023), The combined effect of β -amyloid and white matter hyperintensities on present and future executive function performance in cognitively normal older adults. Alzheimer s Dement. 19: e061863. 10.1002/alz.061863

[B2] Alzheimer's A. (2016). 2016 Alzheimer's disease facts and figures. Alzheimers Dement. 12, 459–509. 10.1016/j.jalz.2016.03.001 27570871

[B3] Arroyo-Pacheco N. Sarmiento-Blanco S. Vergara-Cadavid G. Castro-Leones M. Contreras-Puentes N. (2025). Monoclonal therapy with lecanemab in the treatment of mild Alzheimer's disease: a systematic review and meta-analysis. Ageing Res. Rev. 104, 102620. 10.1016/j.arr.2024.102620 39638097

[B4] Barakos J. Purcell D. Suhy J. Chalkias S. Burkett P. Marsica Grassi C. (2022). Detection and management of amyloid-related imaging abnormalities in P atients with Alzheimer's disease treated with anti-amyloid beta therap y. J. Prev. Alzheimers Dis. 9, 211–220. 10.14283/jpad.2022.21 35542992

[B5] Budd Haeberlein S. Aisen P. S. Barkhof F. Chalkias S. Chen T. Cohen S. (2022). Two randomized phase 3 studies of aducanumab in early Alzheimer's dise ase. J. Prev. Alzheimers Dis. 9, 197–210. 10.14283/jpad.2022.30 35542991

[B6] Cao W. Zhu B. Liu Z. Jia X. Zhao H. Gu N. (2025). Comparison of the efficacy of updated drugs for the treatment on the i mprovement of cognitive function in patients with Alzheimer 's disease: a systematic review and network meta-analysis. Neuroscience 565, 29–39. 10.1016/j.neuroscience.2024.11.029 39550061

[B7] Cummings J. Apostolova L. Rabinovici G. D. Atri A. Aisen P. Greenberg S. (2023). Lecanemab: appropriate use recommendations. J. Prev. Alzheimers Dis. 10, 362–377. 10.14283/jpad.2023.30 37357276 PMC10313141

[B8] Dhadda S. Kanekiyo M. Swanson C. J. Irizarry M. Berry S. (2022). Consistency of efficacy results across various clinical measures and s tatistical methods in the lecanemab phase 2 trial of early Alzheimer's disease. Alzheimers Res. Ther. 14, 182. 10.1186/s13195-022-01129-x 36482412 PMC9733166

[B9] Filippi M. Cecchetti G. Spinelli E. G. Vezzulli P. Falini A. Agosta F. (2022). Amyloid-related imaging abnormalities and β-amyloid-targeting antibodi es: a systematic review. JAMA Neurol. 79, 291–304. 10.1001/jamaneurol.2021.5205 35099507

[B10] Gallardo G. Holtzman D. M. (2017). Antibody therapeutics targeting Aβ and tau. Cold Spring Harb. Perspect. Med. 7, a024331. 10.1101/cshperspect.a024331 28062555 PMC5500436

[B11] Gatz M. Reynolds C. A. Fratiglioni L. Johansson B. Mortimer J. A. Berg S. (2006). Role of genes and environments for explaining Alzheimer disease. Arch. Gen. Psychiatry 63, 168–174. 10.1001/archpsyc.63.2.168 16461860

[B12] Hampel H. Elhage A. Cho M. Apostolova L. G. Nicoll J. A. R. Atri A. (2023). Amyloid-related imaging abnormalities (ARIA): radiological, biological and clinical characteristics. Brain 146, 4414–4424. 10.1093/brain/awad188 37280110 PMC10629981

[B13] Howard L. Abdelnour C. Abner E. L. Allegri R. F. Dodge H. H. Gauthier S. (2024). Decentralized clinical trials for medications to reduce the risk of de mentia: Consensus report and guidance. Alzheimers Dement. 20, 4625–4634. 10.1002/alz.13891 38824659 PMC11247660

[B14] Kumar A. (2019). The newly available FAERS public dashboard: implications for Health Ca re professionals. Hosp. Pharm. 54, 75–77. 10.1177/0018578718795271 30923396 PMC6431724

[B15] Liu M. McPeek Hinz E. R. Matheny M. E. Denny J. C. Schildcrout J. S. Miller R. A. (2013). Comparative analysis of pharmacovigilance methods in the detection of adverse drug reactions using electronic medical records. J. Am. Med. Inf. Assoc. 20, 420–426. 10.1136/amiajnl-2012-001119 PMC3628053 23161894

[B16] Logovinsky V. Satlin A. Lai R. Swanson C. Kaplow J. Osswald G. (2016). Safety and tolerability of BAN2401--a clinical study in Alzheimer's di sease with a protofibril selective Aβ antibody. Alzheimers Res. Ther. 8, 14. 10.1186/s13195-016-0181-2 27048170 PMC4822297

[B17] Mahase E. (2023). Alzheimer's disease: lecanemab gets full FDA approval and black box sa fety warning. BMJ 382, 1580. 10.1136/bmj.p1580 37419629

[B18] Mintun M. A. Lo A. C. Duggan Evans C. Wessels A. M. Ardayfio P. A. Andersen S. W. (2021). Donanemab in early Alzheimer's disease. N. Engl. J. Med. 384, 1691–1704. 10.1056/NEJMoa2100708 33720637

[B19] Park G. Jung H. Heo S.-J. Jung I. (2020). Comparison of Data Mining Methods for the Signal Detection of Adverse Drug Events with a Hierarchical Structure in Postmarketing Surveillanc e. Life (Basel) 10, 138. 10.3390/life10080138 32764444 PMC7460123

[B20] Passeri E. Elkhoury K. Morsink M. Broersen K. Linder M. Tamayol A. (2022). Alzheimer's disease: treatment strategies and their limitations. Int. J. Mol. Sci. 23, 13954. 10.3390/ijms232213954 36430432 PMC9697769

[B21] Rabinovici G. D. Rafii M. S. Apgar C. Barakos J. Brangman S. A. (2022). ALZ-NET: using real world evidence to inform the future of Alzheimer’s treatment and care. Alzheimer's. and Dementia 18. 10.1002/alz.069542

[B22] Raulin A.-C. Doss S. V. Trottier Z. A. Ikezu T. C. Bu G. Liu C. C. (2022). ApoE in Alzheimer's disease: pathophysiology and therapeutic strategie s. Mol. Neurodegener. 17, 72. 10.1186/s13024-022-00574-4 36348357 PMC9644639

[B23] Roytman M. Mashriqi F. Al-Tawil K. Schulz P. E. Zaharchuk G. Benzinger T. L. S. (2023). Amyloid-related imaging abnormalities: an update. AJR Am. J. Roentgenol. 220, 562–574. 10.2214/AJR.22.28461 36321981

[B24] Scheltens P. De Strooper B. Kivipelto M. Holstege H. Chételat G. Teunissen C. E. (2021). Alzheimer's disease. Lancet 397, 1577–1590. 10.1016/S0140-6736(20)32205-4 33667416 PMC8354300

[B25] Serrano-Pozo A. Das S. Hyman B. T. (2021). APOE and Alzheimer's disease: advances in genetics, pathophysiology, a nd therapeutic approaches. Lancet Neurol. 20, 68–80. 10.1016/S1474-4422(20)30412-9 33340485 PMC8096522

[B26] Sperling R. Salloway S. Brooks D. J. Tampieri D. Barakos J. Fox N. C. (2012). Amyloid-related imaging abnormalities in patients with Alzheimer's dis ease treated with bapineuzumab: a retrospective analysis. Lancet Neurol. 11, 241–249. 10.1016/S1474-4422(12)70015-7 22305802 PMC4063417

[B27] Sperling R. A. Jack C. R. Black S. E. Frosch M. P. Greenberg S. M. Hyman B. T. (2011). Amyloid-related imaging abnormalities in amyloid-modifying therapeutic trials: recommendations from the Alzheimer's Association Research Rou ndtable Workgroup. Alzheimers Dement. 7, 367–385. 10.1016/j.jalz.2011.05.2351 21784348 PMC3693547

[B28] Swanson C. J. Zhang Y. Dhadda S. Wang J. Kaplow J. Lai R. Y. K. (2021). A randomized, double-blind, phase 2b proof-of-concept clinical trial i n early Alzheimer's disease with lecanemab, an anti-Aβ protofibril ant ibody. Alzheimers Res. Ther. 13, 80. 10.1186/s13195-021-00813-8 33865446 PMC8053280

[B29] van Dyck C. H. Swanson C. J. Aisen P. Bateman R. J. Chen C. Gee M. (2023). Lecanemab in early Alzheimer's disease. N. Engl. J. Med. 388, 9–21. 10.1056/NEJMoa2212948 36449413

[B30] Xi Y. Bao Z. Guo Q. Wang J. Jing Z. Di J. (2024). Reproductive toxicity induced by serotonin-norepinephrine reuptake inh ibitors: a pharmacovigilance analysis from 2004 to 2023 based on the F aers database. CNS Neurosci. Ther. 30, e70176. 10.1111/cns.70176 39670536 PMC11638886

[B31] Yamazaki A. Sekine T. Takahashi S. Sohara K. Sakamaki M. Nagao T. (2025). A case of severe ARIA with multiple infarctions and extensive microble eds following lecanemab administration. Psychogeriatrics 25, e13231. 10.1111/psyg.13231 39694473 PMC11655173

[B32] Zhao J. Tao Y. (2024). Adverse event reporting of the IGF-1R monoclonal antibody teprotumumab: a real-world study based on the US food and drug administration adve rse event reporting system. Front. Pharmacol. 15, 1393940. 10.3389/fphar.2024.1393940 39185318 PMC11341477

