# Supplementary material for: A real-world disproportionality analysis of FDA adverse event reporting system (FAERS) events for lecanemab
Source: Front Pharmacol. 2025 Apr 2;16:1559447. doi: 10.3389/fphar.2025.1559447 (PMC12000022; doi:10.3389/fphar.2025.1559447)
Supplement: Supplementary file 1 [file Supplementaryfile1.docx]

Supplementary Material

# Supplementary Data

Supplementary Material should be uploaded separately on submission. Please include any supplementary data, figures and/or tables.

Supplementary material is not typeset so please ensure that all information is clearly presented, the appropriate caption is included in the file and not in the manuscript, and that the style conforms to the rest of the article.

# Supplementary Figures and Tables

## 2.1 Supplementary Figures

##
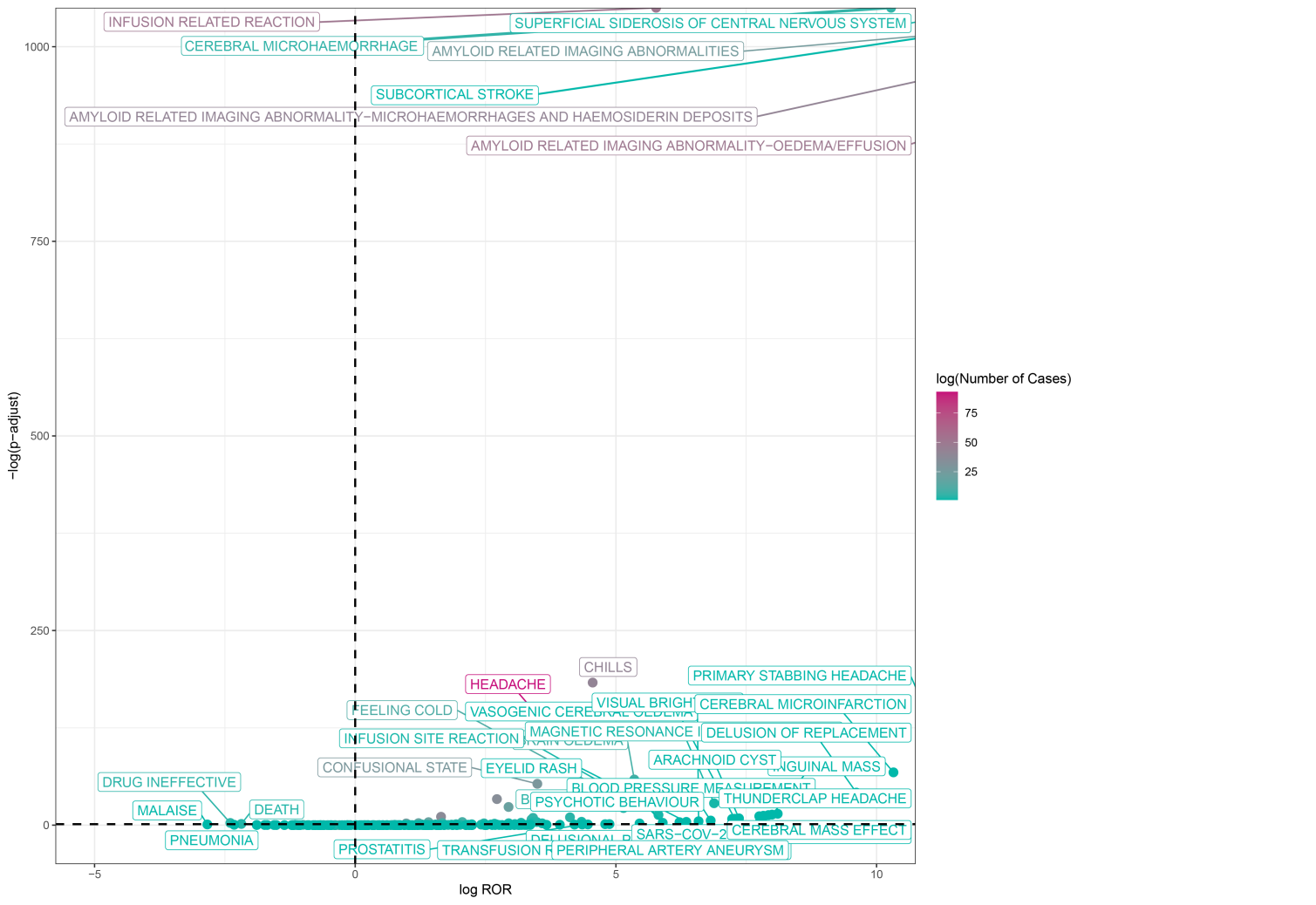


## **Supplementary Figure 1.** The volcano picture of the PT in lecanemab used for Non-AD patients.


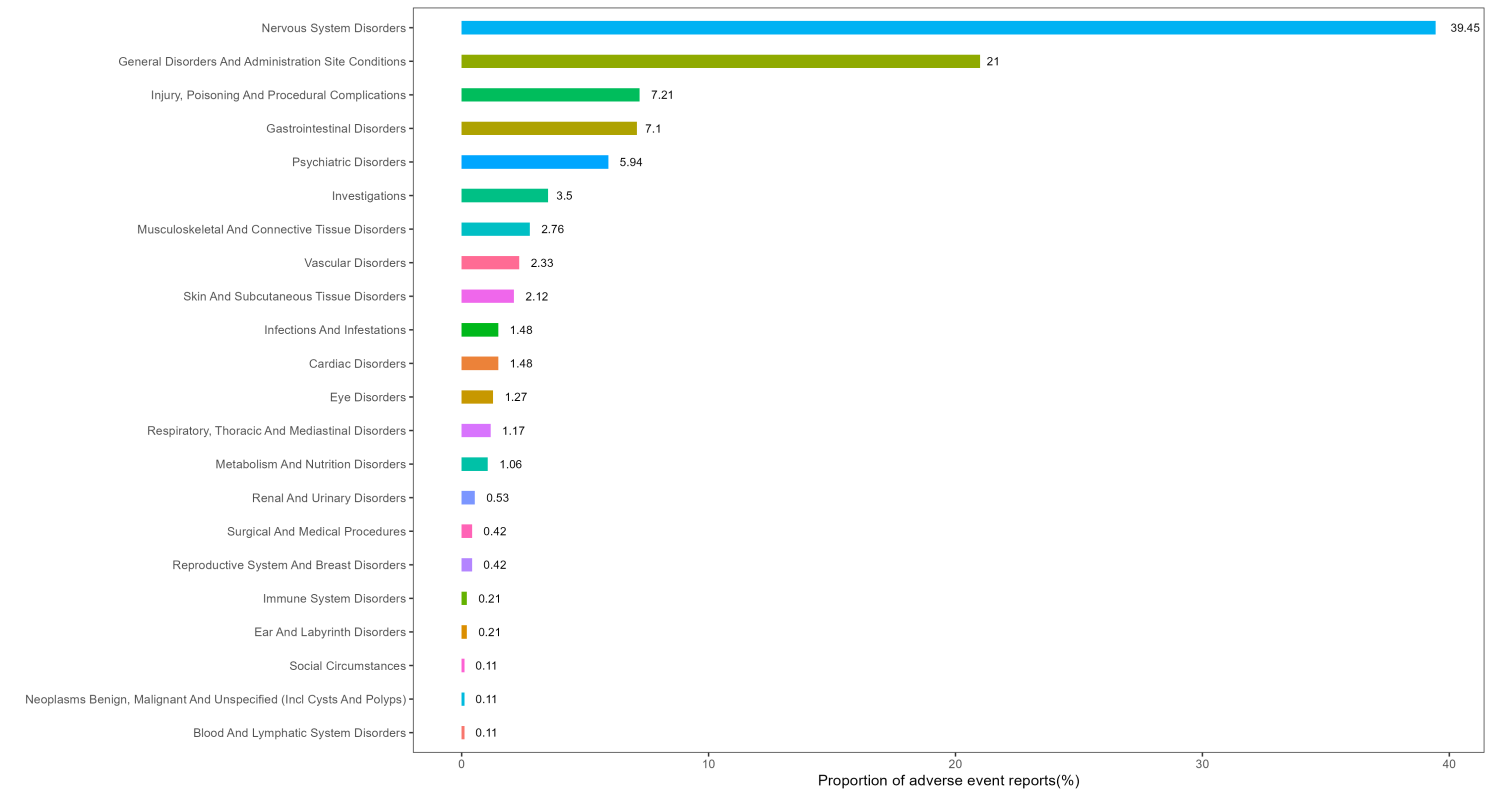
**Supplementary Figure 2.** The percentage of the AE at SOC level in lecanemab used for Non-AD patients.


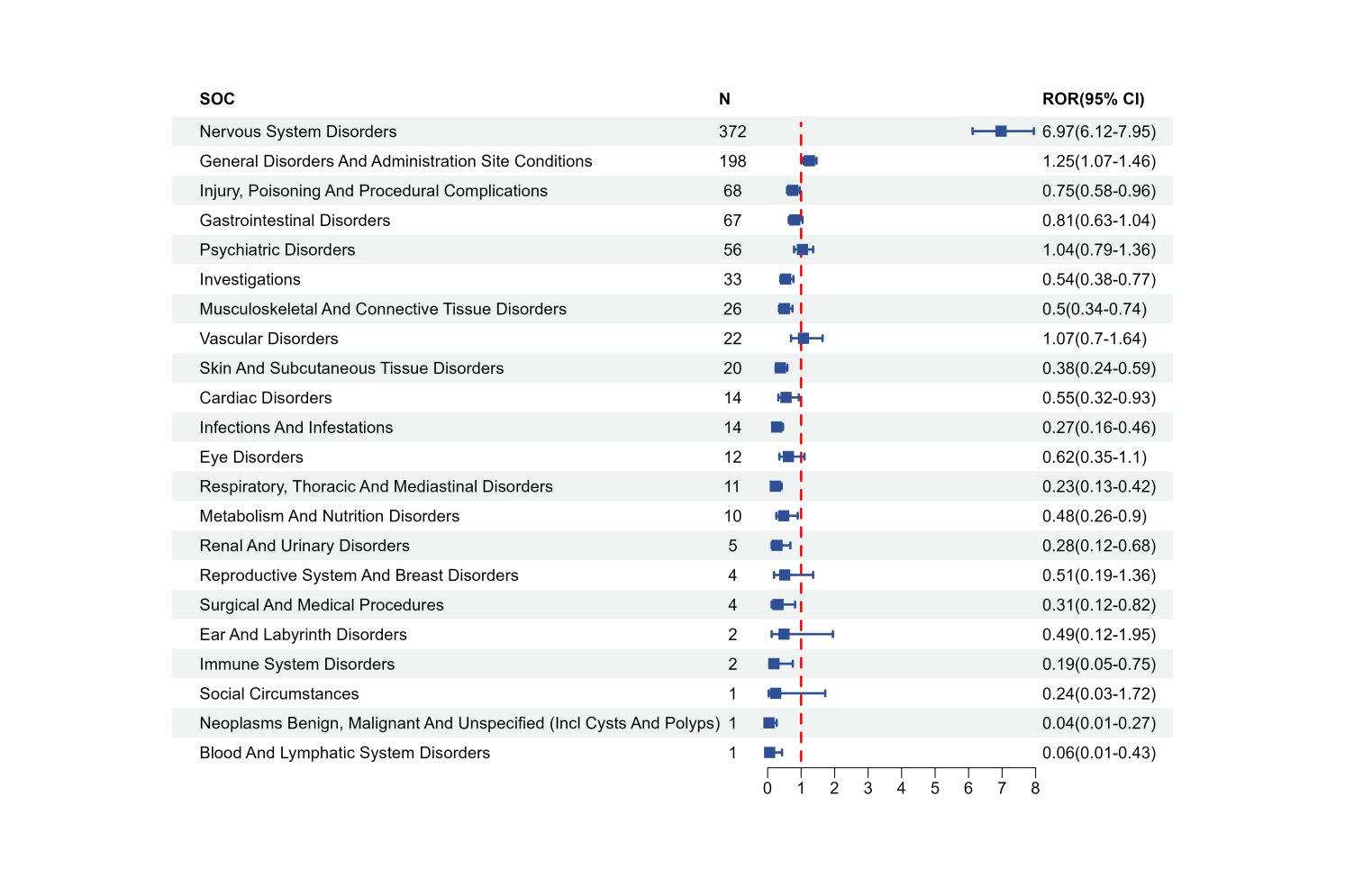


**Supplementary Figure 3.** The forest picture of the SOC level in lecanemab used for Non-AD patients.


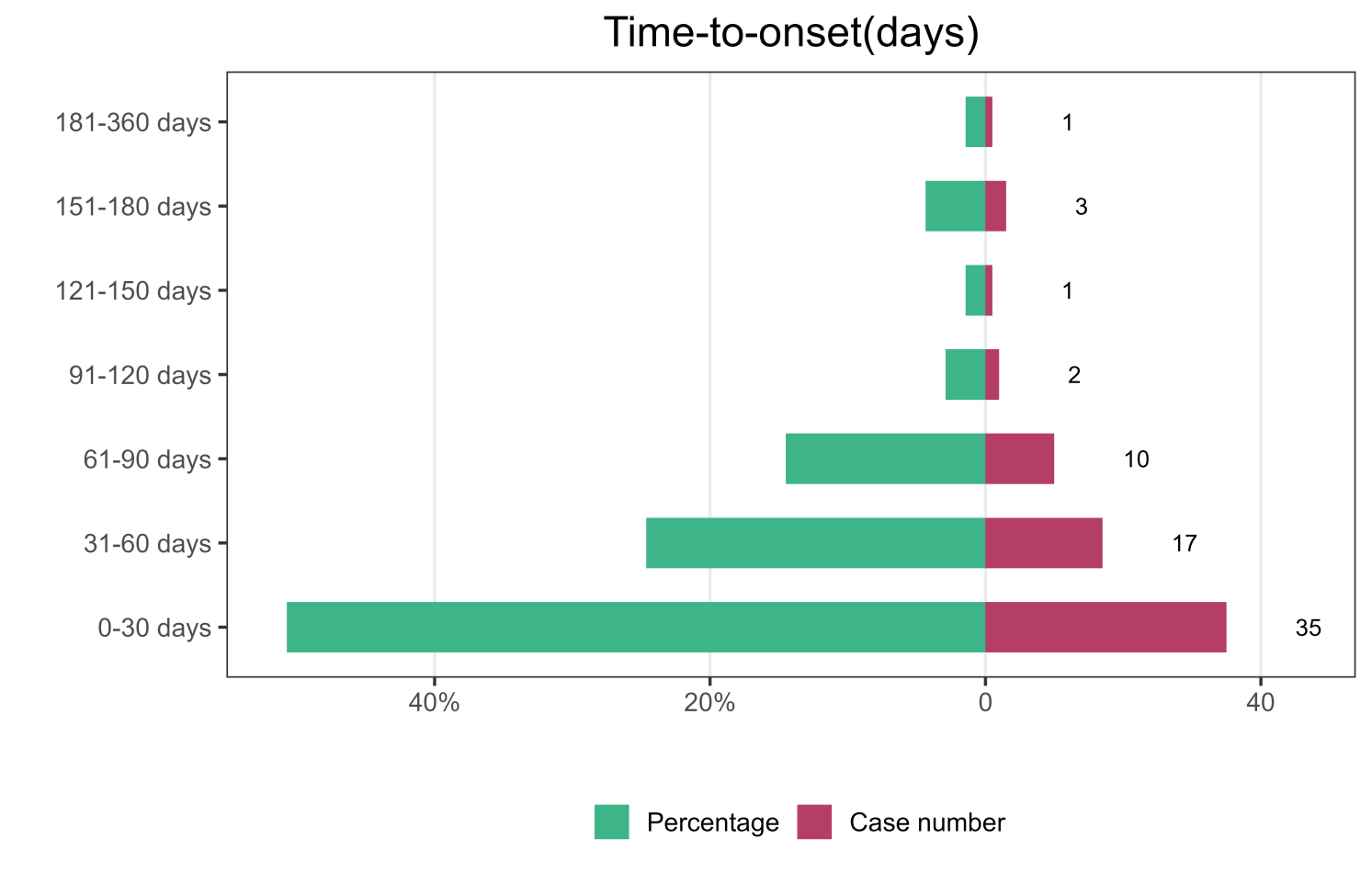


**Supplementary Figure 4.** The time of AE onset in lecanemab used for Non-AD patients.

## 2.2 Supplementary Table

**Supplementary Table 1.** Clinical characteristics of lecanemab in Non-AD

| Characteristics | Lecanemab (N, %) |
| --- | --- |
| Total Number of Reports | 506 |
| Gender |  |
| Female | 240 (47.4%) |
| Male | 173 (34.2%) |
| Unknow | 93 (18.4%) |
| Weight |  |
| <50Kg | 7 (1.4%) |
| 50-100Kg | 44 (8.7%) |
| >100Kg | 4 (0.8%) |
| Unknow | 451 (89.1%) |
| Age |  |
| <65 | 35 (6.9%) |
| 65-85 | 256 (50.6%) |
| >85 | 8 (1.6%) |
| Unknow | 207 (40.9%) |
| Report Person |  |
| Consumer (CN) | 194 (38.3%) |
| Health Professional (HP) | 119 (23.5%) |
| Physician (MD) | 169 (33.4%) |
| Pharmacist (PH) | 15 (3%) |
| Unknow | 9 (1.8%) |
| Serious outcome |  |
| Death (DE) | 7 (1.4%) |
| Hospitalization (HO) | 50 (9.9%) |
| Life-threatening (LT) | 1 (0.2%) |
| Other Serious Outcomes (OT) | 27 (5.3%) |
| Unknow | 421 (83.2%) |
| Report Countries |  |
| United States | 479 (94.7%) |
| Japan | 26 (5.1%) |
| Israel | 1 (0.2%) |

**Supplementary Table 2.** PTs of lecanemab among Non-AD identified as positive across all four algorithms.

| PT | N | RR(95%CI) | ROR(95%Cl) | X^2^ | IC(IC025) | EBGM(EBGM05) |
| --- | --- | --- | --- | --- | --- | --- |
| Headache | 93 | 9.4 (9.21 - 9.59) | 10.32 ( 8.33 - 12.78 ) | 705.22 | 3.23 ( 2.92 ) | 9.4 ( 7.86 ) |
| Infusion Related Reaction | 51 | 51.72 (51.46 - 51.99) | 54.62 ( 41.19 - 72.44 ) | 2537.27 | 5.69 ( 5.28 ) | 51.68 ( 40.81 ) |
| **ARIA-E** | 50 | 313052.49 (313051.78 - 313053.19) | 330580.56 ( 162085.56 - 674233.47 ) | 2387675.85 | 15.54 ( 14.99 ) | 47754.62 ( 26303.79 ) |
| Chills | 42 | 22.5 (22.21 - 22.8) | 23.5 ( 17.25 - 32.03 ) | 864.29 | 4.49 ( 4.04 ) | 22.49 ( 17.36 ) |
| **ARIA-H** | 42 | 147917.3 (147916.73 - 147917.87) | 154812.4 ( 86721.8 - 276365.11 ) | 1713779.14 | 15.32 ( 14.73 ) | 40805.5 ( 25126.47 ) |
| Fatigue | 37 | 3.05 (2.73 - 3.36) | 3.13 ( 2.25 - 4.35 ) | 51.52 | 1.61 ( 1.13 ) | 3.05 ( 2.31 ) |
| Pyrexia | 35 | 6.37 (6.04 - 6.69) | 6.58 ( 4.69 - 9.22 ) | 159.33 | 2.67 ( 2.18 ) | 6.37 ( 4.8 ) |
| Confusional State | 28 | 10.96 (10.59 - 11.32) | 11.26 ( 7.73 - 16.41 ) | 254.07 | 3.45 ( 2.91 ) | 10.96 ( 8 ) |
| Amyloid Related Imaging Abnormalities | 25 | 140873.62 (140872.89 - 140874.35) | 144710.02 ( 69305.16 - 302156.28 ) | 1006226.51 | 15.3 ( 14.55 ) | 40250.32 ( 21738.9 ) |
| Tremor | 20 | 7.54 (7.11 - 7.98) | 7.68 ( 4.93 - 11.97 ) | 113.79 | 2.91 ( 2.28 ) | 7.54 ( 5.2 ) |
| Influenza Like Illness | 15 | 11.22 (10.71 - 11.72) | 11.38 ( 6.83 - 18.96 ) | 139.76 | 3.49 ( 2.76 ) | 11.21 ( 7.32 ) |
| Feeling Cold | 11 | 24.84 (24.25 - 25.43) | 25.12 ( 13.86 - 45.52 ) | 251.67 | 4.63 ( 3.8 ) | 24.83 ( 15.1 ) |
| Memory Impairment | 9 | 4.12 (3.47 - 4.77) | 4.15 ( 2.15 - 8.01 ) | 21.34 | 2.04 ( 1.13 ) | 4.12 ( 2.38 ) |
| **Brain Oedema** | 8 | 40.52 (39.83 - 41.21) | 40.86 ( 20.37 - 81.97 ) | 308.21 | 5.34 ( 4.37 ) | 40.49 ( 22.61 ) |
| **Cerebral Haemorrhage** | 6 | 10.58 (9.78 - 11.38) | 10.64 ( 4.77 - 23.75 ) | 52.07 | 3.4 ( 2.31 ) | 10.58 ( 5.4 ) |
| Aphasia | 5 | 10.37 (9.49 - 11.24) | 10.42 ( 4.33 - 25.09 ) | 42.34 | 3.37 ( 2.19 ) | 10.37 ( 4.97 ) |
| **Cerebral Microhaemorrhage** | 5 | 1235.73 (1234.85 - 1236.62) | 1242.32 ( 510.98 - 3020.38 ) | 6036.32 | 10.24 ( 9.04 ) | 1209.24 ( 575.01 ) |
| Cognitive Disorder | 4 | 5.52 (4.55 - 6.5) | 5.54 ( 2.08 - 14.8 ) | 14.83 | 2.47 ( 1.17 ) | 5.52 ( 2.43 ) |
| Brain Fog | 4 | 35.25 (34.27 - 36.23) | 35.4 ( 13.25 - 94.54 ) | 133.05 | 5.14 ( 3.84 ) | 35.23 ( 15.49 ) |
| **Subdural Haematoma** | 4 | 17.31 (16.33 - 18.28) | 17.38 ( 6.51 - 46.4 ) | 61.45 | 4.11 ( 2.82 ) | 17.3 ( 7.61 ) |
| Body Temperature Increased | 3 | 9.04 (7.91 - 10.17) | 9.07 ( 2.92 - 28.17 ) | 21.46 | 3.18 ( 1.73 ) | 9.04 ( 3.5 ) |
| Gait Inability | 3 | 6.35 (5.22 - 7.48) | 6.37 ( 2.05 - 19.79 ) | 13.54 | 2.67 ( 1.22 ) | 6.35 ( 2.46 ) |
| Head Discomfort | 3 | 11.15 (10.02 - 12.28) | 11.18 ( 3.6 - 34.73 ) | 27.71 | 3.48 ( 2.03 ) | 11.14 ( 4.32 ) |
| **Ischaemic Stroke** | 3 | 10.55 (9.42 - 11.68) | 10.58 ( 3.41 - 32.87 ) | 25.94 | 3.4 ( 1.95 ) | 10.55 ( 4.09 ) |
| **Cerebral Infarction** | 3 | 7.67 (6.54 - 8.8) | 7.69 ( 2.48 - 23.9 ) | 17.41 | 2.94 ( 1.49 ) | 7.67 ( 2.97 ) |

**Supplementary Table 3.** All PTs of lecanemab among AD.

| PT | N | RR(95% CI) | ROR(95%Cl) | X2 | EBGM(EBGM05) | IC(IC025) |
| --- | --- | --- | --- | --- | --- | --- |
| Headache | 170 | 14.79 (14.61 - 14.97) | 16.07 ( 13.32 - 19.4 ) | 1500.82 | 10.39 ( 8.88 ) | 3.38 ( 3.12 ) |
| ARIA-E | 99 | 21.27 (21.02 - 21.52) | 22.33 ( 17.23 - 28.92 ) | 1146.94 | 13.11 ( 10.56 ) | 3.71 ( 3.37 ) |
| Chills | 96 | 53.18 (52.86 - 53.51) | 55.83 ( 40.1 - 77.72 ) | 1835.61 | 20.44 ( 15.5 ) | 4.35 ( 3.97 ) |
| ARIA-H | 90 | 27.07 (26.79 - 27.35) | 28.3 ( 21.28 - 37.64 ) | 1220.26 | 15.04 ( 11.84 ) | 3.91 ( 3.54 ) |
| Fatigue | 89 | 7.22 (7 - 7.45) | 7.52 ( 5.94 - 9.5 ) | 391.34 | 6.07 ( 4.98 ) | 2.6 ( 2.26 ) |
| Infusion Related Reaction | 65 | 293.23 (292.45 - 294.01) | 303.08 ( 138.8 - 661.82 ) | 1842.47 | 29.41 ( 15.3 ) | 4.88 ( 4.38 ) |
| Nausea | 60 | 2.78 (2.52 - 3.04) | 2.84 ( 2.17 - 3.71 ) | 63.68 | 2.64 ( 2.11 ) | 1.4 ( 1.01 ) |
| Confusional State | 59 | 1.68 (1.42 - 1.94) | 1.7 ( 1.31 - 2.22 ) | 15.76 | 1.65 ( 1.32 ) | 0.72 ( 0.33 ) |
| Dizziness | 55 | 2.07 (1.8 - 2.34) | 2.1 ( 1.59 - 2.77 ) | 29.05 | 2.01 ( 1.59 ) | 1 ( 0.6 ) |
| Pyrexia | 50 | 4.52 (4.23 - 4.82) | 4.61 ( 3.42 - 6.23 ) | 120.8 | 4.08 ( 3.18 ) | 2.03 ( 1.6 ) |
| Vomiting | 33 | 1.19 (0.85 - 1.54) | 1.2 ( 0.84 - 1.7 ) | 1 | 1.19 ( 0.88 ) | 0.25 ( -0.26 ) |
| Tremor | 31 | 2.3 (1.94 - 2.66) | 2.32 ( 1.61 - 3.35 ) | 21.34 | 2.21 ( 1.63 ) | 1.14 ( 0.61 ) |
| Pain | 28 | 4.89 (4.49 - 5.28) | 4.94 ( 3.31 - 7.37 ) | 75.17 | 4.36 ( 3.12 ) | 2.13 ( 1.55 ) |
| Diarrhoea | 28 | 1.49 (1.11 - 1.86) | 1.5 ( 1.02 - 2.19 ) | 4.33 | 1.47 ( 1.07 ) | 0.55 ( 0 ) |
| Asthenia | 27 | 1.53 (1.14 - 1.91) | 1.53 ( 1.04 - 2.26 ) | 4.7 | 1.5 ( 1.08 ) | 0.59 ( 0.02 ) |
| Fall | 22 | 0.53 (0.11 - 0.95) | 0.52 ( 0.34 - 0.8 ) | 9.37 | 0.53 ( 0.38 ) | -0.9 ( -1.51 ) |
| Influenza Like Illness | 21 | 73.68 (72.9 - 74.46) | 74.46 ( 34.06 - 162.78 ) | 451.9 | 22.8 ( 11.85 ) | 4.51 ( 3.7 ) |
| Somnolence | 19 | 0.91 (0.46 - 1.37) | 0.91 ( 0.58 - 1.44 ) | 0.15 | 0.92 ( 0.62 ) | -0.13 ( -0.78 ) |
| Gait Disturbance | 19 | 1.29 (0.83 - 1.74) | 1.29 ( 0.81 - 2.05 ) | 1.18 | 1.28 ( 0.87 ) | 0.35 ( -0.31 ) |
| Amyloid Related Imaging Abnormalities | 18 | 43.72 (43.01 - 44.44) | 44.11 ( 21.58 - 90.16 ) | 315.27 | 18.92 ( 10.4 ) | 4.24 ( 3.4 ) |
| Feeling Cold | 15 | 22.56 (21.89 - 23.22) | 22.72 ( 11.69 - 44.14 ) | 180.35 | 13.57 ( 7.79 ) | 3.76 ( 2.9 ) |
| Decreased Appetite | 15 | 0.82 (0.31 - 1.33) | 0.82 ( 0.49 - 1.37 ) | 0.57 | 0.83 ( 0.54 ) | -0.27 ( -1.01 ) |
| Dementia Alzheimer'S Type | 14 | 0.56 (0.03 - 1.08) | 0.55 ( 0.33 - 0.94 ) | 4.89 | 0.57 ( 0.36 ) | -0.82 ( -1.58 ) |
| Syncope | 14 | 0.87 (0.34 - 1.4) | 0.87 ( 0.51 - 1.48 ) | 0.27 | 0.87 ( 0.56 ) | -0.19 ( -0.95 ) |
| Seizure | 14 | 2.47 (1.93 - 3.01) | 2.48 ( 1.44 - 4.28 ) | 11.39 | 2.36 ( 1.5 ) | 1.24 ( 0.47 ) |
| Cerebral Haemorrhage | 13 | 2.55 (1.99 - 3.11) | 2.56 ( 1.45 - 4.51 ) | 11.36 | 2.43 ( 1.51 ) | 1.28 ( 0.48 ) |
| Nasopharyngitis | 13 | 6.32 (5.72 - 6.91) | 6.35 ( 3.5 - 11.54 ) | 48.53 | 5.43 ( 3.29 ) | 2.44 ( 1.6 ) |
| Memory Impairment | 13 | 1.1 (0.55 - 1.65) | 1.1 ( 0.63 - 1.92 ) | 0.12 | 1.1 ( 0.69 ) | 0.13 ( -0.65 ) |
| Weight Decreased | 13 | 0.87 (0.32 - 1.42) | 0.87 ( 0.5 - 1.51 ) | 0.24 | 0.88 ( 0.55 ) | -0.19 ( -0.98 ) |
| Cerebrovascular Accident | 12 | 1.1 (0.52 - 1.67) | 1.1 ( 0.62 - 1.95 ) | 0.1 | 1.09 ( 0.67 ) | 0.13 ( -0.69 ) |
| Cough | 12 | 2.98 (2.39 - 3.57) | 3 ( 1.65 - 5.42 ) | 14.49 | 2.81 ( 1.71 ) | 1.49 ( 0.65 ) |
| Covid-19 | 12 | 13.07 (12.4 - 13.74) | 13.14 ( 6.7 - 25.79 ) | 94.64 | 9.54 ( 5.42 ) | 3.25 ( 2.34 ) |
| Lethargy | 12 | 2.11 (1.52 - 2.69) | 2.11 ( 1.18 - 3.79 ) | 6.55 | 2.04 ( 1.25 ) | 1.03 ( 0.2 ) |
| Balance Disorder | 12 | 2.11 (1.52 - 2.69) | 2.11 ( 1.18 - 3.79 ) | 6.55 | 2.04 ( 1.25 ) | 1.03 ( 0.2 ) |
| Feeling Abnormal | 10 | 1.2 (0.57 - 1.83) | 1.2 ( 0.64 - 2.26 ) | 0.32 | 1.19 ( 0.7 ) | 0.26 ( -0.63 ) |
| Blood Pressure Increased | 10 | 1.5 (0.86 - 2.13) | 1.5 ( 0.79 - 2.83 ) | 1.58 | 1.47 ( 0.87 ) | 0.56 ( -0.33 ) |
| Back Pain | 10 | 3.59 (2.94 - 4.24) | 3.6 ( 1.87 - 6.94 ) | 16.79 | 3.32 ( 1.92 ) | 1.73 ( 0.81 ) |
| Migraine | 9 | 12.36 (11.59 - 13.13) | 12.41 ( 5.73 - 26.85 ) | 67.55 | 9.16 ( 4.8 ) | 3.2 ( 2.15 ) |
| Brain Oedema | 9 | 11.84 (11.08 - 12.61) | 11.89 ( 5.52 - 25.62 ) | 65.01 | 8.89 ( 4.68 ) | 3.15 ( 2.11 ) |
| Rash | 9 | 1.39 (0.72 - 2.05) | 1.39 ( 0.71 - 2.71 ) | 0.93 | 1.37 ( 0.78 ) | 0.45 ( -0.48 ) |
| Drug Ineffective | 9 | 0.56 (-0.1 - 1.21) | 0.55 ( 0.29 - 1.07 ) | 3.18 | 0.56 ( 0.32 ) | -0.83 ( -1.75 ) |
| Atrial Fibrillation | 8 | 1.28 (0.58 - 1.99) | 1.28 ( 0.63 - 2.61 ) | 0.48 | 1.27 ( 0.7 ) | 0.35 ( -0.64 ) |
| Arthralgia | 8 | 2.48 (1.76 - 3.19) | 2.48 ( 1.21 - 5.11 ) | 6.54 | 2.37 ( 1.3 ) | 1.24 ( 0.24 ) |
| Insomnia | 8 | 0.7 (0 - 1.4) | 0.7 ( 0.35 - 1.42 ) | 0.98 | 0.71 ( 0.39 ) | -0.49 ( -1.47 ) |
| Hypotension | 8 | 0.84 (0.14 - 1.54) | 0.84 ( 0.41 - 1.69 ) | 0.25 | 0.84 ( 0.47 ) | -0.25 ( -1.23 ) |
| Cognitive Disorder | 7 | 0.87 (0.12 - 1.62) | 0.87 ( 0.41 - 1.85 ) | 0.12 | 0.88 ( 0.47 ) | -0.19 ( -1.23 ) |
| Brain Fog | 7 | 44.21 (43.06 - 45.36) | 44.36 ( 14.07 - 139.9 ) | 123.2 | 19 ( 7.27 ) | 4.25 ( 2.95 ) |
| Pruritus | 7 | 1.26 (0.5 - 2.01) | 1.26 ( 0.59 - 2.68 ) | 0.35 | 1.25 ( 0.66 ) | 0.32 ( -0.73 ) |
| Visual Impairment | 7 | 6.91 (6.09 - 7.72) | 6.93 ( 3.05 - 15.72 ) | 29.04 | 5.85 ( 2.95 ) | 2.55 ( 1.43 ) |
| Disorientation | 7 | 0.77 (0.02 - 1.52) | 0.77 ( 0.36 - 1.64 ) | 0.46 | 0.78 ( 0.42 ) | -0.36 ( -1.4 ) |
| Anxiety | 6 | 0.78 (-0.03 - 1.59) | 0.78 ( 0.35 - 1.75 ) | 0.37 | 0.79 ( 0.4 ) | -0.35 ( -1.46 ) |
| Anger | 6 | 2.15 (1.33 - 2.98) | 2.16 ( 0.94 - 4.94 ) | 3.47 | 2.08 ( 1.04 ) | 1.06 ( -0.08 ) |
| Hallucination | 6 | 0.39 (-0.41 - 1.19) | 0.39 ( 0.17 - 0.87 ) | 5.73 | 0.4 ( 0.2 ) | -1.33 ( -2.43 ) |
| Pain In Extremity | 6 | 1.75 (0.93 - 2.58) | 1.76 ( 0.77 - 4 ) | 1.85 | 1.71 ( 0.86 ) | 0.78 ( -0.35 ) |
| Dyspnoea | 6 | 0.51 (-0.29 - 1.32) | 0.51 ( 0.23 - 1.14 ) | 2.78 | 0.52 ( 0.26 ) | -0.95 ( -2.05 ) |
| Heart Rate Decreased | 6 | 1.89 (1.07 - 2.72) | 1.9 ( 0.83 - 4.33 ) | 2.4 | 1.84 ( 0.92 ) | 0.88 ( -0.24 ) |
| Pneumonia | 6 | 0.27 (-0.53 - 1.07) | 0.27 ( 0.12 - 0.59 ) | 12.03 | 0.27 ( 0.14 ) | -1.87 ( -2.96 ) |
| Malaise | 6 | 0.34 (-0.46 - 1.15) | 0.34 ( 0.15 - 0.77 ) | 7.47 | 0.35 ( 0.18 ) | -1.51 ( -2.61 ) |
| Renal Failure | 5 | 0.82 (-0.06 - 1.71) | 0.82 ( 0.34 - 2 ) | 0.19 | 0.83 ( 0.39 ) | -0.27 ( -1.47 ) |
| Urinary Tract Infection | 5 | 0.35 (-0.53 - 1.23) | 0.35 ( 0.14 - 0.84 ) | 6.06 | 0.36 ( 0.17 ) | -1.49 ( -2.68 ) |
| Agitation | 5 | 0.21 (-0.67 - 1.09) | 0.21 ( 0.09 - 0.5 ) | 14.88 | 0.22 ( 0.1 ) | -2.21 ( -3.4 ) |
| Muscular Weakness | 5 | 1.7 (0.8 - 2.6) | 1.7 ( 0.69 - 4.18 ) | 1.36 | 1.66 ( 0.78 ) | 0.73 ( -0.48 ) |
| Haemorrhage | 5 | 2.36 (1.45 - 3.26) | 2.36 ( 0.95 - 5.86 ) | 3.64 | 2.26 ( 1.06 ) | 1.18 ( -0.05 ) |
| Musculoskeletal Stiffness | 5 | 1.66 (0.76 - 2.56) | 1.66 ( 0.68 - 4.09 ) | 1.25 | 1.63 ( 0.77 ) | 0.7 ( -0.51 ) |
| Abnormal Dreams | 5 | 4.05 (3.12 - 4.98) | 4.06 ( 1.6 - 10.3 ) | 10.18 | 3.7 ( 1.7 ) | 1.89 ( 0.63 ) |
| Hyperhidrosis | 5 | 1.05 (0.16 - 1.94) | 1.05 ( 0.43 - 2.57 ) | 0.01 | 1.05 ( 0.5 ) | 0.07 ( -1.13 ) |
| Superficial Siderosis Of Central Nervous System | 5 | 7.18 (6.21 - 8.15) | 7.19 ( 2.72 - 19.01 ) | 21.67 | 6.03 ( 2.67 ) | 2.59 ( 1.29 ) |
| Flushing | 5 | 7.18 (6.21 - 8.15) | 7.19 ( 2.72 - 19.01 ) | 21.67 | 6.03 ( 2.67 ) | 2.59 ( 1.29 ) |
| Urinary Incontinence | 5 | 0.81 (-0.08 - 1.7) | 0.81 ( 0.33 - 1.97 ) | 0.22 | 0.81 ( 0.39 ) | -0.3 ( -1.49 ) |
| Pancreatic Carcinoma | 4 | 5.05 (4 - 6.11) | 5.06 ( 1.76 - 14.56 ) | 11.21 | 4.49 ( 1.86 ) | 2.17 ( 0.77 ) |
| Productive Cough | 4 | 3.95 (2.91 - 4.99) | 3.95 ( 1.4 - 11.19 ) | 7.83 | 3.62 ( 1.52 ) | 1.86 ( 0.48 ) |
| Depressed Mood | 4 | 2.11 (1.09 - 3.12) | 2.11 ( 0.77 - 5.8 ) | 2.18 | 2.04 ( 0.87 ) | 1.03 ( -0.31 ) |
| Contusion | 4 | 1.78 (0.77 - 2.79) | 1.78 ( 0.65 - 4.88 ) | 1.29 | 1.74 ( 0.75 ) | 0.8 ( -0.54 ) |
| Hypertension | 4 | 0.49 (-0.5 - 1.48) | 0.49 ( 0.18 - 1.32 ) | 2.08 | 0.5 ( 0.22 ) | -1 ( -2.31 ) |
| Alopecia | 4 | 4.36 (3.31 - 5.4) | 4.36 ( 1.53 - 12.42 ) | 9.09 | 3.95 ( 1.65 ) | 1.98 ( 0.6 ) |
| Abdominal Discomfort | 4 | 1.56 (0.56 - 2.56) | 1.56 ( 0.57 - 4.26 ) | 0.77 | 1.53 ( 0.66 ) | 0.62 ( -0.71 ) |
| Nasal Congestion | 4 | 14.03 (12.86 - 15.21) | 14.06 ( 4.33 - 45.7 ) | 33.53 | 10.02 ( 3.74 ) | 3.33 ( 1.82 ) |
| Chest Pain | 4 | 0.76 (-0.23 - 1.75) | 0.76 ( 0.28 - 2.05 ) | 0.29 | 0.77 ( 0.33 ) | -0.38 ( -1.69 ) |
| Heart Rate Increased | 4 | 2 (1 - 3.01) | 2.01 ( 0.73 - 5.52 ) | 1.9 | 1.94 ( 0.83 ) | 0.96 ( -0.38 ) |
| Abdominal Pain Upper | 4 | 0.97 (-0.02 - 1.97) | 0.97 ( 0.36 - 2.63 ) | 0 | 0.97 ( 0.42 ) | -0.04 ( -1.36 ) |
| Myocardial Infarction | 4 | 0.58 (-0.4 - 1.57) | 0.58 ( 0.22 - 1.57 ) | 1.16 | 0.59 ( 0.26 ) | -0.76 ( -2.06 ) |
| Pallor | 4 | 1.73 (0.72 - 2.74) | 1.73 ( 0.63 - 4.74 ) | 1.17 | 1.69 ( 0.73 ) | 0.76 ( -0.57 ) |
| Head Discomfort | 4 | 5.05 (4 - 6.11) | 5.06 ( 1.76 - 14.56 ) | 11.21 | 4.49 ( 1.86 ) | 2.17 ( 0.77 ) |
| Status Epilepticus | 4 | 5.74 (4.68 - 6.81) | 5.75 ( 1.98 - 16.7 ) | 13.26 | 5.01 ( 2.05 ) | 2.33 ( 0.92 ) |
| Hypersomnia | 4 | 0.96 (-0.04 - 1.95) | 0.96 ( 0.35 - 2.59 ) | 0.01 | 0.96 ( 0.42 ) | -0.06 ( -1.38 ) |
| Sepsis | 4 | 1.07 (0.07 - 2.07) | 1.07 ( 0.39 - 2.9 ) | 0.02 | 1.07 ( 0.46 ) | 0.1 ( -1.22 ) |
| Abdominal Pain | 3 | 0.64 (-0.51 - 1.78) | 0.64 ( 0.2 - 1.99 ) | 0.61 | 0.64 ( 0.25 ) | -0.64 ( -2.1 ) |
| Transient Ischaemic Attack | 3 | 0.99 (-0.16 - 2.14) | 0.99 ( 0.31 - 3.12 ) | 0 | 0.99 ( 0.38 ) | -0.02 ( -1.49 ) |
| Cardiac Failure | 3 | 0.47 (-0.67 - 1.61) | 0.47 ( 0.15 - 1.47 ) | 1.78 | 0.48 ( 0.18 ) | -1.07 ( -2.53 ) |
| Pulmonary Embolism | 3 | 0.78 (-0.36 - 1.93) | 0.78 ( 0.25 - 2.46 ) | 0.18 | 0.79 ( 0.3 ) | -0.34 ( -1.81 ) |
| Generalised Tonic-Clonic Seizure | 3 | 1.89 (0.73 - 3.06) | 1.9 ( 0.59 - 6.08 ) | 1.2 | 1.84 ( 0.7 ) | 0.88 ( -0.61 ) |
| Screaming | 3 | 1.93 (0.77 - 3.1) | 1.93 ( 0.6 - 6.21 ) | 1.27 | 1.88 ( 0.71 ) | 0.91 ( -0.59 ) |
| Peripheral Swelling | 3 | 1.86 (0.69 - 3.02) | 1.86 ( 0.58 - 5.96 ) | 1.12 | 1.81 ( 0.68 ) | 0.86 ( -0.64 ) |
| Sluggishness | 3 | 5.26 (4.04 - 6.48) | 5.27 ( 1.55 - 17.9 ) | 8.88 | 4.65 ( 1.67 ) | 2.22 ( 0.65 ) |
| Dehydration | 3 | 0.25 (-0.88 - 1.39) | 0.25 ( 0.08 - 0.78 ) | 6.65 | 0.26 ( 0.1 ) | -1.96 ( -3.41 ) |
| Poor Quality Sleep | 3 | 4.99 (3.77 - 6.2) | 4.99 ( 1.48 - 16.88 ) | 8.26 | 4.44 ( 1.6 ) | 2.15 ( 0.59 ) |
| Muscle Spasms | 3 | 1.05 (-0.1 - 2.2) | 1.05 ( 0.33 - 3.33 ) | 0.01 | 1.05 ( 0.4 ) | 0.07 ( -1.4 ) |
| Femur Fracture | 3 | 0.62 (-0.52 - 1.77) | 0.62 ( 0.2 - 1.95 ) | 0.67 | 0.63 ( 0.24 ) | -0.67 ( -2.13 ) |
| Feeling Hot | 3 | 3.95 (2.75 - 5.15) | 3.95 ( 1.19 - 13.13 ) | 5.87 | 3.62 ( 1.33 ) | 1.86 ( 0.31 ) |
| Head Injury | 3 | 0.84 (-0.31 - 1.98) | 0.84 ( 0.27 - 2.64 ) | 0.09 | 0.84 ( 0.32 ) | -0.25 ( -1.72 ) |
| Hepatic Enzyme Increased | 3 | 4.12 (2.92 - 5.32) | 4.12 ( 1.24 - 13.75 ) | 6.27 | 3.76 ( 1.37 ) | 1.91 ( 0.36 ) |
| Off Label Use | 3 | 0.18 (-0.95 - 1.32) | 0.18 ( 0.06 - 0.57 ) | 10.82 | 0.19 ( 0.07 ) | -2.4 ( -3.85 ) |
| Blood Glucose Decreased | 3 | 3.38 (2.19 - 4.57) | 3.39 ( 1.03 - 11.15 ) | 4.55 | 3.15 ( 1.16 ) | 1.66 ( 0.13 ) |
| Hiccups | 3 | 4.99 (3.77 - 6.2) | 4.99 ( 1.48 - 16.88 ) | 8.26 | 4.44 ( 1.6 ) | 2.15 ( 0.59 ) |
| Sneezing | 3 | 23.68 (22.19 - 25.18) | 23.72 ( 5.3 - 106.05 ) | 37.25 | 13.96 ( 3.99 ) | 3.8 ( 2.04 ) |
| Incontinence | 3 | 1.3 (0.14 - 2.45) | 1.3 ( 0.41 - 4.12 ) | 0.2 | 1.29 ( 0.49 ) | 0.36 ( -1.12 ) |
| Vertigo | 3 | 1.37 (0.22 - 2.53) | 1.37 ( 0.43 - 4.37 ) | 0.29 | 1.36 ( 0.52 ) | 0.44 ( -1.04 ) |
| Acute Kidney Injury | 3 | 0.88 (-0.27 - 2.02) | 0.88 ( 0.28 - 2.76 ) | 0.05 | 0.88 ( 0.34 ) | -0.18 ( -1.65 ) |
| Myalgia | 3 | 1.86 (0.69 - 3.02) | 1.86 ( 0.58 - 5.96 ) | 1.12 | 1.81 ( 0.68 ) | 0.86 ( -0.64 ) |
| Aphasia | 3 | 0.62 (-0.52 - 1.76) | 0.62 ( 0.2 - 1.94 ) | 0.69 | 0.63 ( 0.24 ) | -0.67 ( -2.14 ) |
| Death | 3 | 0.1 (-1.03 - 1.23) | 0.1 ( 0.03 - 0.3 ) | 25.25 | 0.1 ( 0.04 ) | -3.31 ( -4.76 ) |
| White Blood Cell Count Increased | 3 | 1.28 (0.13 - 2.43) | 1.28 ( 0.4 - 4.07 ) | 0.18 | 1.27 ( 0.48 ) | 0.34 ( -1.14 ) |
| Paraesthesia | 3 | 2.71 (1.53 - 3.88) | 2.71 ( 0.83 - 8.82 ) | 2.98 | 2.57 ( 0.96 ) | 1.36 ( -0.15 ) |
| Body Temperature Increased | 3 | 4.99 (3.77 - 6.2) | 4.99 ( 1.48 - 16.88 ) | 8.26 | 4.44 ( 1.6 ) | 2.15 ( 0.59 ) |
| Amnesia | 3 | 0.76 (-0.39 - 1.9) | 0.76 ( 0.24 - 2.38 ) | 0.23 | 0.76 ( 0.29 ) | -0.39 ( -1.86 ) |
| Therapy Cessation | 3 | 5.57 (4.35 - 6.8) | 5.58 ( 1.63 - 19.05 ) | 9.57 | 4.89 ( 1.75 ) | 2.29 ( 0.71 ) |
| Magnetic Resonance Imaging Abnormal | 3 | 94.74 (92.47 - 97) | 94.88 ( 9.86 - 912.54 ) | 69.56 | 24.43 ( 3.68 ) | 4.61 ( 2.67 ) |
| Mental Status Changes | 2 | 0.9 (-0.5 - 2.31) | 0.9 ( 0.22 - 3.68 ) | 0.02 | 0.9 ( 0.28 ) | -0.14 ( -1.84 ) |
| Cerebral Microhaemorrhage | 2 | 10.53 (8.93 - 12.13) | 10.54 ( 2.13 - 52.23 ) | 12.93 | 8.14 ( 2.13 ) | 3.03 ( 1.1 ) |
| Disturbance In Attention | 2 | 0.9 (-0.5 - 2.31) | 0.9 ( 0.22 - 3.68 ) | 0.02 | 0.9 ( 0.28 ) | -0.14 ( -1.84 ) |
| Palpitations | 2 | 1.34 (-0.07 - 2.76) | 1.34 ( 0.33 - 5.54 ) | 0.17 | 1.33 ( 0.41 ) | 0.41 ( -1.31 ) |
| Crying | 2 | 0.77 (-0.63 - 2.17) | 0.77 ( 0.19 - 3.13 ) | 0.13 | 0.78 ( 0.24 ) | -0.37 ( -2.06 ) |
| Aggression | 2 | 0.11 (-1.28 - 1.49) | 0.1 ( 0.03 - 0.42 ) | 15.24 | 0.11 ( 0.03 ) | -3.2 ( -4.87 ) |
| Hypoacusis | 2 | 3.51 (2.05 - 4.97) | 3.51 ( 0.81 - 15.14 ) | 3.23 | 3.26 ( 0.96 ) | 1.7 ( -0.08 ) |
| Slow Speech | 2 | 10.53 (8.93 - 12.13) | 10.54 ( 2.13 - 52.23 ) | 12.93 | 8.14 ( 2.13 ) | 3.03 ( 1.1 ) |
| Vitreous Floaters | 2 | 31.58 (29.62 - 33.54) | 31.61 ( 4.45 - 224.52 ) | 29.61 | 16.29 ( 3.16 ) | 4.03 ( 1.92 ) |
| Formication | 2 | 31.58 (29.62 - 33.54) | 31.61 ( 4.45 - 224.52 ) | 29.61 | 16.29 ( 3.16 ) | 4.03 ( 1.92 ) |
| Hallucination, Auditory | 2 | 1.24 (-0.17 - 2.65) | 1.24 ( 0.3 - 5.09 ) | 0.09 | 1.23 ( 0.38 ) | 0.3 ( -1.41 ) |
| Hyponatraemia | 2 | 0.42 (-0.97 - 1.82) | 0.42 ( 0.1 - 1.7 ) | 1.57 | 0.43 ( 0.13 ) | -1.22 ( -2.91 ) |
| Interstitial Lung Disease | 2 | 1.4 (-0.01 - 2.82) | 1.4 ( 0.34 - 5.79 ) | 0.22 | 1.39 ( 0.42 ) | 0.47 ( -1.25 ) |
| Skin Abrasion | 2 | 15.79 (14.09 - 17.49) | 15.8 ( 2.89 - 86.34 ) | 18.47 | 10.86 ( 2.62 ) | 3.44 ( 1.45 ) |
| Epilepsy | 2 | 0.52 (-0.87 - 1.92) | 0.52 ( 0.13 - 2.11 ) | 0.86 | 0.53 ( 0.16 ) | -0.92 ( -2.6 ) |
| Swelling | 2 | 2.11 (0.67 - 3.54) | 2.11 ( 0.5 - 8.82 ) | 1.09 | 2.04 ( 0.61 ) | 1.03 ( -0.72 ) |
| Hepatitis B Reactivation | 2 | 0 (0 - 0) | Inf ( NaN - Inf ) | 63.16 | 32.58 ( 0 ) | 5.03 ( 2.67 ) |
| Tenderness | 2 | 31.58 (29.62 - 33.54) | 31.61 ( 4.45 - 224.52 ) | 29.61 | 16.29 ( 3.16 ) | 4.03 ( 1.92 ) |
| Initial Insomnia | 2 | 10.53 (8.93 - 12.13) | 10.54 ( 2.13 - 52.23 ) | 12.93 | 8.14 ( 2.13 ) | 3.03 ( 1.1 ) |
| Magnetic Resonance Imaging Head Abnormal | 2 | 0 (0 - 0) | Inf ( NaN - Inf ) | 63.16 | 32.58 ( 0 ) | 5.03 ( 2.67 ) |
| Product Dose Omission Issue | 2 | 1.86 (0.43 - 3.28) | 1.86 ( 0.45 - 7.74 ) | 0.75 | 1.81 ( 0.55 ) | 0.86 ( -0.88 ) |
| Neovascular Age-Related Macular Degeneration | 2 | 31.58 (29.62 - 33.54) | 31.61 ( 4.45 - 224.52 ) | 29.61 | 16.29 ( 3.16 ) | 4.03 ( 1.92 ) |
| Encephalitis | 2 | 7.89 (6.35 - 9.44) | 7.9 ( 1.68 - 37.23 ) | 9.64 | 6.52 ( 1.78 ) | 2.7 ( 0.82 ) |
| Infusion Site Bruising | 2 | 0 (0 - 0) | Inf ( NaN - Inf ) | 63.16 | 32.58 ( 0 ) | 5.03 ( 2.67 ) |
| Ocular Hyperaemia | 2 | 9.02 (7.45 - 10.59) | 9.03 ( 1.87 - 43.5 ) | 11.1 | 7.24 ( 1.94 ) | 2.86 ( 0.96 ) |
| Poor Venous Access | 2 | 31.58 (29.62 - 33.54) | 31.61 ( 4.45 - 224.52 ) | 29.61 | 16.29 ( 3.16 ) | 4.03 ( 1.92 ) |
| Tinnitus | 2 | 2.63 (1.19 - 4.07) | 2.63 ( 0.62 - 11.15 ) | 1.87 | 2.51 ( 0.75 ) | 1.33 ( -0.43 ) |
| Deep Vein Thrombosis | 2 | 0.97 (-0.43 - 2.38) | 0.97 ( 0.24 - 3.97 ) | 0 | 0.97 ( 0.3 ) | -0.04 ( -1.74 ) |
| Unresponsive To Stimuli | 2 | 0.58 (-0.82 - 1.98) | 0.58 ( 0.14 - 2.35 ) | 0.6 | 0.59 ( 0.18 ) | -0.77 ( -2.46 ) |
| Lacrimation Increased | 2 | 7.89 (6.35 - 9.44) | 7.9 ( 1.68 - 37.23 ) | 9.64 | 6.52 ( 1.78 ) | 2.7 ( 0.82 ) |
| Restlessness | 2 | 0.29 (-1.1 - 1.68) | 0.29 ( 0.07 - 1.17 ) | 3.43 | 0.3 ( 0.09 ) | -1.75 ( -3.43 ) |
| Dizziness Postural | 2 | 3.51 (2.05 - 4.97) | 3.51 ( 0.81 - 15.14 ) | 3.23 | 3.26 ( 0.96 ) | 1.7 ( -0.08 ) |
| Rhinorrhoea | 2 | 2.04 (0.61 - 3.47) | 2.04 ( 0.49 - 8.52 ) | 0.99 | 1.97 ( 0.6 ) | 0.98 ( -0.76 ) |
| Atrioventricular Block | 2 | 0.94 (-0.46 - 2.35) | 0.94 ( 0.23 - 3.85 ) | 0.01 | 0.94 ( 0.29 ) | -0.08 ( -1.78 ) |
| Bradycardia | 2 | 0.1 (-1.28 - 1.49) | 0.1 ( 0.03 - 0.41 ) | 15.52 | 0.11 ( 0.03 ) | -3.22 ( -4.9 ) |
| Cardiac Arrest | 2 | 0.31 (-1.09 - 1.7) | 0.31 ( 0.08 - 1.23 ) | 3.12 | 0.31 ( 0.1 ) | -1.67 ( -3.35 ) |
| Urticaria | 2 | 1.37 (-0.04 - 2.79) | 1.37 ( 0.33 - 5.66 ) | 0.19 | 1.36 ( 0.41 ) | 0.44 ( -1.28 ) |
| Joint Swelling | 2 | 1.47 (0.05 - 2.89) | 1.47 ( 0.36 - 6.07 ) | 0.29 | 1.45 ( 0.44 ) | 0.53 ( -1.19 ) |
| Heart Rate Irregular | 2 | 3.16 (1.7 - 4.61) | 3.16 ( 0.74 - 13.53 ) | 2.68 | 2.96 ( 0.88 ) | 1.57 ( -0.21 ) |
| Rash Pruritic | 2 | 3.51 (2.05 - 4.97) | 3.51 ( 0.81 - 15.14 ) | 3.23 | 3.26 ( 0.96 ) | 1.7 ( -0.08 ) |
| Weight Increased | 2 | 0.63 (-0.77 - 2.02) | 0.62 ( 0.15 - 2.54 ) | 0.44 | 0.63 ( 0.2 ) | -0.66 ( -2.35 ) |
| Chest Discomfort | 2 | 1.71 (0.28 - 3.13) | 1.71 ( 0.41 - 7.09 ) | 0.56 | 1.67 ( 0.51 ) | 0.74 ( -0.99 ) |
| Skin Burning Sensation | 2 | 10.53 (8.93 - 12.13) | 10.54 ( 2.13 - 52.23 ) | 12.93 | 8.14 ( 2.13 ) | 3.03 ( 1.1 ) |
| Illness | 2 | 7.02 (5.49 - 8.55) | 7.02 ( 1.52 - 32.53 ) | 8.44 | 5.92 ( 1.64 ) | 2.57 ( 0.7 ) |
| Eye Swelling | 2 | 7.89 (6.35 - 9.44) | 7.9 ( 1.68 - 37.23 ) | 9.64 | 6.52 ( 1.78 ) | 2.7 ( 0.82 ) |
| Ischaemic Stroke | 2 | 1.47 (0.05 - 2.89) | 1.47 ( 0.36 - 6.07 ) | 0.29 | 1.45 ( 0.44 ) | 0.53 ( -1.19 ) |
| Herpes Zoster | 2 | 2.11 (0.67 - 3.54) | 2.11 ( 0.5 - 8.82 ) | 1.09 | 2.04 ( 0.61 ) | 1.03 ( -0.72 ) |
| Dementia | 2 | 0.27 (-1.12 - 1.66) | 0.27 ( 0.07 - 1.07 ) | 4.02 | 0.27 ( 0.09 ) | -1.88 ( -3.55 ) |
| Road Traffic Accident | 2 | 4.51 (3.03 - 5.99) | 4.51 ( 1.03 - 19.88 ) | 4.78 | 4.07 ( 1.18 ) | 2.03 ( 0.22 ) |
| Retching | 2 | 2.11 (0.67 - 3.54) | 2.11 ( 0.5 - 8.82 ) | 1.09 | 2.04 ( 0.61 ) | 1.03 ( -0.72 ) |
| Vision Blurred | 2 | 1.54 (0.12 - 2.96) | 1.54 ( 0.37 - 6.38 ) | 0.36 | 1.52 ( 0.46 ) | 0.6 ( -1.12 ) |
| Viral Infection | 2 | 3.01 (1.56 - 4.46) | 3.01 ( 0.71 - 12.84 ) | 2.45 | 2.83 ( 0.84 ) | 1.5 ( -0.27 ) |
| Nightmare | 2 | 0.74 (-0.66 - 2.14) | 0.74 ( 0.18 - 3.02 ) | 0.17 | 0.75 ( 0.23 ) | -0.42 ( -2.11 ) |
| Blepharospasm | 2 | 31.58 (29.62 - 33.54) | 31.61 ( 4.45 - 224.52 ) | 29.61 | 16.29 ( 3.16 ) | 4.03 ( 1.92 ) |
| Neck Pain | 2 | 4.51 (3.03 - 5.99) | 4.51 ( 1.03 - 19.88 ) | 4.78 | 4.07 ( 1.18 ) | 2.03 ( 0.22 ) |
| C-Reactive Protein Increased | 2 | 1.11 (-0.3 - 2.52) | 1.11 ( 0.27 - 4.54 ) | 0.02 | 1.1 ( 0.34 ) | 0.14 ( -1.56 ) |
| Encephalopathy | 2 | 1.4 (-0.01 - 2.82) | 1.4 ( 0.34 - 5.79 ) | 0.22 | 1.39 ( 0.42 ) | 0.47 ( -1.25 ) |
| Effusion | 2 | 15.79 (14.09 - 17.49) | 15.8 ( 2.89 - 86.34 ) | 18.47 | 10.86 ( 2.62 ) | 3.44 ( 1.45 ) |
| Cerebral Mass Effect | 2 | 0 (0 - 0) | Inf ( NaN - Inf ) | 63.16 | 32.58 ( 0 ) | 5.03 ( 2.67 ) |
| Thrombocytopenia | 2 | 1.37 (-0.04 - 2.79) | 1.37 ( 0.33 - 5.66 ) | 0.19 | 1.36 ( 0.41 ) | 0.44 ( -1.28 ) |
| Diverticulitis | 2 | 1.91 (0.49 - 3.34) | 1.91 ( 0.46 - 7.99 ) | 0.82 | 1.86 ( 0.56 ) | 0.9 ( -0.84 ) |
| Thirst | 2 | 7.02 (5.49 - 8.55) | 7.02 ( 1.52 - 32.53 ) | 8.44 | 5.92 ( 1.64 ) | 2.57 ( 0.7 ) |
| Loss Of Consciousness | 2 | 0.13 (-1.26 - 1.51) | 0.13 ( 0.03 - 0.5 ) | 12.1 | 0.13 ( 0.04 ) | -2.94 ( -4.61 ) |
| Oedema | 2 | 1.5 (0.09 - 2.92) | 1.5 ( 0.36 - 6.22 ) | 0.32 | 1.48 ( 0.45 ) | 0.57 ( -1.16 ) |
| Frustration Tolerance Decreased | 2 | 31.58 (29.62 - 33.54) | 31.61 ( 4.45 - 224.52 ) | 29.61 | 16.29 ( 3.16 ) | 4.03 ( 1.92 ) |
| Gait Inability | 2 | 1.86 (0.43 - 3.28) | 1.86 ( 0.45 - 7.74 ) | 0.75 | 1.81 ( 0.55 ) | 0.86 ( -0.88 ) |
| Infusion Site Extravasation | 2 | 31.58 (29.62 - 33.54) | 31.61 ( 4.45 - 224.52 ) | 29.61 | 16.29 ( 3.16 ) | 4.03 ( 1.92 ) |
| Cerebral Infarction | 2 | 0.51 (-0.89 - 1.91) | 0.51 ( 0.13 - 2.06 ) | 0.93 | 0.52 ( 0.16 ) | -0.95 ( -2.64 ) |
| Therapy Interrupted | 2 | 9.02 (7.45 - 10.59) | 9.03 ( 1.87 - 43.5 ) | 11.1 | 7.24 ( 1.94 ) | 2.86 ( 0.96 ) |
| Personality Change | 2 | 3.51 (2.05 - 4.97) | 3.51 ( 0.81 - 15.14 ) | 3.23 | 3.26 ( 0.96 ) | 1.7 ( -0.08 ) |
| Cervical Vertebral Fracture | 1 | 3.95 (1.87 - 6.03) | 3.95 ( 0.49 - 31.59 ) | 1.96 | 3.62 ( 0.64 ) | 1.86 ( -0.38 ) |
| Acute Myocardial Infarction | 1 | 0.69 (-1.29 - 2.67) | 0.69 ( 0.09 - 4.98 ) | 0.14 | 0.69 ( 0.13 ) | -0.53 ( -2.61 ) |
| Renal Cyst | 1 | 3.16 (1.1 - 5.21) | 3.16 ( 0.4 - 24.69 ) | 1.34 | 2.96 ( 0.53 ) | 1.57 ( -0.64 ) |
| Ovarian Cancer Metastatic | 1 | 0 (0 - 0) | Inf ( NaN - Inf ) | 31.58 | 32.58 ( 0 ) | 5.03 ( 2.14 ) |
| Small Intestinal Obstruction | 1 | 4.51 (2.42 - 6.61) | 4.51 ( 0.55 - 36.7 ) | 2.39 | 4.07 ( 0.71 ) | 2.03 ( -0.23 ) |
| Condition Aggravated | 1 | 0.06 (-1.9 - 2.02) | 0.06 ( 0.01 - 0.43 ) | 14.68 | 0.06 ( 0.01 ) | -4 ( -6.05 ) |
| Colon Cancer Metastatic | 1 | 0 (0 - 0) | Inf ( NaN - Inf ) | 31.58 | 32.58 ( 0 ) | 5.03 ( 2.14 ) |
| Urinary Retention | 1 | 0.45 (-1.52 - 2.42) | 0.45 ( 0.06 - 3.25 ) | 0.66 | 0.46 ( 0.09 ) | -1.12 ( -3.19 ) |
| Hypertransaminasaemia | 1 | 0 (0 - 0) | Inf ( NaN - Inf ) | 31.58 | 32.58 ( 0 ) | 5.03 ( 2.14 ) |
| Verbal Abuse | 1 | 4.51 (2.42 - 6.61) | 4.51 ( 0.55 - 36.7 ) | 2.39 | 4.07 ( 0.71 ) | 2.03 ( -0.23 ) |
| Tubulointerstitial Nephritis | 1 | 3.16 (1.1 - 5.21) | 3.16 ( 0.4 - 24.69 ) | 1.34 | 2.96 ( 0.53 ) | 1.57 ( -0.64 ) |
| Wheezing | 1 | 1.26 (-0.74 - 3.26) | 1.26 ( 0.17 - 9.33 ) | 0.05 | 1.25 ( 0.24 ) | 0.33 ( -1.79 ) |
| Jaundice | 1 | 0.64 (-1.33 - 2.62) | 0.64 ( 0.09 - 4.67 ) | 0.19 | 0.65 ( 0.12 ) | -0.62 ( -2.7 ) |
| Feeling Jittery | 1 | 2.26 (0.23 - 4.28) | 2.26 ( 0.3 - 17.17 ) | 0.65 | 2.17 ( 0.4 ) | 1.12 ( -1.05 ) |
| Paranasal Sinus Discomfort | 1 | 0 (0 - 0) | Inf ( NaN - Inf ) | 31.58 | 32.58 ( 0 ) | 5.03 ( 2.14 ) |
| Delirium | 1 | 0.09 (-1.87 - 2.05) | 0.09 ( 0.01 - 0.64 ) | 9.17 | 0.09 ( 0.02 ) | -3.43 ( -5.47 ) |
| Hypoaesthesia | 1 | 0.63 (-1.35 - 2.61) | 0.63 ( 0.09 - 4.57 ) | 0.21 | 0.64 ( 0.12 ) | -0.65 ( -2.73 ) |
| Claustrophobia | 1 | 0 (0 - 0) | Inf ( NaN - Inf ) | 31.58 | 32.58 ( 0 ) | 5.03 ( 2.14 ) |
| Emotional Disorder | 1 | 1.5 (-0.5 - 3.51) | 1.5 ( 0.2 - 11.19 ) | 0.16 | 1.48 ( 0.28 ) | 0.57 ( -1.56 ) |
| Intracranial Pressure Increased | 1 | 15.79 (13.39 - 18.19) | 15.8 ( 1.43 - 174.29 ) | 9.24 | 10.86 ( 1.46 ) | 3.44 ( 0.94 ) |
| Dyskinesia | 1 | 0.22 (-1.75 - 2.19) | 0.22 ( 0.03 - 1.58 ) | 2.74 | 0.23 ( 0.04 ) | -2.14 ( -4.2 ) |
| Night Sweats | 1 | 2.87 (0.82 - 4.92) | 2.87 ( 0.37 - 22.25 ) | 1.12 | 2.71 ( 0.49 ) | 1.44 ( -0.75 ) |
| Ocular Discomfort | 1 | 10.53 (8.26 - 12.79) | 10.53 ( 1.09 - 101.29 ) | 6.47 | 8.14 ( 1.23 ) | 3.03 ( 0.61 ) |
| Eye Pruritus | 1 | 6.32 (4.17 - 8.46) | 6.32 ( 0.74 - 54.11 ) | 3.73 | 5.43 ( 0.9 ) | 2.44 ( 0.13 ) |
| Cerebrovascular Disorder | 1 | 3.51 (1.44 - 5.57) | 3.51 ( 0.44 - 27.72 ) | 1.61 | 3.26 ( 0.58 ) | 1.7 ( -0.51 ) |
| Low Density Lipoprotein Increased | 1 | 31.58 (28.81 - 34.35) | 31.59 ( 1.98 - 505.31 ) | 14.81 | 16.29 ( 1.6 ) | 4.03 ( 1.39 ) |
| Bladder Pain | 1 | 0 (0 - 0) | Inf ( NaN - Inf ) | 31.58 | 32.58 ( 0 ) | 5.03 ( 2.14 ) |
| Superficial Vein Thrombosis | 1 | 0 (0 - 0) | Inf ( NaN - Inf ) | 31.58 | 32.58 ( 0 ) | 5.03 ( 2.14 ) |
| Circulatory Collapse | 1 | 0.37 (-1.6 - 2.34) | 0.37 ( 0.05 - 2.64 ) | 1.08 | 0.37 ( 0.07 ) | -1.42 ( -3.48 ) |
| Eye Disorder | 1 | 1.05 (-0.94 - 3.04) | 1.05 ( 0.14 - 7.72 ) | 0 | 1.05 ( 0.2 ) | 0.07 ( -2.03 ) |
| Ear Infection | 1 | 31.58 (28.81 - 34.35) | 31.59 ( 1.98 - 505.31 ) | 14.81 | 16.29 ( 1.6 ) | 4.03 ( 1.39 ) |
| Rhinitis | 1 | 10.53 (8.26 - 12.79) | 10.53 ( 1.09 - 101.29 ) | 6.47 | 8.14 ( 1.23 ) | 3.03 ( 0.61 ) |
| Cerebrosclerosis | 1 | 0 (0 - 0) | Inf ( NaN - Inf ) | 31.58 | 32.58 ( 0 ) | 5.03 ( 2.14 ) |
| Central Nervous System Lesion | 1 | 15.79 (13.39 - 18.19) | 15.8 ( 1.43 - 174.29 ) | 9.24 | 10.86 ( 1.46 ) | 3.44 ( 0.94 ) |
| Ventricular Fibrillation | 1 | 1.86 (-0.16 - 3.87) | 1.86 ( 0.25 - 13.97 ) | 0.37 | 1.81 ( 0.33 ) | 0.86 ( -1.29 ) |
| Electrocardiogram St Segment Elevation | 1 | 6.32 (4.17 - 8.46) | 6.32 ( 0.74 - 54.11 ) | 3.73 | 5.43 ( 0.9 ) | 2.44 ( 0.13 ) |
| Cardiac Disorder | 1 | 0.26 (-1.71 - 2.23) | 0.26 ( 0.04 - 1.85 ) | 2.11 | 0.26 ( 0.05 ) | -1.92 ( -3.97 ) |
| Face Injury | 1 | 4.51 (2.42 - 6.61) | 4.51 ( 0.55 - 36.7 ) | 2.39 | 4.07 ( 0.71 ) | 2.03 ( -0.23 ) |
| Ear Discomfort | 1 | 15.79 (13.39 - 18.19) | 15.8 ( 1.43 - 174.29 ) | 9.24 | 10.86 ( 1.46 ) | 3.44 ( 0.94 ) |
| Delusional Perception | 1 | 0 (0 - 0) | Inf ( NaN - Inf ) | 31.58 | 32.58 ( 0 ) | 5.03 ( 2.14 ) |
| Rash Papular | 1 | 5.26 (3.15 - 7.38) | 5.27 ( 0.63 - 43.76 ) | 2.96 | 4.65 ( 0.79 ) | 2.22 ( -0.06 ) |
| Bradyphrenia | 1 | 3.95 (1.87 - 6.03) | 3.95 ( 0.49 - 31.59 ) | 1.96 | 3.62 ( 0.64 ) | 1.86 ( -0.38 ) |
| Abdominal Pain Lower | 1 | 10.53 (8.26 - 12.79) | 10.53 ( 1.09 - 101.29 ) | 6.47 | 8.14 ( 1.23 ) | 3.03 ( 0.61 ) |
| Sinus Pain | 1 | 0 (0 - 0) | Inf ( NaN - Inf ) | 31.58 | 32.58 ( 0 ) | 5.03 ( 2.14 ) |
| Weight Fluctuation | 1 | 6.32 (4.17 - 8.46) | 6.32 ( 0.74 - 54.11 ) | 3.73 | 5.43 ( 0.9 ) | 2.44 ( 0.13 ) |
| Peripheral Coldness | 1 | 1.97 (-0.05 - 3.99) | 1.97 ( 0.26 - 14.89 ) | 0.45 | 1.92 ( 0.35 ) | 0.94 ( -1.21 ) |
| Dermatitis Acneiform | 1 | 31.58 (28.81 - 34.35) | 31.59 ( 1.98 - 505.31 ) | 14.81 | 16.29 ( 1.6 ) | 4.03 ( 1.39 ) |
| Asthma | 1 | 0.77 (-1.21 - 2.75) | 0.77 ( 0.11 - 5.6 ) | 0.07 | 0.78 ( 0.15 ) | -0.37 ( -2.45 ) |
| Constipation | 1 | 0.13 (-1.83 - 2.09) | 0.13 ( 0.02 - 0.92 ) | 5.82 | 0.13 ( 0.03 ) | -2.9 ( -4.95 ) |
| Pollakiuria | 1 | 0.44 (-1.53 - 2.41) | 0.44 ( 0.06 - 3.16 ) | 0.71 | 0.45 ( 0.09 ) | -1.16 ( -3.23 ) |
| Tachyphrenia | 1 | 0 (0 - 0) | Inf ( NaN - Inf ) | 31.58 | 32.58 ( 0 ) | 5.03 ( 2.14 ) |
| Swelling Face | 1 | 2.11 (0.08 - 4.13) | 2.11 ( 0.28 - 15.95 ) | 0.54 | 2.04 ( 0.37 ) | 1.03 ( -1.13 ) |
| Depression | 1 | 0.13 (-1.84 - 2.09) | 0.13 ( 0.02 - 0.91 ) | 5.95 | 0.13 ( 0.03 ) | -2.93 ( -4.98 ) |
| Dry Mouth | 1 | 0.93 (-1.06 - 2.92) | 0.93 ( 0.13 - 6.79 ) | 0.01 | 0.93 ( 0.18 ) | -0.1 ( -2.2 ) |
| Conjunctival Haemorrhage | 1 | 15.79 (13.39 - 18.19) | 15.8 ( 1.43 - 174.29 ) | 9.24 | 10.86 ( 1.46 ) | 3.44 ( 0.94 ) |
| Gastroenteritis Viral | 1 | 7.89 (5.7 - 10.09) | 7.9 ( 0.88 - 70.7 ) | 4.82 | 6.52 ( 1.04 ) | 2.7 ( 0.35 ) |
| Bronchitis | 1 | 0.73 (-1.25 - 2.72) | 0.73 ( 0.1 - 5.33 ) | 0.09 | 0.74 ( 0.14 ) | -0.43 ( -2.52 ) |
| Cerebral Thrombosis | 1 | 2.63 (0.59 - 4.67) | 2.63 ( 0.34 - 20.26 ) | 0.93 | 2.51 ( 0.45 ) | 1.33 ( -0.86 ) |
| Tearfulness | 1 | 3.16 (1.1 - 5.21) | 3.16 ( 0.4 - 24.69 ) | 1.34 | 2.96 ( 0.53 ) | 1.57 ( -0.64 ) |
| Rib Fracture | 1 | 0.96 (-1.03 - 2.95) | 0.96 ( 0.13 - 7 ) | 0 | 0.96 ( 0.18 ) | -0.06 ( -2.16 ) |
| Clumsiness | 1 | 7.89 (5.7 - 10.09) | 7.9 ( 0.88 - 70.7 ) | 4.82 | 6.52 ( 1.04 ) | 2.7 ( 0.35 ) |
| Depressive Symptom | 1 | 15.79 (13.39 - 18.19) | 15.8 ( 1.43 - 174.29 ) | 9.24 | 10.86 ( 1.46 ) | 3.44 ( 0.94 ) |
| Traumatic Intracranial Haemorrhage | 1 | 10.53 (8.26 - 12.79) | 10.53 ( 1.09 - 101.29 ) | 6.47 | 8.14 ( 1.23 ) | 3.03 ( 0.61 ) |
| Metabolic Encephalopathy | 1 | 2.43 (0.4 - 4.46) | 2.43 ( 0.32 - 18.58 ) | 0.78 | 2.33 ( 0.42 ) | 1.22 ( -0.95 ) |
| Disorganised Speech | 1 | 15.79 (13.39 - 18.19) | 15.8 ( 1.43 - 174.29 ) | 9.24 | 10.86 ( 1.46 ) | 3.44 ( 0.94 ) |
| Burning Sensation | 1 | 1.75 (-0.26 - 3.77) | 1.75 ( 0.23 - 13.15 ) | 0.31 | 1.71 ( 0.32 ) | 0.78 ( -1.36 ) |
| Paraesthesia Oral | 1 | 31.58 (28.81 - 34.35) | 31.59 ( 1.98 - 505.31 ) | 14.81 | 16.29 ( 1.6 ) | 4.03 ( 1.39 ) |
| Eye Allergy | 1 | 0 (0 - 0) | Inf ( NaN - Inf ) | 31.58 | 32.58 ( 0 ) | 5.03 ( 2.14 ) |
| Tooth Infection | 1 | 6.32 (4.17 - 8.46) | 6.32 ( 0.74 - 54.11 ) | 3.73 | 5.43 ( 0.9 ) | 2.44 ( 0.13 ) |
| Infusion Site Pain | 1 | 0 (0 - 0) | Inf ( NaN - Inf ) | 31.58 | 32.58 ( 0 ) | 5.03 ( 2.14 ) |
| Tachycardia | 1 | 0.47 (-1.5 - 2.45) | 0.47 ( 0.07 - 3.39 ) | 0.58 | 0.48 ( 0.09 ) | -1.06 ( -3.13 ) |
| Dysarthria | 1 | 0.41 (-1.56 - 2.38) | 0.41 ( 0.06 - 2.95 ) | 0.84 | 0.42 ( 0.08 ) | -1.26 ( -3.33 ) |
| Rosacea | 1 | 10.53 (8.26 - 12.79) | 10.53 ( 1.09 - 101.29 ) | 6.47 | 8.14 ( 1.23 ) | 3.03 ( 0.61 ) |
| Rash Erythematous | 1 | 1.37 (-0.63 - 3.37) | 1.37 ( 0.19 - 10.17 ) | 0.1 | 1.36 ( 0.25 ) | 0.44 ( -1.68 ) |
| Axillary Pain | 1 | 31.58 (28.81 - 34.35) | 31.59 ( 1.98 - 505.31 ) | 14.81 | 16.29 ( 1.6 ) | 4.03 ( 1.39 ) |
| Dysphagia | 1 | 0.1 (-1.87 - 2.06) | 0.1 ( 0.01 - 0.68 ) | 8.5 | 0.1 ( 0.02 ) | -3.34 ( -5.38 ) |
| Rash Macular | 1 | 1.97 (-0.05 - 3.99) | 1.97 ( 0.26 - 14.89 ) | 0.45 | 1.92 ( 0.35 ) | 0.94 ( -1.21 ) |
| Calculus Bladder | 1 | 15.79 (13.39 - 18.19) | 15.8 ( 1.43 - 174.29 ) | 9.24 | 10.86 ( 1.46 ) | 3.44 ( 0.94 ) |
| Increased Appetite | 1 | 2.63 (0.59 - 4.67) | 2.63 ( 0.34 - 20.26 ) | 0.93 | 2.51 ( 0.45 ) | 1.33 ( -0.86 ) |
| Sleep Terror | 1 | 3.95 (1.87 - 6.03) | 3.95 ( 0.49 - 31.59 ) | 1.96 | 3.62 ( 0.64 ) | 1.86 ( -0.38 ) |
| Muscle Twitching | 1 | 0.72 (-1.26 - 2.7) | 0.72 ( 0.1 - 5.21 ) | 0.11 | 0.72 ( 0.14 ) | -0.47 ( -2.55 ) |
| Flatulence | 1 | 1.05 (-0.94 - 3.04) | 1.05 ( 0.14 - 7.72 ) | 0 | 1.05 ( 0.2 ) | 0.07 ( -2.03 ) |
| Bundle Branch Block | 1 | 5.26 (3.15 - 7.38) | 5.27 ( 0.63 - 43.76 ) | 2.96 | 4.65 ( 0.79 ) | 2.22 ( -0.06 ) |
| Dyspraxia | 1 | 31.58 (28.81 - 34.35) | 31.59 ( 1.98 - 505.31 ) | 14.81 | 16.29 ( 1.6 ) | 4.03 ( 1.39 ) |
| Coccidioidomycosis | 1 | 0 (0 - 0) | Inf ( NaN - Inf ) | 31.58 | 32.58 ( 0 ) | 5.03 ( 2.14 ) |
| Hypothermia | 1 | 0.57 (-1.4 - 2.55) | 0.57 ( 0.08 - 4.15 ) | 0.31 | 0.58 ( 0.11 ) | -0.78 ( -2.86 ) |
| Subarachnoid Haemorrhage | 1 | 0.81 (-1.17 - 2.79) | 0.81 ( 0.11 - 5.9 ) | 0.04 | 0.81 ( 0.15 ) | -0.3 ( -2.39 ) |
| Withdrawal Syndrome | 1 | 0.83 (-1.15 - 2.82) | 0.83 ( 0.11 - 6.06 ) | 0.03 | 0.84 ( 0.16 ) | -0.26 ( -2.35 ) |
| Cholecystitis | 1 | 2.63 (0.59 - 4.67) | 2.63 ( 0.34 - 20.26 ) | 0.93 | 2.51 ( 0.45 ) | 1.33 ( -0.86 ) |
| Femoral Neck Fracture | 1 | 0.41 (-1.56 - 2.38) | 0.41 ( 0.06 - 2.95 ) | 0.84 | 0.42 ( 0.08 ) | -1.26 ( -3.33 ) |
| Altered State Of Consciousness | 1 | 0.18 (-1.79 - 2.14) | 0.18 ( 0.03 - 1.28 ) | 3.74 | 0.18 ( 0.04 ) | -2.44 ( -4.49 ) |
| Blood Brain Barrier Defect | 1 | 0 (0 - 0) | Inf ( NaN - Inf ) | 31.58 | 32.58 ( 0 ) | 5.03 ( 2.14 ) |
| Device Use Issue | 1 | 10.53 (8.26 - 12.79) | 10.53 ( 1.09 - 101.29 ) | 6.47 | 8.14 ( 1.23 ) | 3.03 ( 0.61 ) |
| Cerebral Sulcal Prominence | 1 | 0 (0 - 0) | Inf ( NaN - Inf ) | 31.58 | 32.58 ( 0 ) | 5.03 ( 2.14 ) |
| Pelvic Fracture | 1 | 1.44 (-0.57 - 3.44) | 1.44 ( 0.19 - 10.66 ) | 0.13 | 1.42 ( 0.26 ) | 0.5 ( -1.62 ) |
| Facial Pain | 1 | 15.79 (13.39 - 18.19) | 15.8 ( 1.43 - 174.29 ) | 9.24 | 10.86 ( 1.46 ) | 3.44 ( 0.94 ) |
| Cold Sweat | 1 | 0.62 (-1.36 - 2.6) | 0.62 ( 0.09 - 4.48 ) | 0.23 | 0.63 ( 0.12 ) | -0.67 ( -2.75 ) |
| Groin Pain | 1 | 10.53 (8.26 - 12.79) | 10.53 ( 1.09 - 101.29 ) | 6.47 | 8.14 ( 1.23 ) | 3.03 ( 0.61 ) |
| Anaemia | 1 | 0.15 (-1.82 - 2.11) | 0.15 ( 0.02 - 1.05 ) | 4.89 | 0.15 ( 0.03 ) | -2.72 ( -4.77 ) |
| Gastrooesophageal Reflux Disease | 1 | 0.9 (-1.09 - 2.89) | 0.9 ( 0.12 - 6.59 ) | 0.01 | 0.9 ( 0.17 ) | -0.14 ( -2.24 ) |
| Gingival Swelling | 1 | 0 (0 - 0) | Inf ( NaN - Inf ) | 31.58 | 32.58 ( 0 ) | 5.03 ( 2.14 ) |
| Gingival Pain | 1 | 15.79 (13.39 - 18.19) | 15.8 ( 1.43 - 174.29 ) | 9.24 | 10.86 ( 1.46 ) | 3.44 ( 0.94 ) |
| Panic Attack | 1 | 0.73 (-1.25 - 2.72) | 0.73 ( 0.1 - 5.33 ) | 0.09 | 0.74 ( 0.14 ) | -0.43 ( -2.52 ) |
| Petechiae | 1 | 2.26 (0.23 - 4.28) | 2.26 ( 0.3 - 17.17 ) | 0.65 | 2.17 ( 0.4 ) | 1.12 ( -1.05 ) |
| Physical Deconditioning | 1 | 7.89 (5.7 - 10.09) | 7.9 ( 0.88 - 70.7 ) | 4.82 | 6.52 ( 1.04 ) | 2.7 ( 0.35 ) |
| Colitis | 1 | 2.26 (0.23 - 4.28) | 2.26 ( 0.3 - 17.17 ) | 0.65 | 2.17 ( 0.4 ) | 1.12 ( -1.05 ) |
| Nervous System Disorder | 1 | 0.96 (-1.03 - 2.95) | 0.96 ( 0.13 - 7 ) | 0 | 0.96 ( 0.18 ) | -0.06 ( -2.16 ) |
| Muscle Tightness | 1 | 6.32 (4.17 - 8.46) | 6.32 ( 0.74 - 54.11 ) | 3.73 | 5.43 ( 0.9 ) | 2.44 ( 0.13 ) |
| Arthropod Bite | 1 | 10.53 (8.26 - 12.79) | 10.53 ( 1.09 - 101.29 ) | 6.47 | 8.14 ( 1.23 ) | 3.03 ( 0.61 ) |
| Pain In Jaw | 1 | 15.79 (13.39 - 18.19) | 15.8 ( 1.43 - 174.29 ) | 9.24 | 10.86 ( 1.46 ) | 3.44 ( 0.94 ) |
| Ageusia | 1 | 4.51 (2.42 - 6.61) | 4.51 ( 0.55 - 36.7 ) | 2.39 | 4.07 ( 0.71 ) | 2.03 ( -0.23 ) |
| Photophobia | 1 | 10.53 (8.26 - 12.79) | 10.53 ( 1.09 - 101.29 ) | 6.47 | 8.14 ( 1.23 ) | 3.03 ( 0.61 ) |
| Haemorrhagic Stroke | 1 | 0.81 (-1.17 - 2.79) | 0.81 ( 0.11 - 5.9 ) | 0.04 | 0.81 ( 0.15 ) | -0.3 ( -2.39 ) |
| Taste Disorder | 1 | 0 (0 - 0) | Inf ( NaN - Inf ) | 31.58 | 32.58 ( 0 ) | 5.03 ( 2.14 ) |
| Cerebral Haematoma | 1 | 1.17 (-0.83 - 3.17) | 1.17 ( 0.16 - 8.61 ) | 0.02 | 1.16 ( 0.22 ) | 0.22 ( -1.89 ) |
| Negative Thoughts | 1 | 15.79 (13.39 - 18.19) | 15.8 ( 1.43 - 174.29 ) | 9.24 | 10.86 ( 1.46 ) | 3.44 ( 0.94 ) |
| Blood Pressure Decreased | 1 | 0.16 (-1.8 - 2.12) | 0.16 ( 0.02 - 1.14 ) | 4.42 | 0.16 ( 0.03 ) | -2.61 ( -4.66 ) |
| Limb Discomfort | 1 | 3.51 (1.44 - 5.57) | 3.51 ( 0.44 - 27.72 ) | 1.61 | 3.26 ( 0.58 ) | 1.7 ( -0.51 ) |
| Cerebellar Stroke | 1 | 0 (0 - 0) | Inf ( NaN - Inf ) | 31.58 | 32.58 ( 0 ) | 5.03 ( 2.14 ) |
| Intraventricular Haemorrhage | 1 | 15.79 (13.39 - 18.19) | 15.8 ( 1.43 - 174.29 ) | 9.24 | 10.86 ( 1.46 ) | 3.44 ( 0.94 ) |
| General Physical Health Deterioration | 1 | 0.11 (-1.85 - 2.07) | 0.11 ( 0.02 - 0.77 ) | 7.3 | 0.11 ( 0.02 ) | -3.16 ( -5.21 ) |
| Tension Headache | 1 | 10.53 (8.26 - 12.79) | 10.53 ( 1.09 - 101.29 ) | 6.47 | 8.14 ( 1.23 ) | 3.03 ( 0.61 ) |
| Liver Function Test Increased | 1 | 2.87 (0.82 - 4.92) | 2.87 ( 0.37 - 22.25 ) | 1.12 | 2.71 ( 0.49 ) | 1.44 ( -0.75 ) |
| Fibrin D Dimer Increased | 1 | 4.51 (2.42 - 6.61) | 4.51 ( 0.55 - 36.7 ) | 2.39 | 4.07 ( 0.71 ) | 2.03 ( -0.23 ) |
| Injection Site Erythema | 1 | 0 (0 - 0) | Inf ( NaN - Inf ) | 31.58 | 32.58 ( 0 ) | 5.03 ( 2.14 ) |
| Injection Site Rash | 1 | 0 (0 - 0) | Inf ( NaN - Inf ) | 31.58 | 32.58 ( 0 ) | 5.03 ( 2.14 ) |
| Blood Glucose Increased | 1 | 0.21 (-1.76 - 2.17) | 0.21 ( 0.03 - 1.48 ) | 3.01 | 0.21 ( 0.04 ) | -2.23 ( -4.29 ) |
| Sinus Headache | 1 | 0 (0 - 0) | Inf ( NaN - Inf ) | 31.58 | 32.58 ( 0 ) | 5.03 ( 2.14 ) |
| Oral Pain | 1 | 7.89 (5.7 - 10.09) | 7.9 ( 0.88 - 70.7 ) | 4.82 | 6.52 ( 1.04 ) | 2.7 ( 0.35 ) |
| Oral Mucosal Exfoliation | 1 | 15.79 (13.39 - 18.19) | 15.8 ( 1.43 - 174.29 ) | 9.24 | 10.86 ( 1.46 ) | 3.44 ( 0.94 ) |
| Parosmia | 1 | 31.58 (28.81 - 34.35) | 31.59 ( 1.98 - 505.31 ) | 14.81 | 16.29 ( 1.6 ) | 4.03 ( 1.39 ) |
| Musculoskeletal Discomfort | 1 | 6.32 (4.17 - 8.46) | 6.32 ( 0.74 - 54.11 ) | 3.73 | 5.43 ( 0.9 ) | 2.44 ( 0.13 ) |
| Upper Gastrointestinal Haemorrhage | 1 | 0.88 (-1.11 - 2.86) | 0.88 ( 0.12 - 6.4 ) | 0.02 | 0.88 ( 0.17 ) | -0.18 ( -2.28 ) |
| Dialysis | 1 | 4.51 (2.42 - 6.61) | 4.51 ( 0.55 - 36.7 ) | 2.39 | 4.07 ( 0.71 ) | 2.03 ( -0.23 ) |
| Hyperkalaemia | 1 | 1.02 (-0.97 - 3.01) | 1.02 ( 0.14 - 7.47 ) | 0 | 1.02 ( 0.19 ) | 0.03 ( -2.08 ) |
| Ear Haemorrhage | 1 | 10.53 (8.26 - 12.79) | 10.53 ( 1.09 - 101.29 ) | 6.47 | 8.14 ( 1.23 ) | 3.03 ( 0.61 ) |
| Oedema Peripheral | 1 | 0.2 (-1.76 - 2.17) | 0.2 ( 0.03 - 1.45 ) | 3.1 | 0.21 ( 0.04 ) | -2.26 ( -4.31 ) |
| Ileus | 1 | 1.44 (-0.57 - 3.44) | 1.44 ( 0.19 - 10.66 ) | 0.13 | 1.42 ( 0.26 ) | 0.5 ( -1.62 ) |
| Brain Natriuretic Peptide Increased | 1 | 4.51 (2.42 - 6.61) | 4.51 ( 0.55 - 36.7 ) | 2.39 | 4.07 ( 0.71 ) | 2.03 ( -0.23 ) |
| Gastric Dilatation | 1 | 0 (0 - 0) | Inf ( NaN - Inf ) | 31.58 | 32.58 ( 0 ) | 5.03 ( 2.14 ) |
| Hypokalaemia | 1 | 0.27 (-1.7 - 2.23) | 0.26 ( 0.04 - 1.9 ) | 2.02 | 0.27 ( 0.05 ) | -1.88 ( -3.94 ) |
| Hypoxia | 1 | 1.13 (-0.87 - 3.12) | 1.13 ( 0.15 - 8.29 ) | 0.01 | 1.12 ( 0.21 ) | 0.17 ( -1.94 ) |
| Electrocardiogram Qt Prolonged | 1 | 0.13 (-1.83 - 2.09) | 0.13 ( 0.02 - 0.93 ) | 5.79 | 0.13 ( 0.03 ) | -2.9 ( -4.95 ) |
| Abdominal Rigidity | 1 | 0 (0 - 0) | Inf ( NaN - Inf ) | 31.58 | 32.58 ( 0 ) | 5.03 ( 2.14 ) |
| Gastroenteritis | 1 | 0.93 (-1.06 - 2.92) | 0.93 ( 0.13 - 6.79 ) | 0.01 | 0.93 ( 0.18 ) | -0.1 ( -2.2 ) |
| Feeding Disorder | 1 | 0.55 (-1.42 - 2.53) | 0.55 ( 0.08 - 4 ) | 0.35 | 0.56 ( 0.11 ) | -0.83 ( -2.91 ) |
| Coronavirus Infection | 1 | 10.53 (8.26 - 12.79) | 10.53 ( 1.09 - 101.29 ) | 6.47 | 8.14 ( 1.23 ) | 3.03 ( 0.61 ) |
| Treatment Delayed | 1 | 0 (0 - 0) | Inf ( NaN - Inf ) | 31.58 | 32.58 ( 0 ) | 5.03 ( 2.14 ) |
| Joint Dislocation | 1 | 3.51 (1.44 - 5.57) | 3.51 ( 0.44 - 27.72 ) | 1.61 | 3.26 ( 0.58 ) | 1.7 ( -0.51 ) |
| Aortic Dissection | 1 | 10.53 (8.26 - 12.79) | 10.53 ( 1.09 - 101.29 ) | 6.47 | 8.14 ( 1.23 ) | 3.03 ( 0.61 ) |
| Glioma | 1 | 0 (0 - 0) | Inf ( NaN - Inf ) | 31.58 | 32.58 ( 0 ) | 5.03 ( 2.14 ) |
| Endocarditis | 1 | 0 (0 - 0) | Inf ( NaN - Inf ) | 31.58 | 32.58 ( 0 ) | 5.03 ( 2.14 ) |
| Sinusitis | 1 | 3.51 (1.44 - 5.57) | 3.51 ( 0.44 - 27.72 ) | 1.61 | 3.26 ( 0.58 ) | 1.7 ( -0.51 ) |
| Loss Of Personal Independence In Daily Activities | 1 | 1.75 (-0.26 - 3.77) | 1.75 ( 0.23 - 13.15 ) | 0.31 | 1.71 ( 0.32 ) | 0.78 ( -1.36 ) |
| Aspartate Aminotransferase Increased | 1 | 0.38 (-1.59 - 2.35) | 0.38 ( 0.05 - 2.73 ) | 1 | 0.39 ( 0.07 ) | -1.37 ( -3.43 ) |
| Alanine Aminotransferase Increased | 1 | 0.42 (-1.56 - 2.39) | 0.42 ( 0.06 - 2.99 ) | 0.81 | 0.42 ( 0.08 ) | -1.24 ( -3.31 ) |
| Drug-Induced Liver Injury | 1 | 0.58 (-1.39 - 2.56) | 0.58 ( 0.08 - 4.23 ) | 0.29 | 0.59 ( 0.11 ) | -0.76 ( -2.83 ) |
| Hospice Care | 1 | 6.32 (4.17 - 8.46) | 6.32 ( 0.74 - 54.11 ) | 3.73 | 5.43 ( 0.9 ) | 2.44 ( 0.13 ) |
| Coma | 1 | 0.23 (-1.73 - 2.2) | 0.23 ( 0.03 - 1.66 ) | 2.53 | 0.24 ( 0.05 ) | -2.07 ( -4.13 ) |
| Irritability | 1 | 0.27 (-1.7 - 2.24) | 0.27 ( 0.04 - 1.95 ) | 1.93 | 0.28 ( 0.05 ) | -1.84 ( -3.9 ) |
| Vasogenic Cerebral Oedema | 1 | 15.79 (13.39 - 18.19) | 15.8 ( 1.43 - 174.29 ) | 9.24 | 10.86 ( 1.46 ) | 3.44 ( 0.94 ) |
| Metabolic Acidosis | 1 | 2.26 (0.23 - 4.28) | 2.26 ( 0.3 - 17.17 ) | 0.65 | 2.17 ( 0.4 ) | 1.12 ( -1.05 ) |
| Organ Failure | 1 | 6.32 (4.17 - 8.46) | 6.32 ( 0.74 - 54.11 ) | 3.73 | 5.43 ( 0.9 ) | 2.44 ( 0.13 ) |
| Retinal Oedema | 1 | 15.79 (13.39 - 18.19) | 15.8 ( 1.43 - 174.29 ) | 9.24 | 10.86 ( 1.46 ) | 3.44 ( 0.94 ) |
| Ulcerative Keratitis | 1 | 31.58 (28.81 - 34.35) | 31.59 ( 1.98 - 505.31 ) | 14.81 | 16.29 ( 1.6 ) | 4.03 ( 1.39 ) |
| Hyperpyrexia | 1 | 7.89 (5.7 - 10.09) | 7.9 ( 0.88 - 70.7 ) | 4.82 | 6.52 ( 1.04 ) | 2.7 ( 0.35 ) |
| White Blood Cell Count Decreased | 1 | 0.99 (-1 - 2.98) | 0.99 ( 0.13 - 7.23 ) | 0 | 0.99 ( 0.19 ) | -0.02 ( -2.12 ) |
| Blister | 1 | 0.46 (-1.52 - 2.43) | 0.46 ( 0.06 - 3.3 ) | 0.63 | 0.47 ( 0.09 ) | -1.1 ( -3.17 ) |
| Hunger | 1 | 5.26 (3.15 - 7.38) | 5.27 ( 0.63 - 43.76 ) | 2.96 | 4.65 ( 0.79 ) | 2.22 ( -0.06 ) |
| Blood Pressure Systolic Increased | 1 | 0.73 (-1.25 - 2.72) | 0.73 ( 0.1 - 5.33 ) | 0.09 | 0.74 ( 0.14 ) | -0.43 ( -2.52 ) |
| Epistaxis | 1 | 0.96 (-1.03 - 2.95) | 0.96 ( 0.13 - 7 ) | 0 | 0.96 ( 0.18 ) | -0.06 ( -2.16 ) |
| Prodromal Alzheimer'S Disease | 1 | 0 (0 - 0) | Inf ( NaN - Inf ) | 31.58 | 32.58 ( 0 ) | 5.03 ( 2.14 ) |
| Pulmonary Haemosiderosis | 1 | 0 (0 - 0) | Inf ( NaN - Inf ) | 31.58 | 32.58 ( 0 ) | 5.03 ( 2.14 ) |
| Aortic Aneurysm Rupture | 1 | 31.58 (28.81 - 34.35) | 31.59 ( 1.98 - 505.31 ) | 14.81 | 16.29 ( 1.6 ) | 4.03 ( 1.39 ) |
| Disease Progression | 1 | 0.28 (-1.69 - 2.25) | 0.28 ( 0.04 - 2 ) | 1.84 | 0.29 ( 0.06 ) | -1.81 ( -3.87 ) |
| Cardiomyopathy | 1 | 2.11 (0.08 - 4.13) | 2.11 ( 0.28 - 15.95 ) | 0.54 | 2.04 ( 0.37 ) | 1.03 ( -1.13 ) |
| Haemorrhagic Hepatic Cyst | 1 | 0 (0 - 0) | Inf ( NaN - Inf ) | 31.58 | 32.58 ( 0 ) | 5.03 ( 2.14 ) |
| Infusion Site Erythema | 1 | 0 (0 - 0) | Inf ( NaN - Inf ) | 31.58 | 32.58 ( 0 ) | 5.03 ( 2.14 ) |
| Injection Site Paraesthesia | 1 | 31.58 (28.81 - 34.35) | 31.59 ( 1.98 - 505.31 ) | 14.81 | 16.29 ( 1.6 ) | 4.03 ( 1.39 ) |
| Injection Site Pain | 1 | 4.51 (2.42 - 6.61) | 4.51 ( 0.55 - 36.7 ) | 2.39 | 4.07 ( 0.71 ) | 2.03 ( -0.23 ) |
| Breast Cancer | 1 | 1.09 (-0.9 - 3.08) | 1.09 ( 0.15 - 8 ) | 0.01 | 1.09 ( 0.2 ) | 0.12 ( -1.99 ) |
| Discomfort | 1 | 2.43 (0.4 - 4.46) | 2.43 ( 0.32 - 18.58 ) | 0.78 | 2.33 ( 0.42 ) | 1.22 ( -0.95 ) |
| Partial Seizures | 1 | 1.66 (-0.35 - 3.67) | 1.66 ( 0.22 - 12.42 ) | 0.25 | 1.63 ( 0.3 ) | 0.7 ( -1.43 ) |
| Lacunar Infarction | 1 | 1.37 (-0.63 - 3.37) | 1.37 ( 0.19 - 10.17 ) | 0.1 | 1.36 ( 0.25 ) | 0.44 ( -1.68 ) |
| Dysphonia | 1 | 1.58 (-0.43 - 3.59) | 1.58 ( 0.21 - 11.77 ) | 0.2 | 1.55 ( 0.29 ) | 0.63 ( -1.5 ) |
| Tooth Abscess | 1 | 31.58 (28.81 - 34.35) | 31.59 ( 1.98 - 505.31 ) | 14.81 | 16.29 ( 1.6 ) | 4.03 ( 1.39 ) |
| Infection | 1 | 0.26 (-1.71 - 2.23) | 0.26 ( 0.04 - 1.87 ) | 2.08 | 0.27 ( 0.05 ) | -1.9 ( -3.96 ) |
| Pulmonary Mass | 1 | 3.95 (1.87 - 6.03) | 3.95 ( 0.49 - 31.59 ) | 1.96 | 3.62 ( 0.64 ) | 1.86 ( -0.38 ) |
| Haemoptysis | 1 | 2.87 (0.82 - 4.92) | 2.87 ( 0.37 - 22.25 ) | 1.12 | 2.71 ( 0.49 ) | 1.44 ( -0.75 ) |
| Ophthalmic Vein Thrombosis | 1 | 0 (0 - 0) | Inf ( NaN - Inf ) | 31.58 | 32.58 ( 0 ) | 5.03 ( 2.14 ) |
| Blindness | 1 | 1.5 (-0.5 - 3.51) | 1.5 ( 0.2 - 11.19 ) | 0.16 | 1.48 ( 0.28 ) | 0.57 ( -1.56 ) |
| Speech Disorder | 1 | 0.12 (-1.84 - 2.09) | 0.12 ( 0.02 - 0.88 ) | 6.23 | 0.13 ( 0.02 ) | -2.98 ( -5.03 ) |
| Body Temperature Abnormal | 1 | 0 (0 - 0) | Inf ( NaN - Inf ) | 31.58 | 32.58 ( 0 ) | 5.03 ( 2.14 ) |

**Supplementary Table 4.** All PTs of lecanemab among Non-AD.

| PT | N | RR(95% CI) | ROR(95%Cl) | X2 | EBGM(EBGM05) | IC(IC025) |
| --- | --- | --- | --- | --- | --- | --- |
| Headache | 93 | 9.4 (9.21 - 9.59) | 10.32 ( 8.33 - 12.78 ) | 705.22 | 9.4 ( 7.86 ) | 3.23 ( 2.92 ) |
| Infusion Related Reaction | 51 | 51.72 (51.46 - 51.99) | 54.62 ( 41.19 - 72.44 ) | 2537.27 | 51.68 ( 40.81 ) | 5.69 ( 5.28 ) |
| ARIA-E | 50 | 313052.49 (313051.78 - 313053.19) | 330580.56 ( 162085.56 - 674233.47 ) | 2387675.85 | 47754.62 ( 26303.79 ) | 15.54 ( 14.99 ) |
| Chills | 42 | 22.5 (22.21 - 22.8) | 23.5 ( 17.25 - 32.03 ) | 864.29 | 22.49 ( 17.36 ) | 4.49 ( 4.04 ) |
| ARIA-H | 42 | 147917.3 (147916.73 - 147917.87) | 154812.4 ( 86721.8 - 276365.11 ) | 1713779.14 | 40805.5 ( 25126.47 ) | 15.32 ( 14.73 ) |
| Fatigue | 37 | 3.05 (2.73 - 3.36) | 3.13 ( 2.25 - 4.35 ) | 51.52 | 3.05 ( 2.31 ) | 1.61 ( 1.13 ) |
| Pyrexia | 35 | 6.37 (6.04 - 6.69) | 6.58 ( 4.69 - 9.22 ) | 159.33 | 6.37 ( 4.8 ) | 2.67 ( 2.18 ) |
| Confusional State | 28 | 10.96 (10.59 - 11.32) | 11.26 ( 7.73 - 16.41 ) | 254.07 | 10.96 ( 8 ) | 3.45 ( 2.91 ) |
| Amyloid Related Imaging Abnormalities | 25 | 140873.62 (140872.89 - 140874.35) | 144710.02 ( 69305.16 - 302156.28 ) | 1006226.51 | 40250.32 ( 21738.9 ) | 15.3 ( 14.55 ) |
| Nausea | 24 | 1.95 (1.55 - 2.34) | 1.97 ( 1.31 - 2.96 ) | 11.18 | 1.95 ( 1.39 ) | 0.96 ( 0.38 ) |
| Tremor | 20 | 7.54 (7.11 - 7.98) | 7.68 ( 4.93 - 11.97 ) | 113.79 | 7.54 ( 5.2 ) | 2.91 ( 2.28 ) |
| Vomiting | 19 | 2.61 (2.17 - 3.06) | 2.65 ( 1.68 - 4.17 ) | 19.09 | 2.61 ( 1.79 ) | 1.39 ( 0.73 ) |
| Dizziness | 18 | 2.3 (1.84 - 2.75) | 2.32 ( 1.46 - 3.7 ) | 13.28 | 2.3 ( 1.55 ) | 1.2 ( 0.53 ) |
| Influenza Like Illness | 15 | 11.22 (10.71 - 11.72) | 11.38 ( 6.83 - 18.96 ) | 139.76 | 11.21 ( 7.32 ) | 3.49 ( 2.76 ) |
| Feeling Cold | 11 | 24.84 (24.25 - 25.43) | 25.12 ( 13.86 - 45.52 ) | 251.67 | 24.83 ( 15.1 ) | 4.63 ( 3.8 ) |
| Somnolence | 10 | 3.16 (2.55 - 3.78) | 3.18 ( 1.71 - 5.94 ) | 14.83 | 3.16 ( 1.88 ) | 1.66 ( 0.79 ) |
| Asthenia | 10 | 1.69 (1.07 - 2.3) | 1.69 ( 0.91 - 3.16 ) | 2.81 | 1.69 ( 1 ) | 0.75 ( -0.12 ) |
| Memory Impairment | 9 | 4.12 (3.47 - 4.77) | 4.15 ( 2.15 - 8.01 ) | 21.34 | 4.12 ( 2.38 ) | 2.04 ( 1.13 ) |
| Hypertension | 8 | 2.4 (1.71 - 3.09) | 2.41 ( 1.2 - 4.84 ) | 6.56 | 2.4 ( 1.34 ) | 1.26 ( 0.3 ) |
| Brain Oedema | 8 | 40.52 (39.83 - 41.21) | 40.86 ( 20.37 - 81.97 ) | 308.21 | 40.49 ( 22.61 ) | 5.34 ( 4.37 ) |
| Pain | 8 | 0.81 (0.12 - 1.5) | 0.81 ( 0.4 - 1.62 ) | 0.36 | 0.81 ( 0.45 ) | -0.3 ( -1.27 ) |
| Decreased Appetite | 7 | 1.96 (1.23 - 2.7) | 1.97 ( 0.94 - 4.15 ) | 3.33 | 1.96 ( 1.05 ) | 0.97 ( -0.05 ) |
| Arthralgia | 6 | 0.93 (0.14 - 1.73) | 0.93 ( 0.42 - 2.08 ) | 0.03 | 0.93 ( 0.48 ) | -0.1 ( -1.2 ) |
| Back Pain | 6 | 1.62 (0.83 - 2.42) | 1.63 ( 0.73 - 3.63 ) | 1.44 | 1.62 ( 0.83 ) | 0.7 ( -0.4 ) |
| Gait Disturbance | 6 | 1.95 (1.16 - 2.75) | 1.96 ( 0.88 - 4.37 ) | 2.8 | 1.95 ( 1 ) | 0.97 ( -0.13 ) |
| Rash | 6 | 0.91 (0.11 - 1.7) | 0.9 ( 0.41 - 2.02 ) | 0.06 | 0.91 ( 0.46 ) | -0.14 ( -1.24 ) |
| Fall | 6 | 1.15 (0.35 - 1.95) | 1.15 ( 0.52 - 2.57 ) | 0.12 | 1.15 ( 0.59 ) | 0.2 ( -0.89 ) |
| Cerebral Haemorrhage | 6 | 10.58 (9.78 - 11.38) | 10.64 ( 4.77 - 23.75 ) | 52.07 | 10.58 ( 5.4 ) | 3.4 ( 2.31 ) |
| Abdominal Discomfort | 5 | 1.93 (1.05 - 2.8) | 1.93 ( 0.8 - 4.65 ) | 2.23 | 1.93 ( 0.92 ) | 0.95 ( -0.24 ) |
| Pruritus | 5 | 0.91 (0.03 - 1.78) | 0.91 ( 0.38 - 2.18 ) | 0.05 | 0.91 ( 0.43 ) | -0.14 ( -1.32 ) |
| Muscle Spasms | 5 | 1.71 (0.84 - 2.59) | 1.72 ( 0.71 - 4.14 ) | 1.49 | 1.71 ( 0.82 ) | 0.78 ( -0.4 ) |
| Diarrhoea | 5 | 0.5 (-0.37 - 1.38) | 0.5 ( 0.21 - 1.2 ) | 2.5 | 0.5 ( 0.24 ) | -0.99 ( -2.18 ) |
| Seizure | 5 | 2.9 (2.03 - 3.77) | 2.91 ( 1.21 - 7.01 ) | 6.23 | 2.9 ( 1.39 ) | 1.54 ( 0.35 ) |
| Aphasia | 5 | 10.37 (9.49 - 11.24) | 10.42 ( 4.33 - 25.09 ) | 42.34 | 10.37 ( 4.97 ) | 3.37 ( 2.19 ) |
| Cerebral Microhaemorrhage | 5 | 1235.73 (1234.85 - 1236.62) | 1242.32 ( 510.98 - 3020.38 ) | 6036.32 | 1209.24 ( 575.01 ) | 10.24 ( 9.04 ) |
| Covid-19 | 5 | 1.79 (0.91 - 2.66) | 1.79 ( 0.74 - 4.31 ) | 1.73 | 1.79 ( 0.86 ) | 0.84 ( -0.35 ) |
| Heart Rate Increased | 4 | 2.57 (1.59 - 3.54) | 2.57 ( 0.96 - 6.87 ) | 3.83 | 2.57 ( 1.13 ) | 1.36 ( 0.07 ) |
| Blood Pressure Increased | 4 | 1.65 (0.67 - 2.63) | 1.65 ( 0.62 - 4.41 ) | 1.03 | 1.65 ( 0.73 ) | 0.72 ( -0.57 ) |
| Cognitive Disorder | 4 | 5.52 (4.55 - 6.5) | 5.54 ( 2.08 - 14.8 ) | 14.83 | 5.52 ( 2.43 ) | 2.47 ( 1.17 ) |
| Brain Fog | 4 | 35.25 (34.27 - 36.23) | 35.4 ( 13.25 - 94.54 ) | 133.05 | 35.23 ( 15.49 ) | 5.14 ( 3.84 ) |
| Subdural Haematoma | 4 | 17.31 (16.33 - 18.28) | 17.38 ( 6.51 - 46.4 ) | 61.45 | 17.3 ( 7.61 ) | 4.11 ( 2.82 ) |
| Drug Ineffective | 4 | 0.19 (-0.78 - 1.17) | 0.19 ( 0.07 - 0.51 ) | 13.75 | 0.19 ( 0.09 ) | -2.37 ( -3.66 ) |
| Dyspnoea | 4 | 0.45 (-0.53 - 1.43) | 0.45 ( 0.17 - 1.19 ) | 2.73 | 0.45 ( 0.2 ) | -1.16 ( -2.45 ) |
| Syncope | 4 | 2.52 (1.54 - 3.49) | 2.52 ( 0.94 - 6.74 ) | 3.66 | 2.52 ( 1.11 ) | 1.33 ( 0.04 ) |
| Amnesia | 4 | 3.81 (2.83 - 4.79) | 3.82 ( 1.43 - 10.21 ) | 8.3 | 3.81 ( 1.68 ) | 1.93 ( 0.64 ) |
| Loss Of Consciousness | 4 | 1.97 (1 - 2.95) | 1.98 ( 0.74 - 5.28 ) | 1.92 | 1.97 ( 0.87 ) | 0.98 ( -0.31 ) |
| Hypotension | 4 | 1.26 (0.29 - 2.24) | 1.27 ( 0.47 - 3.38 ) | 0.22 | 1.26 ( 0.56 ) | 0.34 ( -0.96 ) |
| Balance Disorder | 4 | 2.89 (1.91 - 3.87) | 2.9 ( 1.09 - 7.74 ) | 4.95 | 2.89 ( 1.27 ) | 1.53 ( 0.24 ) |
| Atrial Fibrillation | 4 | 2.59 (1.62 - 3.57) | 2.6 ( 0.97 - 6.94 ) | 3.92 | 2.59 ( 1.14 ) | 1.37 ( 0.08 ) |
| Body Temperature Increased | 3 | 9.04 (7.91 - 10.17) | 9.07 ( 2.92 - 28.17 ) | 21.46 | 9.04 ( 3.5 ) | 3.18 ( 1.73 ) |
| Abdominal Pain Upper | 3 | 0.94 (-0.19 - 2.07) | 0.94 ( 0.3 - 2.92 ) | 0.01 | 0.94 ( 0.36 ) | -0.09 ( -1.53 ) |
| Feeling Abnormal | 3 | 0.77 (-0.36 - 1.9) | 0.77 ( 0.25 - 2.38 ) | 0.21 | 0.77 ( 0.3 ) | -0.38 ( -1.83 ) |
| Lethargy | 3 | 3.28 (2.15 - 4.41) | 3.28 ( 1.06 - 10.2 ) | 4.75 | 3.28 ( 1.27 ) | 1.71 ( 0.27 ) |
| Disorientation | 3 | 4.7 (3.57 - 5.83) | 4.71 ( 1.52 - 14.64 ) | 8.75 | 4.7 ( 1.82 ) | 2.23 ( 0.79 ) |
| Blood Pressure Decreased | 3 | 2.87 (1.74 - 4) | 2.88 ( 0.93 - 8.94 ) | 3.66 | 2.87 ( 1.11 ) | 1.52 ( 0.08 ) |
| Migraine | 3 | 2.07 (0.94 - 3.2) | 2.07 ( 0.67 - 6.43 ) | 1.65 | 2.07 ( 0.8 ) | 1.05 ( -0.4 ) |
| Pain In Extremity | 3 | 0.63 (-0.5 - 1.76) | 0.63 ( 0.2 - 1.95 ) | 0.66 | 0.63 ( 0.24 ) | -0.67 ( -2.11 ) |
| Gait Inability | 3 | 6.35 (5.22 - 7.48) | 6.37 ( 2.05 - 19.79 ) | 13.54 | 6.35 ( 2.46 ) | 2.67 ( 1.22 ) |
| Head Discomfort | 3 | 11.15 (10.02 - 12.28) | 11.18 ( 3.6 - 34.73 ) | 27.71 | 11.14 ( 4.32 ) | 3.48 ( 2.03 ) |
| Insomnia | 3 | 0.7 (-0.43 - 1.83) | 0.7 ( 0.23 - 2.19 ) | 0.37 | 0.7 ( 0.27 ) | -0.5 ( -1.95 ) |
| Hypoaesthesia | 3 | 1.25 (0.12 - 2.38) | 1.25 ( 0.4 - 3.9 ) | 0.15 | 1.25 ( 0.49 ) | 0.33 ( -1.12 ) |
| Speech Disorder | 3 | 3.6 (2.47 - 4.73) | 3.6 ( 1.16 - 11.2 ) | 5.63 | 3.6 ( 1.39 ) | 1.85 ( 0.4 ) |
| Chest Pain | 3 | 1.01 (-0.12 - 2.14) | 1.01 ( 0.32 - 3.14 ) | 0 | 1.01 ( 0.39 ) | 0.01 ( -1.43 ) |
| Visual Impairment | 3 | 1.6 (0.47 - 2.73) | 1.6 ( 0.52 - 4.97 ) | 0.67 | 1.6 ( 0.62 ) | 0.68 ( -0.77 ) |
| Ischaemic Stroke | 3 | 10.55 (9.42 - 11.68) | 10.58 ( 3.41 - 32.87 ) | 25.94 | 10.55 ( 4.09 ) | 3.4 ( 1.95 ) |
| Deep Vein Thrombosis | 3 | 2.8 (1.67 - 3.93) | 2.81 ( 0.9 - 8.72 ) | 3.48 | 2.8 ( 1.09 ) | 1.49 ( 0.04 ) |
| Death | 3 | 0.22 (-0.91 - 1.35) | 0.22 ( 0.07 - 0.69 ) | 8.13 | 0.22 ( 0.09 ) | -2.15 ( -3.6 ) |
| Cerebral Infarction | 3 | 7.67 (6.54 - 8.8) | 7.69 ( 2.48 - 23.9 ) | 17.41 | 7.67 ( 2.97 ) | 2.94 ( 1.49 ) |
| Upper Respiratory Tract Infection | 2 | 2.78 (1.39 - 4.16) | 2.78 ( 0.69 - 11.14 ) | 2.28 | 2.78 ( 0.87 ) | 1.47 ( -0.19 ) |
| Depressed Mood | 2 | 2.46 (1.08 - 3.85) | 2.47 ( 0.62 - 9.87 ) | 1.74 | 2.46 ( 0.77 ) | 1.3 ( -0.37 ) |
| Ear Pain | 2 | 6.53 (5.14 - 7.91) | 6.54 ( 1.63 - 26.19 ) | 9.37 | 6.53 ( 2.04 ) | 2.71 ( 1.04 ) |
| Dehydration | 2 | 0.94 (-0.44 - 2.33) | 0.94 ( 0.24 - 3.78 ) | 0.01 | 0.94 ( 0.3 ) | -0.08 ( -1.75 ) |
| Condition Aggravated | 2 | 0.44 (-0.94 - 1.83) | 0.44 ( 0.11 - 1.77 ) | 1.41 | 0.44 ( 0.14 ) | -1.18 ( -2.84 ) |
| Dyskinesia | 2 | 3.11 (1.72 - 4.49) | 3.11 ( 0.78 - 12.46 ) | 2.86 | 3.11 ( 0.97 ) | 1.63 ( -0.03 ) |
| Rash Pruritic | 2 | 2.44 (1.05 - 3.82) | 2.44 ( 0.61 - 9.78 ) | 1.7 | 2.44 ( 0.76 ) | 1.29 ( -0.38 ) |
| Poor Quality Sleep | 2 | 6.07 (4.69 - 7.45) | 6.08 ( 1.52 - 24.35 ) | 8.47 | 6.07 ( 1.9 ) | 2.6 ( 0.93 ) |
| Lacrimation Increased | 2 | 4.48 (3.09 - 5.86) | 4.49 ( 1.12 - 17.96 ) | 5.41 | 4.48 ( 1.4 ) | 2.16 ( 0.49 ) |
| Presyncope | 2 | 5.33 (3.94 - 6.71) | 5.34 ( 1.33 - 21.38 ) | 7.04 | 5.33 ( 1.67 ) | 2.41 ( 0.75 ) |
| Infusion Site Reaction | 2 | 56.27 (54.88 - 57.65) | 56.38 ( 14.07 - 225.94 ) | 108.46 | 56.21 ( 17.6 ) | 5.81 ( 4.14 ) |
| Weight Decreased | 2 | 0.45 (-0.93 - 1.84) | 0.45 ( 0.11 - 1.82 ) | 1.31 | 0.45 ( 0.14 ) | -1.14 ( -2.8 ) |
| Incontinence | 2 | 11.97 (10.59 - 13.36) | 12 ( 3 - 48.04 ) | 20.11 | 11.97 ( 3.75 ) | 3.58 ( 1.91 ) |
| Urinary Tract Infection | 2 | 0.76 (-0.63 - 2.14) | 0.76 ( 0.19 - 3.03 ) | 0.16 | 0.76 ( 0.24 ) | -0.4 ( -2.07 ) |
| Cough | 2 | 0.46 (-0.92 - 1.85) | 0.46 ( 0.12 - 1.85 ) | 1.25 | 0.46 ( 0.14 ) | -1.11 ( -2.78 ) |
| Feeling Hot | 2 | 2.05 (0.66 - 3.43) | 2.05 ( 0.51 - 8.22 ) | 1.08 | 2.05 ( 0.64 ) | 1.04 ( -0.63 ) |
| Bradycardia | 2 | 2.36 (0.97 - 3.74) | 2.36 ( 0.59 - 9.45 ) | 1.56 | 2.36 ( 0.74 ) | 1.24 ( -0.43 ) |
| Myocardial Ischaemia | 2 | 10.35 (8.97 - 11.74) | 10.37 ( 2.59 - 41.55 ) | 16.9 | 10.35 ( 3.24 ) | 3.37 ( 1.7 ) |
| Anxiety | 2 | 0.44 (-0.95 - 1.82) | 0.44 ( 0.11 - 1.75 ) | 1.45 | 0.44 ( 0.14 ) | -1.19 ( -2.86 ) |
| Restlessness | 2 | 3.43 (2.05 - 4.82) | 3.44 ( 0.86 - 13.77 ) | 3.45 | 3.43 ( 1.07 ) | 1.78 ( 0.11 ) |
| Weight Increased | 2 | 0.58 (-0.81 - 1.96) | 0.58 ( 0.14 - 2.31 ) | 0.62 | 0.58 ( 0.18 ) | -0.79 ( -2.46 ) |
| Swollen Tongue | 2 | 4.02 (2.64 - 5.41) | 4.03 ( 1.01 - 16.13 ) | 4.54 | 4.02 ( 1.26 ) | 2.01 ( 0.34 ) |
| Cerebrovascular Accident | 2 | 0.73 (-0.66 - 2.11) | 0.73 ( 0.18 - 2.91 ) | 0.2 | 0.73 ( 0.23 ) | -0.46 ( -2.13 ) |
| Superficial Siderosis Of Central Nervous System | 2 | 4507.96 (4506.52 - 4509.39) | 4517.53 ( 1068.49 - 19099.9 ) | 8344.37 | 4174.11 ( 1249.28 ) | 12.03 ( 10.27 ) |
| Hypersomnia | 2 | 4.48 (3.09 - 5.86) | 4.48 ( 1.12 - 17.96 ) | 5.4 | 4.48 ( 1.4 ) | 2.16 ( 0.49 ) |
| Infusion Site Extravasation | 2 | 20.23 (18.85 - 21.62) | 20.27 ( 5.06 - 81.21 ) | 36.56 | 20.23 ( 6.33 ) | 4.34 ( 2.67 ) |
| Delusion | 2 | 8.34 (6.95 - 9.72) | 8.35 ( 2.09 - 33.45 ) | 12.91 | 8.33 ( 2.61 ) | 3.06 ( 1.39 ) |
| Hypersensitivity | 2 | 0.69 (-0.7 - 2.07) | 0.69 ( 0.17 - 2.75 ) | 0.29 | 0.69 ( 0.22 ) | -0.54 ( -2.21 ) |
| Oedema Peripheral | 2 | 1 (-0.38 - 2.39) | 1 ( 0.25 - 4.02 ) | 0 | 1 ( 0.31 ) | 0.01 ( -1.66 ) |
| Blood Glucose Increased | 2 | 0.65 (-0.73 - 2.04) | 0.65 ( 0.16 - 2.62 ) | 0.37 | 0.65 ( 0.2 ) | -0.61 ( -2.28 ) |
| Magnetic Resonance Imaging Abnormal | 2 | 117.76 (116.38 - 119.15) | 118.01 ( 29.43 - 473.25 ) | 231.06 | 117.52 ( 36.76 ) | 6.88 ( 5.21 ) |
| Palpitations | 1 | 0.54 (-1.42 - 2.5) | 0.54 ( 0.08 - 3.85 ) | 0.39 | 0.54 ( 0.11 ) | -0.88 ( -2.92 ) |
| Neck Pain | 1 | 1.13 (-0.83 - 3.09) | 1.13 ( 0.16 - 8.06 ) | 0.02 | 1.13 ( 0.22 ) | 0.18 ( -1.86 ) |
| Constipation | 1 | 0.3 (-1.65 - 2.26) | 0.3 ( 0.04 - 2.16 ) | 1.59 | 0.3 ( 0.06 ) | -1.71 ( -3.76 ) |
| Sars-Cov-2 Test Positive | 1 | 4.54 (2.58 - 6.5) | 4.54 ( 0.64 - 32.3 ) | 2.76 | 4.54 ( 0.88 ) | 2.18 ( 0.14 ) |
| Blood Magnesium Decreased | 1 | 7.41 (5.45 - 9.37) | 7.42 ( 1.04 - 52.72 ) | 5.55 | 7.41 ( 1.44 ) | 2.89 ( 0.85 ) |
| Abnormal Dreams | 1 | 2.21 (0.25 - 4.17) | 2.21 ( 0.31 - 15.71 ) | 0.66 | 2.21 ( 0.43 ) | 1.14 ( -0.9 ) |
| Delusional Perception | 1 | 149.87 (147.9 - 151.83) | 150.02 ( 21.06 - 1068.95 ) | 147.48 | 149.47 ( 28.91 ) | 7.22 ( 5.18 ) |
| Rash Papular | 1 | 2.75 (0.79 - 4.71) | 2.75 ( 0.39 - 19.58 ) | 1.12 | 2.75 ( 0.53 ) | 1.46 ( -0.58 ) |
| Urinary Incontinence | 1 | 2.13 (0.17 - 4.09) | 2.13 ( 0.3 - 15.16 ) | 0.6 | 2.13 ( 0.41 ) | 1.09 ( -0.95 ) |
| Encephalitis | 1 | 9.49 (7.54 - 11.45) | 9.5 ( 1.34 - 67.55 ) | 7.6 | 9.49 ( 1.84 ) | 3.25 ( 1.2 ) |
| Eye Pruritus | 1 | 2.14 (0.18 - 4.1) | 2.14 ( 0.3 - 15.21 ) | 0.61 | 2.14 ( 0.41 ) | 1.1 ( -0.95 ) |
| Ocular Hyperaemia | 1 | 1.45 (-0.51 - 3.41) | 1.45 ( 0.2 - 10.32 ) | 0.14 | 1.45 ( 0.28 ) | 0.54 ( -1.51 ) |
| Hallucination | 1 | 0.87 (-1.09 - 2.83) | 0.87 ( 0.12 - 6.17 ) | 0.02 | 0.87 ( 0.17 ) | -0.2 ( -2.25 ) |
| Concussion | 1 | 8.31 (6.35 - 10.27) | 8.32 ( 1.17 - 59.15 ) | 6.43 | 8.31 ( 1.61 ) | 3.06 ( 1.01 ) |
| Nasal Congestion | 1 | 1.13 (-0.83 - 3.08) | 1.13 ( 0.16 - 8 ) | 0.01 | 1.13 ( 0.22 ) | 0.17 ( -1.87 ) |
| Heart Rate Decreased | 1 | 1.77 (-0.19 - 3.73) | 1.77 ( 0.25 - 12.57 ) | 0.33 | 1.77 ( 0.34 ) | 0.82 ( -1.22 ) |
| Atrioventricular Block | 1 | 8.25 (6.29 - 10.21) | 8.26 ( 1.16 - 58.7 ) | 6.37 | 8.25 ( 1.6 ) | 3.04 ( 1 ) |
| Panic Reaction | 1 | 8.72 (6.76 - 10.68) | 8.73 ( 1.23 - 62.02 ) | 6.83 | 8.72 ( 1.69 ) | 3.12 ( 1.08 ) |
| Pneumonia | 1 | 0.2 (-1.76 - 2.16) | 0.2 ( 0.03 - 1.42 ) | 3.2 | 0.2 ( 0.04 ) | -2.32 ( -4.36 ) |
| Thinking Abnormal | 1 | 3.26 (1.3 - 5.21) | 3.26 ( 0.46 - 23.16 ) | 1.56 | 3.26 ( 0.63 ) | 1.7 ( -0.34 ) |
| Retching | 1 | 3.02 (1.07 - 4.98) | 3.03 ( 0.43 - 21.51 ) | 1.36 | 3.02 ( 0.59 ) | 1.6 ( -0.45 ) |
| Muscle Tightness | 1 | 3.86 (1.9 - 5.81) | 3.86 ( 0.54 - 27.42 ) | 2.12 | 3.86 ( 0.75 ) | 1.95 ( -0.1 ) |
| Bone Pain | 1 | 1.08 (-0.88 - 3.03) | 1.08 ( 0.15 - 7.65 ) | 0.01 | 1.08 ( 0.21 ) | 0.11 ( -1.94 ) |
| Erectile Dysfunction | 1 | 2.5 (0.54 - 4.46) | 2.5 ( 0.35 - 17.79 ) | 0.9 | 2.5 ( 0.48 ) | 1.32 ( -0.72 ) |
| Rib Fracture | 1 | 3.1 (1.14 - 5.06) | 3.11 ( 0.44 - 22.07 ) | 1.43 | 3.1 ( 0.6 ) | 1.63 ( -0.41 ) |
| Tooth Fracture | 1 | 5.65 (3.69 - 7.61) | 5.66 ( 0.8 - 40.19 ) | 3.83 | 5.65 ( 1.09 ) | 2.5 ( 0.46 ) |
| Hemiplegia | 1 | 7.46 (5.5 - 9.42) | 7.47 ( 1.05 - 53.08 ) | 5.59 | 7.46 ( 1.45 ) | 2.9 ( 0.86 ) |
| Osteoarthritis | 1 | 1.51 (-0.45 - 3.47) | 1.51 ( 0.21 - 10.72 ) | 0.17 | 1.51 ( 0.29 ) | 0.59 ( -1.45 ) |
| Ventricular Fibrillation | 1 | 5.58 (3.62 - 7.54) | 5.59 ( 0.79 - 39.71 ) | 3.76 | 5.58 ( 1.08 ) | 2.48 ( 0.44 ) |
| Electrocardiogram St Segment Elevation | 1 | 18.44 (16.48 - 20.4) | 18.46 ( 2.6 - 131.21 ) | 16.49 | 18.43 ( 3.57 ) | 4.2 ( 2.16 ) |
| Skin Irritation | 1 | 1.35 (-0.61 - 3.31) | 1.35 ( 0.19 - 9.61 ) | 0.09 | 1.35 ( 0.26 ) | 0.43 ( -1.61 ) |
| Abdominal Pain | 1 | 0.27 (-1.68 - 2.23) | 0.27 ( 0.04 - 1.95 ) | 1.92 | 0.27 ( 0.05 ) | -1.86 ( -3.91 ) |
| Language Disorder | 1 | 29.35 (27.39 - 31.31) | 29.38 ( 4.13 - 208.89 ) | 27.37 | 29.33 ( 5.68 ) | 4.87 ( 2.83 ) |
| Blood Pressure Measurement | 1 | 112.7 (110.74 - 114.66) | 112.82 ( 15.84 - 803.33 ) | 110.49 | 112.48 ( 21.76 ) | 6.81 ( 4.77 ) |
| Myocardial Infarction | 1 | 0.34 (-1.62 - 2.3) | 0.34 ( 0.05 - 2.43 ) | 1.27 | 0.34 ( 0.07 ) | -1.55 ( -3.59 ) |
| Conjunctivitis | 1 | 3.65 (1.69 - 5.61) | 3.65 ( 0.51 - 25.95 ) | 1.92 | 3.65 ( 0.71 ) | 1.87 ( -0.18 ) |
| Formication | 1 | 8.78 (6.82 - 10.74) | 8.79 ( 1.24 - 62.46 ) | 6.89 | 8.78 ( 1.7 ) | 3.13 ( 1.09 ) |
| Hospitalisation | 1 | 0.44 (-1.52 - 2.4) | 0.44 ( 0.06 - 3.13 ) | 0.71 | 0.44 ( 0.09 ) | -1.18 ( -3.22 ) |
| Paralysis | 1 | 4.36 (2.4 - 6.32) | 4.36 ( 0.61 - 31.01 ) | 2.59 | 4.36 ( 0.84 ) | 2.12 ( 0.08 ) |
| Multiple Organ Dysfunction Syndrome | 1 | 2.53 (0.57 - 4.49) | 2.53 ( 0.36 - 17.98 ) | 0.92 | 2.53 ( 0.49 ) | 1.34 ( -0.7 ) |
| Anger | 1 | 1.82 (-0.14 - 3.77) | 1.82 ( 0.26 - 12.91 ) | 0.37 | 1.82 ( 0.35 ) | 0.86 ( -1.18 ) |
| Myocardial Necrosis Marker Increased | 1 | 43.78 (41.82 - 45.74) | 43.83 ( 6.16 - 311.72 ) | 41.77 | 43.75 ( 8.47 ) | 5.45 ( 3.41 ) |
| Vaginal Haemorrhage | 1 | 1.43 (-0.53 - 3.39) | 1.43 ( 0.2 - 10.17 ) | 0.13 | 1.43 ( 0.28 ) | 0.52 ( -1.53 ) |
| Loss Of Personal Independence In Daily Activities | 1 | 1.45 (-0.51 - 3.41) | 1.45 ( 0.2 - 10.28 ) | 0.14 | 1.45 ( 0.28 ) | 0.53 ( -1.51 ) |
| Primary Stabbing Headache | 1 | 2253.98 (2251.98 - 2255.98) | 2256.37 ( 305.42 - 16669.7 ) | 2165.36 | 2167.32 ( 406.64 ) | 11.08 ( 8.97 ) |
| Psychotic Behaviour | 1 | 59.57 (57.61 - 61.53) | 59.63 ( 8.38 - 424.2 ) | 57.52 | 59.5 ( 11.52 ) | 5.89 ( 3.85 ) |
| Cerebral Thrombosis | 1 | 22.03 (20.07 - 23.99) | 22.05 ( 3.1 - 156.77 ) | 20.07 | 22.02 ( 4.27 ) | 4.46 ( 2.42 ) |
| Skin Discolouration | 1 | 1.38 (-0.58 - 3.34) | 1.38 ( 0.19 - 9.78 ) | 0.1 | 1.38 ( 0.27 ) | 0.46 ( -1.58 ) |
| Chest Discomfort | 1 | 0.63 (-1.33 - 2.59) | 0.63 ( 0.09 - 4.5 ) | 0.21 | 0.63 ( 0.12 ) | -0.66 ( -2.7 ) |
| Troponin Increased | 1 | 9.13 (7.18 - 11.09) | 9.14 ( 1.29 - 64.99 ) | 7.24 | 9.13 ( 1.77 ) | 3.19 ( 1.15 ) |
| Appetite Disorder | 1 | 10.22 (8.26 - 12.18) | 10.23 ( 1.44 - 72.7 ) | 8.31 | 10.22 ( 1.98 ) | 3.35 ( 1.31 ) |
| Dysuria | 1 | 1.7 (-0.26 - 3.66) | 1.7 ( 0.24 - 12.08 ) | 0.29 | 1.7 ( 0.33 ) | 0.77 ( -1.28 ) |
| Back Injury | 1 | 6.17 (4.21 - 8.13) | 6.18 ( 0.87 - 43.9 ) | 4.33 | 6.17 ( 1.2 ) | 2.63 ( 0.58 ) |
| Road Traffic Accident | 1 | 1.53 (-0.43 - 3.48) | 1.53 ( 0.21 - 10.84 ) | 0.18 | 1.53 ( 0.3 ) | 0.61 ( -1.43 ) |
| Burning Sensation | 1 | 0.9 (-1.06 - 2.85) | 0.9 ( 0.13 - 6.36 ) | 0.01 | 0.9 ( 0.17 ) | -0.16 ( -2.2 ) |
| Vision Blurred | 1 | 0.47 (-1.49 - 2.43) | 0.47 ( 0.07 - 3.34 ) | 0.6 | 0.47 ( 0.09 ) | -1.09 ( -3.13 ) |
| Hot Flush | 1 | 0.9 (-1.06 - 2.85) | 0.9 ( 0.13 - 6.36 ) | 0.01 | 0.9 ( 0.17 ) | -0.16 ( -2.2 ) |
| Night Sweats | 1 | 2.04 (0.08 - 4) | 2.04 ( 0.29 - 14.51 ) | 0.53 | 2.04 ( 0.4 ) | 1.03 ( -1.01 ) |
| Pharyngeal Swelling | 1 | 7.54 (5.58 - 9.5) | 7.55 ( 1.06 - 53.67 ) | 5.68 | 7.54 ( 1.46 ) | 2.92 ( 0.87 ) |
| Nasopharyngitis | 1 | 0.35 (-1.61 - 2.31) | 0.35 ( 0.05 - 2.5 ) | 1.19 | 0.35 ( 0.07 ) | -1.5 ( -3.55 ) |
| Joint Swelling | 1 | 0.54 (-1.42 - 2.49) | 0.53 ( 0.08 - 3.8 ) | 0.4 | 0.54 ( 0.1 ) | -0.9 ( -2.94 ) |
| Lacunar Stroke | 1 | 215.07 (213.11 - 217.04) | 215.3 ( 30.18 - 1535.81 ) | 212.27 | 214.26 ( 41.4 ) | 7.74 ( 5.69 ) |
| Panic Attack | 1 | 1.73 (-0.23 - 3.69) | 1.73 ( 0.24 - 12.33 ) | 0.31 | 1.73 ( 0.34 ) | 0.79 ( -1.25 ) |
| Vasogenic Cerebral Oedema | 1 | 96 (94.04 - 97.96) | 96.1 ( 13.5 - 684.07 ) | 93.85 | 95.83 ( 18.55 ) | 6.58 ( 4.54 ) |
| Mental Impairment | 1 | 2.59 (0.64 - 4.55) | 2.6 ( 0.37 - 18.45 ) | 0.98 | 2.59 ( 0.5 ) | 1.38 ( -0.67 ) |
| Urticaria | 1 | 0.4 (-1.56 - 2.35) | 0.39 ( 0.06 - 2.8 ) | 0.93 | 0.4 ( 0.08 ) | -1.34 ( -3.38 ) |
| Transfusion Reaction | 1 | 74.44 (72.48 - 76.4) | 74.52 ( 10.47 - 530.25 ) | 72.36 | 74.34 ( 14.39 ) | 6.22 ( 4.17 ) |
| Subcortical Stroke | 1 | 28174.72 (28172.32 - 28177.12) | 28204.63 ( 2555.22 - 311324.41 ) | 18781.82 | 18783.48 ( 2518.56 ) | 14.2 ( 11.7 ) |
| Breast Cancer | 1 | 0.63 (-1.33 - 2.59) | 0.63 ( 0.09 - 4.48 ) | 0.22 | 0.63 ( 0.12 ) | -0.67 ( -2.71 ) |
| Sars-Cov-2 Antibody Test Positive | 1 | 227.22 (225.25 - 229.18) | 227.46 ( 31.88 - 1622.85 ) | 224.32 | 226.31 ( 43.72 ) | 7.82 ( 5.77 ) |
| Intestinal Perforation | 1 | 5.73 (3.77 - 7.69) | 5.73 ( 0.81 - 40.74 ) | 3.9 | 5.73 ( 1.11 ) | 2.52 ( 0.48 ) |
| Thrombocytopenia | 1 | 0.57 (-1.39 - 2.53) | 0.57 ( 0.08 - 4.07 ) | 0.32 | 0.57 ( 0.11 ) | -0.8 ( -2.84 ) |
| Crying | 1 | 1.68 (-0.28 - 3.64) | 1.68 ( 0.24 - 11.92 ) | 0.27 | 1.68 ( 0.32 ) | 0.75 ( -1.3 ) |
| Oral Mucosal Blistering | 1 | 9.81 (7.85 - 11.77) | 9.82 ( 1.38 - 69.78 ) | 7.91 | 9.81 ( 1.9 ) | 3.29 ( 1.25 ) |
| Tachycardia | 1 | 0.72 (-1.24 - 2.67) | 0.72 ( 0.1 - 5.09 ) | 0.11 | 0.72 ( 0.14 ) | -0.48 ( -2.52 ) |
| Poor Venous Access | 1 | 6.41 (4.45 - 8.37) | 6.42 ( 0.9 - 45.62 ) | 4.57 | 6.41 ( 1.24 ) | 2.68 ( 0.64 ) |
| Central Nervous System Lesion | 1 | 4.36 (2.4 - 6.32) | 4.36 ( 0.61 - 30.99 ) | 2.59 | 4.36 ( 0.84 ) | 2.12 ( 0.08 ) |
| Thunderclap Headache | 1 | 274.88 (272.91 - 276.84) | 275.17 ( 38.53 - 1964.88 ) | 271.56 | 273.55 ( 52.81 ) | 8.1 ( 6.04 ) |
| Therapy Cessation | 1 | 1.27 (-0.69 - 3.23) | 1.27 ( 0.18 - 9.02 ) | 0.06 | 1.27 ( 0.25 ) | 0.34 ( -1.7 ) |
| Haemorrhagic Stroke | 1 | 7.84 (5.88 - 9.8) | 7.84 ( 1.1 - 55.75 ) | 5.96 | 7.84 ( 1.52 ) | 2.97 ( 0.93 ) |
| Respiratory Tract Infection | 1 | 2.56 (0.6 - 4.52) | 2.56 ( 0.36 - 18.18 ) | 0.95 | 2.56 ( 0.5 ) | 1.35 ( -0.69 ) |
| Oxygen Saturation Decreased | 1 | 1.19 (-0.77 - 3.14) | 1.19 ( 0.17 - 8.43 ) | 0.03 | 1.19 ( 0.23 ) | 0.25 ( -1.8 ) |
| Pallor | 1 | 2.29 (0.33 - 4.25) | 2.29 ( 0.32 - 16.28 ) | 0.73 | 2.29 ( 0.44 ) | 1.19 ( -0.85 ) |
| Extravasation | 1 | 15.16 (13.2 - 17.12) | 15.18 ( 2.14 - 107.9 ) | 13.23 | 15.16 ( 2.94 ) | 3.92 ( 1.88 ) |
| Faeces Soft | 1 | 9.65 (7.69 - 11.61) | 9.66 ( 1.36 - 68.67 ) | 7.76 | 9.65 ( 1.87 ) | 3.27 ( 1.23 ) |
| Aneurysm | 1 | 12.71 (10.75 - 14.66) | 12.72 ( 1.79 - 90.4 ) | 10.78 | 12.7 ( 2.46 ) | 3.67 ( 1.62 ) |
| Stomatitis | 1 | 1.06 (-0.9 - 3.02) | 1.06 ( 0.15 - 7.52 ) | 0 | 1.06 ( 0.21 ) | 0.08 ( -1.96 ) |
| Blood Cholesterol Increased | 1 | 1.38 (-0.58 - 3.34) | 1.38 ( 0.19 - 9.79 ) | 0.1 | 1.38 ( 0.27 ) | 0.46 ( -1.58 ) |
| Retinal Haemorrhage | 1 | 8.93 (6.97 - 10.89) | 8.93 ( 1.26 - 63.5 ) | 7.04 | 8.92 ( 1.73 ) | 3.16 ( 1.12 ) |
| Abnormal Behaviour | 1 | 1.53 (-0.43 - 3.49) | 1.53 ( 0.22 - 10.87 ) | 0.18 | 1.53 ( 0.3 ) | 0.61 ( -1.43 ) |
| Sinus Congestion | 1 | 4.68 (2.72 - 6.64) | 4.68 ( 0.66 - 33.29 ) | 2.89 | 4.68 ( 0.91 ) | 2.23 ( 0.18 ) |
| Rhinorrhoea | 1 | 1.01 (-0.95 - 2.97) | 1.01 ( 0.14 - 7.18 ) | 0 | 1.01 ( 0.2 ) | 0.02 ( -2.03 ) |
| Neuralgia | 1 | 2.66 (0.7 - 4.62) | 2.66 ( 0.37 - 18.9 ) | 1.03 | 2.66 ( 0.52 ) | 1.41 ( -0.63 ) |
| Transient Ischaemic Attack | 1 | 1.82 (-0.14 - 3.78) | 1.82 ( 0.26 - 12.95 ) | 0.37 | 1.82 ( 0.35 ) | 0.86 ( -1.18 ) |
| Head Injury | 1 | 2.04 (0.08 - 4) | 2.04 ( 0.29 - 14.51 ) | 0.53 | 2.04 ( 0.4 ) | 1.03 ( -1.01 ) |
| Visual Brightness | 1 | 155.23 (153.27 - 157.19) | 155.4 ( 21.81 - 1107.34 ) | 152.82 | 154.81 ( 29.94 ) | 7.27 ( 5.23 ) |
| Venous Thrombosis Limb | 1 | 27.73 (25.77 - 29.69) | 27.76 ( 3.9 - 197.37 ) | 25.76 | 27.72 ( 5.37 ) | 4.79 ( 2.75 ) |
| Inflammation | 1 | 1.29 (-0.67 - 3.25) | 1.29 ( 0.18 - 9.16 ) | 0.06 | 1.29 ( 0.25 ) | 0.37 ( -1.68 ) |
| Erythema | 1 | 0.31 (-1.65 - 2.27) | 0.31 ( 0.04 - 2.2 ) | 1.54 | 0.31 ( 0.06 ) | -1.69 ( -3.73 ) |
| Acute Kidney Injury | 1 | 0.43 (-1.53 - 2.39) | 0.43 ( 0.06 - 3.05 ) | 0.76 | 0.43 ( 0.08 ) | -1.22 ( -3.26 ) |
| Prostatitis | 1 | 20.43 (18.47 - 22.39) | 20.45 ( 2.88 - 145.4 ) | 18.47 | 20.42 ( 3.96 ) | 4.35 ( 2.31 ) |
| Hospice Care | 1 | 6.77 (4.82 - 8.73) | 6.78 ( 0.95 - 48.19 ) | 4.92 | 6.77 ( 1.31 ) | 2.76 ( 0.72 ) |
| Inguinal Mass | 1 | 254.97 (253.01 - 256.94) | 255.24 ( 35.76 - 1822 ) | 251.84 | 253.83 ( 49.01 ) | 7.99 ( 5.94 ) |
| Malaise | 1 | 0.14 (-1.82 - 2.1) | 0.14 ( 0.02 - 1 ) | 5.22 | 0.14 ( 0.03 ) | -2.82 ( -4.86 ) |
| Cataract | 1 | 1.1 (-0.86 - 3.06) | 1.1 ( 0.16 - 7.84 ) | 0.01 | 1.1 ( 0.21 ) | 0.14 ( -1.9 ) |
| Menstrual Disorder | 1 | 7.77 (5.81 - 9.73) | 7.78 ( 1.09 - 55.29 ) | 5.9 | 7.77 ( 1.51 ) | 2.96 ( 0.92 ) |
| Hyperhidrosis | 1 | 0.48 (-1.47 - 2.44) | 0.48 ( 0.07 - 3.44 ) | 0.55 | 0.48 ( 0.09 ) | -1.05 ( -3.09 ) |
| Dizziness Postural | 1 | 7.05 (5.09 - 9.01) | 7.05 ( 0.99 - 50.14 ) | 5.19 | 7.05 ( 1.37 ) | 2.82 ( 0.77 ) |
| Coronary Artery Disease | 1 | 1.92 (-0.04 - 3.88) | 1.92 ( 0.27 - 13.67 ) | 0.44 | 1.92 ( 0.37 ) | 0.94 ( -1.1 ) |
| Cerebral Microinfarction | 1 | 1280.67 (1278.69 - 1282.65) | 1282.03 ( 176.45 - 9314.94 ) | 1250.26 | 1252.23 ( 238.24 ) | 10.29 ( 8.2 ) |
| Eyelid Rash | 1 | 81.55 (79.59 - 83.51) | 81.63 ( 11.47 - 580.97 ) | 79.45 | 81.43 ( 15.76 ) | 6.35 ( 4.3 ) |
| Epistaxis | 1 | 0.84 (-1.12 - 2.8) | 0.84 ( 0.12 - 5.97 ) | 0.03 | 0.84 ( 0.16 ) | -0.25 ( -2.29 ) |
| Platelet Count Decreased | 1 | 0.59 (-1.37 - 2.55) | 0.59 ( 0.08 - 4.21 ) | 0.28 | 0.59 ( 0.11 ) | -0.75 ( -2.8 ) |
| Depression | 1 | 0.27 (-1.69 - 2.23) | 0.27 ( 0.04 - 1.92 ) | 1.98 | 0.27 ( 0.05 ) | -1.89 ( -3.93 ) |
| Eating Disorder | 1 | 2.95 (0.99 - 4.9) | 2.95 ( 0.41 - 20.95 ) | 1.29 | 2.95 ( 0.57 ) | 1.56 ( -0.48 ) |
| Dementia Alzheimer'S Type | 1 | 7.54 (5.58 - 9.5) | 7.55 ( 1.06 - 53.66 ) | 5.68 | 7.54 ( 1.46 ) | 2.91 ( 0.87 ) |
| Peripheral Artery Aneurysm | 1 | 151.07 (149.11 - 153.03) | 151.23 ( 21.22 - 1077.57 ) | 148.68 | 150.67 ( 29.14 ) | 7.24 ( 5.19 ) |
| Delusion Of Replacement | 1 | 782.63 (780.66 - 784.6) | 783.46 ( 108.76 - 5643.83 ) | 769.94 | 771.92 ( 147.92 ) | 9.59 ( 7.52 ) |
| Oedema | 1 | 1.19 (-0.77 - 3.15) | 1.19 ( 0.17 - 8.47 ) | 0.03 | 1.19 ( 0.23 ) | 0.25 ( -1.79 ) |
| Cerebral Mass Effect | 1 | 242.89 (240.92 - 244.85) | 243.14 ( 34.07 - 1735.25 ) | 239.86 | 241.85 ( 46.71 ) | 7.92 ( 5.87 ) |
| Therapy Interrupted | 1 | 1.17 (-0.79 - 3.13) | 1.17 ( 0.16 - 8.33 ) | 0.03 | 1.17 ( 0.23 ) | 0.23 ( -1.81 ) |
| Injection Site Extravasation | 1 | 4.73 (2.77 - 6.69) | 4.73 ( 0.67 - 33.63 ) | 2.94 | 4.73 ( 0.92 ) | 2.24 ( 0.2 ) |
| Dyspepsia | 1 | 0.66 (-1.3 - 2.62) | 0.66 ( 0.09 - 4.69 ) | 0.18 | 0.66 ( 0.13 ) | -0.6 ( -2.64 ) |
| Anal Incontinence | 1 | 7.85 (5.89 - 9.81) | 7.86 ( 1.11 - 55.84 ) | 5.98 | 7.85 ( 1.52 ) | 2.97 ( 0.93 ) |
| Arachnoid Cyst | 1 | 164.28 (162.32 - 166.25) | 164.46 ( 23.08 - 1172.09 ) | 161.82 | 163.81 ( 31.67 ) | 7.36 ( 5.31 ) |
| Flushing | 1 | 0.61 (-1.35 - 2.57) | 0.61 ( 0.09 - 4.32 ) | 0.25 | 0.61 ( 0.12 ) | -0.72 ( -2.76 ) |

**Supplementary Table 5.** Time-to-onset analysis of Lecanemab related AEs signals using the Weibull distribution test among Non-AD patients.

|  |  |  |  | Weibull Distribution | | | |  |
| --- | --- | --- | --- | --- | --- | --- | --- | --- |
|  | Time to onset (days) | | | Scale Parameter | | Shape Parameter | |  |
| Drug | N | Median (IQR) | Min-Max | α | 95% CI | β | 95% CI | Failure Type |
| Lecanemab | 69 | 30 (14.00 - 59) | 1 - 211 | 41.85 | 30.28 - 53.38 | 0.9 | 0.73 - 1.07 | Early Failure |
